# African swine fever virus pB318L, a trans-geranylgeranyl-diphosphate synthase, negatively regulates cGAS-STING and IFNAR-JAK-STAT signaling pathways

**DOI:** 10.1371/journal.ppat.1012136

**Published:** 2024-04-15

**Authors:** Xiaohong Liu, Hefeng Chen, Guangqiang Ye, Hongyang Liu, Chunying Feng, Weiye Chen, Liang Hu, Qiongqiong Zhou, Zhaoxia Zhang, Jiangnan Li, Xianfeng Zhang, Xijun He, Yuntao Guan, Zhengshuang Wu, Dongming Zhao, Zhigao Bu, Changjiang Weng, Li Huang

**Affiliations:** 1 National African Swine Fever Para-reference Laboratory, State Key Laboratory of Veterinary Biotechnology, Harbin Veterinary Research Institute, Chinese Academy of Agricultural Sciences, Harbin, China; 2 Heilongjiang Provincial Key Laboratory of Veterinary Immunology, Harbin, China; Pirbright Institute, UNITED KINGDOM

## Abstract

African swine fever (ASF) is an acute, hemorrhagic, and severe infectious disease caused by the ASF virus (ASFV). ASFV has evolved multiple strategies to escape host antiviral immune responses. Here, we reported that ASFV pB318L, a trans-geranylgeranyl-diphosphate synthase, reduced the expression of type I interferon (IFN-I) and IFN-stimulated genes (ISGs). Mechanically, pB318L not only interacted with STING to reduce the translocation of STING from the endoplasmic reticulum to the Golgi apparatus but also interacted with IFN receptors to reduce the interaction of IFNAR1/TYK2 and IFNAR2/JAK1. Of note, ASFV with interruption of *B318L* gene (ASFV-intB318L) infected PAMs produces more IFN-I and ISGs than that in PAMs infected with its parental ASFV HLJ/18 at the late stage of infection. Consistently, the pathogenicity of ASFV-intB318L is attenuated in piglets compared with its parental virus. Taken together, our data reveal that *B318L* gene may partially affect ASFV pathogenicity by reducing the production of IFN-I and ISGs. This study provides a clue to design antiviral agents or live attenuated vaccines to prevent and control ASF.

## Author summary

African swine fever virus (ASFV) causes a highly lethal swine disease in many countries, severely affecting the pig industry. Until now, African swine fever has caused substantial economic losses to the world pig industry because of the lack of commercial vaccines and drugs. Therefore, there is an urgent need for efficient commercialized vaccines to prevent and control the disease. It has been reported that the ability of ASFV to escape host antiviral immune responses is closely related to its pathogenicity, and deletion of ASFV virulence-related gene(s) may contribute to the development of attenuated live vaccines. In this study, we found that pB318L reduces the production of IFN-I induced by cGAS-STING and ISGs induced by IFN-α. As a virulence-related gene, interruption of *B318L* significantly attenuated ASFV pathogenicity. Our findings provide a new clue to understand the functions of ASFV-encoded pB318L and its role in viral infection-induced pathogenesis, which might help design antiviral agents or live attenuated vaccines to control ASF.

## Introduction

African swine fever (ASF) is an acute and severe infectious disease caused by ASF virus (ASFV), which can induce approximately 100% mortality in domestic pigs [1]. The ASF epidemic was first reported in Kenya in 1921 [2] and became widespread in Africa, Europe, and East Asia. At present, ASF is a severe plague in Asian countries, including China, Vietnam, and Korea, and has caused huge economic losses. ASFV is the only member of the ASFV family. The ASFV genome varies from 170–193 kb in length, containing more than 150 open reading frames (ORF) encoding 150–200 viral proteins. Some ASFV proteins are required for viral replication [3], and several proteins antagonize the host’s innate immune responses [4]. However, the functions of most ASFV-encoded proteins are still not known.

The innate immune response is the first line of host defense against the invasion of pathogenic microorganisms. Upon pathogenic infection, pattern recognition receptors (PRRs) in host cells recognize pathogen-related molecular patterns (PAMPs) and initiate the production of IFNs, ISGs, inflammatory cytokines, and other antiviral proteins to eliminate the pathogens [5,6]. During ASFV infection, cyclic GMP-AMP synthase (cGAS) recognizes the viral genomic DNA and catalyzes the cyclization reaction of ATP and GTP to form cyclic-GMP-AMP (cGAMP) [7]. Subsequently, cGAMP binds to the intracellular stimulator of IFN genes (STING), causing a conformational change of STING, which then translocates from the endoplasmic reticulum (ER) to the Golgi apparatus to recruit and phosphorylate TANK binding kinase 1 (TBK1) [8, 9]. The phosphorylated TBK1 further recruit interferon regulatory factor 3 (IRF3) to promote its phosphorylation and activation [9]. The activated IRF3 forms a dimer and then translocates to the nucleus to regulate the production of type I interferons (IFN-I). The secreted IFN-I binds to its receptors (IFNAR1 and/or IFNAR2), resulting in the phosphorylation of two JAK kinases (JAK1 and TYK2), which subsequently phosphorylate signal transducers and activators of transcription 1/2 (STAT1/2). The phosphorylated STAT1/2 interacts with IFN regulatory factor 9 (IRF9) to form a heterotrimeric complex, interferon-stimulated gene factor 3 complex (ISGF3), which then enters the nucleus and binds to the IFN-stimulated response elements (ISREs) to induce the expression of many ISGs such as ISG15, ISG54, and ISG56. These ISGs maintain a potent antiviral response in ASFV-infected cells [10,11].

Prenylation is one of the post-translational lipid modifications of many membrane proteins, which plays a crucial role in cellular signaling transduction during viral infections [12]. The mevalonate pathway is one of the essential pathways of cell metabolism, which regulates cholesterol synthesis and prenylation of proteins [13]. The prenylation pathway and the mevalonate pathway are inseparable, and mevalonate is metabolized to isoprenyl pyrophosphate (IPP), which is the direct source for the synthesis of farnesyl diphosphate (FPP) and geranylgeranyl pyrophosphate (GGPP). This process is catalyzed by farnesyl diphosphate synthase (FPPS) and GGPP synthase (GGPPS). Farnesyltransferase (FTase) catalyzes the FPP to the target protein, simultaneously releasing a free diphosphate. Geranyltransferase type 1 and type 2 (GGTsae-I and GGTase-II) use GGPP as a substrate [14]. FTase and GGTase-I recognize the C-terminal CaaX motif in the target proteins, where C is the modified cysteine site [15]. Prenylation can increase the affinity of proteins to membranes, thereby regulating their localization and transport [16].

ASFV *B318L* gene is an ASFV late gene, which encodes the pB318L protein with 318 amino acids (aa) with a molecular weight of 36 kDa. Previous studies showed that ASFV pB318L is a GGPPS [17,18], containing four highly conserved regions unique to these enzymes [19]. So far, the ASFV *B318L* gene is the only prenyltransferase gene of virus origin identified [18]. Moreover, pB318L is expressed in the later stage of infection, which is necessary for the assembly and release of virus particles [20]. It showed that the geranylation of protein plays a vital role in regulating host antiviral immune responses [21].

In this study, we found that pB318L suppresses the production of IFN-I and the expression of ISGs by regulating cGAS-STING and JAK-STAT signaling pathways. ASFV interrupting the *B318L* gene (ASFV-intB318L) significantly reduces the ASFV virulence in piglets compared with its parental ASFV HLJ/18 isolate *in vivo*. Taken together, our findings reveal that ASFV *B318L* gene is a key virulence-related gene that affects the pathogenicity of ASFV, and it functions by reducing the production of IFN-I and ISGs via targeting STING and IFNAR1/2.

## Results

### ASFV pB318L reduces the production of IFN-I and ISGs

pB318L was identified as one of the potent inhibitors of IFN-β promoter activity induced by cGAS-STING [22]. To confirm the role of pB318L during ASFV infection, a recombinant ASFV interrupting the reading frame of the *B318L* gene (ASFV-intB318L) was generated from the highly pathogenic ASFV HLJ/18 strain by homologous recombination (S1A Fig). The whole genome sequence of ASFV-intB318L was sequenced and aligned with that of its paternal virus ASFV HLJ/18 (S1 and S2 Appendix). The results showed that the p72-EGFP cassette was successfully inserted into the upstream of *B438L* gene, and the first and tenth nucleotide of *B318L* gene was deleted (S1 Table). In addition, four other mutations (14720–14721, 19032–19034, 20835–20836, 1415501) exist in the genome of ASFV-intB318L. To eliminate the inaccuracies of whole genome sequencing, these four mutations were further validated by PCR amplification and sequencing. The results showed that there only exists a single nucleotide insertion near the terminus of the *D1133L* gene (S2 and S3 Appendix), which results in a gene sequence that is entirely identical to the *D1133L* gene of the Georgia 2007/1 strain that belongs to genotype II with the HLJ/18 strain. Therefore, it will not affect the function of pD1133L protein and the expression of *D339L* gene downstream of *D1133L* (S3 Appendix).

PCR results showed that the fragments covering the modified region amplified from ASFV-WT and ASFV-intB318L were different in size, and the extra length in the fragment from ASFV-intB318L corresponds to the inserted p72-EGFP cassette (S1B Fig). Western blot results showed that specific bands were detected in porcine alveolar macrophages (PAMs) infected with ASFV-WT but not in the ASFV-intB318L infection group (S1C Fig). Green fluorescence could be observed in PAMs infected with ASFV-intB318L but not ASFV-WT (S1D Fig). These results indicated that *B318L* gene expression was inactivated, and EGFP was successfully inserted in ASFV-intB318L. In addition, the growth kinetics of ASFV-intB318L was evaluated in PAMs, and the replication index of ASFV-intB318L was slightly reduced in PAMs compared to its parental ASFV (S1E Fig).

**Fig 1:**
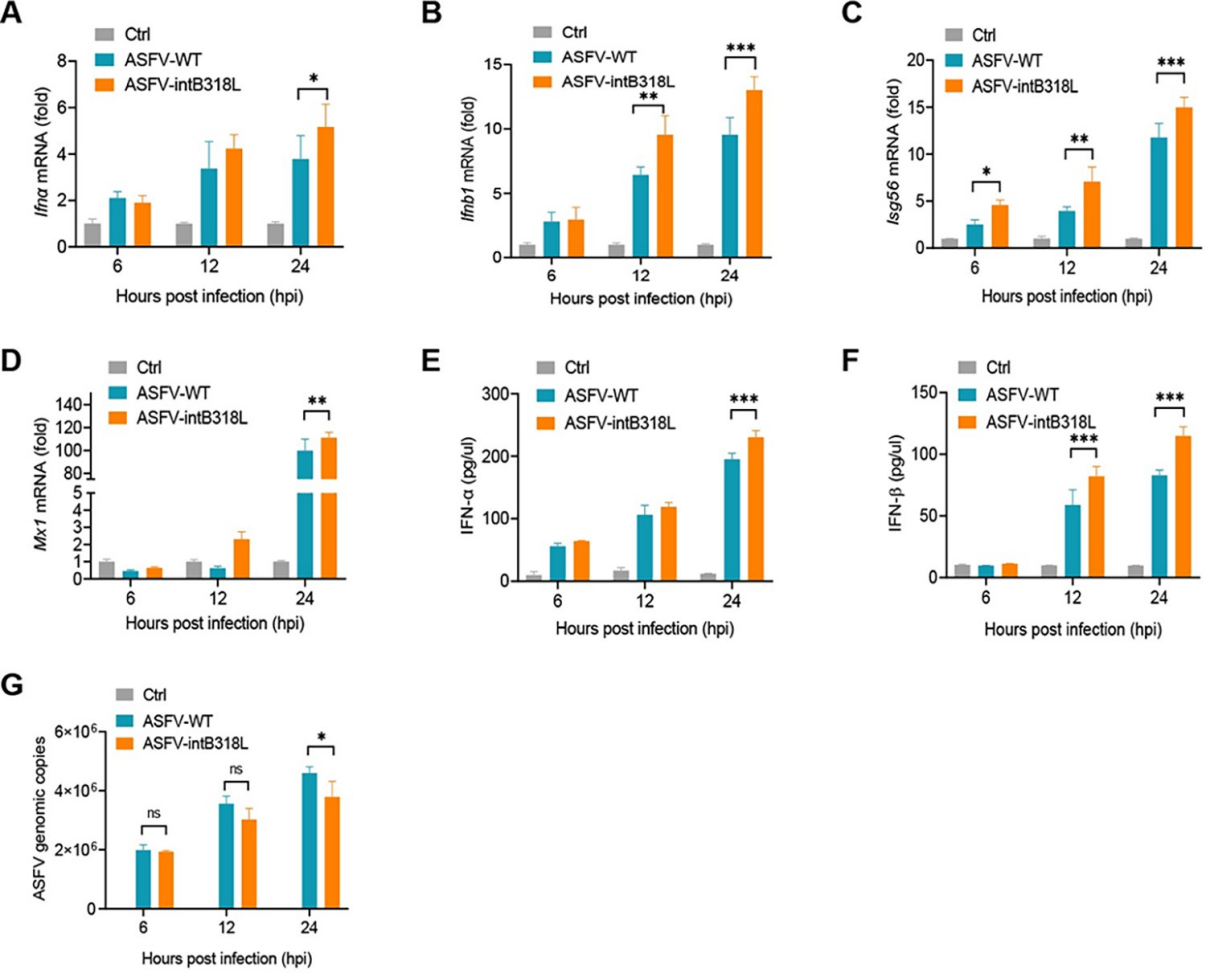
Interruption of *B318L* enhances type I IFN production. **(A-E)** PAMs were mock infected or infected with ASFV-WT or ASFV-intB318L for 6, 12, or 24 h (MOI = 1). The cells were collected to detect the mRNA levels of *Ifna* (A), *Ifnb1* (B), *Isg56* (C), *Mx1* (D), and ASFV genomic copies (G) by qPCR, and the cell supernatants were collected to detect the secreted IFN-α (E) and IFN-β (F) by ELISA. Data are representative of three independent experiments with three biological replicates (mean ± s.d.). * *p* < 0.05, ** *p* < 0.01, *** *p* < 0.001 (one-way ANOVA).

To assess the effect of *B318L* gene interruption on the ASFV infection-induced IFN-I and ISGs, PAMs were infected with ASFV-intB318L or ASFV-WT (multiplicity of infection (MOI) = 1) for 6, 12, or 24 h, and the mRNA and protein levels of several cytokines including IFN-α, IFN-β, ISG56, and MX1 were detected by qPCR and ELISA, respectively. We found that both the mRNA (Fig 1A–1D) and protein levels (Fig 1E and 1F) of IFN-α, IFN-β, ISG56, and MX1 in PAMs induced by ASFV-intB318L infection were higher than those induced by ASFV-WT, especially at 24 hours post-infection (hpi). We also found that the copies of genomic DNA in ASFV-intB318L-infected PAMs were lower than those in ASFV-WT-infected PAMs at 24 hpi (Fig 1G). Taken together, our findings suggest that pB318L plays a critical role in suppressing IFN-I and ISGs production in PAMs during ASFV infection.

**Fig 2:**
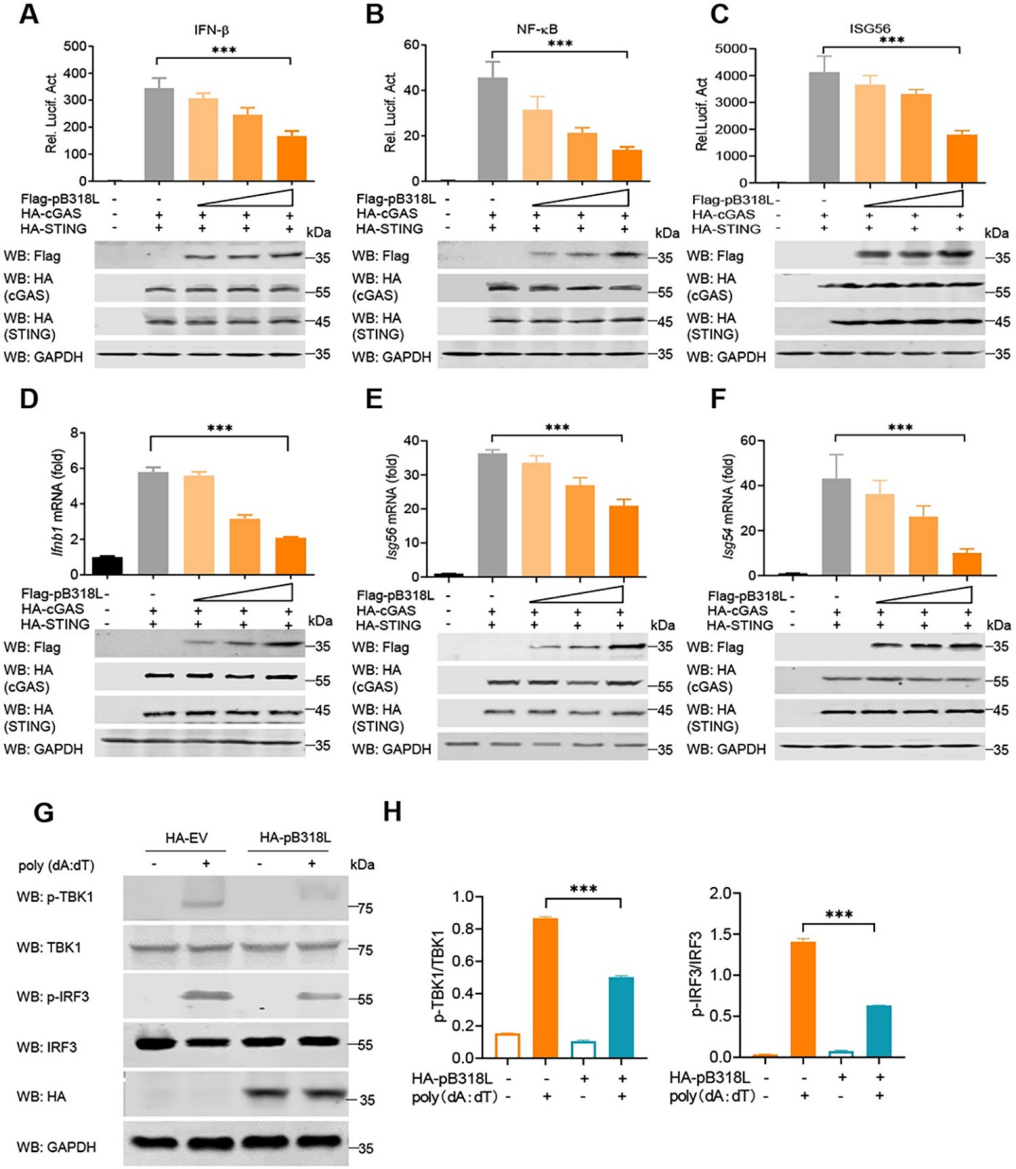
ASFV pB318L reduces type I IFN production and ISGs expression. **(A-C)** HEK293T cells were transfected with an IFN-β- (A), NF-κB- (B) or ISG56- (C) luciferase reporter, a Renilla-TK reporter, and plasmids expressing HA-cGAS and HA-STING, together with increase amounts (0, 100, 200, and 400 ng) of a plasmid expressing Flag-pB318L. Luciferase activities were analyzed at 24 hpt. **(D-F)** HEK293T cells were transfected with plasmids expressing HA-cGAS and HA-STING, together with increased amounts (0, 100, 200, and 400 ng) of a plasmid expressing Flag-pB318L. The mRNA levels of *Ifnb1*, *Isg56*, and *Isg54* were analyzed by qPCR after 24 h. The expressions of cGAS, STING, pB318L, and GAPDH were detected by Western blotting. **(G-H)** HeLa cells were transfected with a plasmid expressing HA-pB318L for 24 h and then treated with poly (dA:dT) for another 24 h. The cells were collected and lysed, and the phosphorylation of TBK1 and IRF3 was detected by Western blotting (G). Quantitations of p-IRF3 and p-TBK1 ratio were analyzed with image J (H). Data are representative of three independent experiments with three biological replicates (mean ± s.d.). Ns, not significantly, * *p* < 0.05, ** *p* < 0.01, *** *p* < 0.001, (one-way ANOVA).

To further explore the regulatory effect of ASFV pB318L on IFN-I production, we tested the effect of overexpressed pB318L on the reporter activities of related genes using the luciferase reporter system. HEK293T cells were co-transfected with increasing doses of a plasmid expressing pB318L, along with an IFN-β, NF-κB, or ISG56 luciferase reporter, an internal control Renilla-TK luciferase, and plasmids expressing cGAS and STING. As shown in Fig 2A–2C, ectopically expressed pB318L reduced the IFN-β, NF-κB, and ISG56 promoter activity induced by coexpressed cGAS and STING in a dose-dependent manner. A plasmid expressing ASFV pI215L or pH171R was also transfected into HEK293T cells as a positive or negative control (S2 Fig) [22]. To further analyze the negative regulation of pB318L on IFN-I gene expression, HEK293T cells were transfected with plasmids expressing cGAS and STING and together with different doses of a plasmid expressing pB318L, and then the mRNA levels of *Ifnb1*, *Isg56*, *Isg54* were detected by qPCR. We found that the ectopic expression of pB318L significantly down-regulated the transcriptional levels of *Ifnb1* (Fig 2D), *Isg56* (Fig 2E), and *Isg54* (Fig 2F) induced by cGAS-STING in a dose-dependent manner. These results indicate that pB318L strongly suppresses the promoter activities of IFN-I-related genes. As TBK1 and IFR3 are vital regulators of IFN-I signaling, we further tested whether pB318L affects the activities of TBK1 and IFR3. As shown in Fig 2G and 2H, poly (dA:dT) (a dsDNA analog that binds to cGAS to active cGAS-STING signaling pathway) strongly induced phosphorylation of TBK1 and IRF3, which were both significantly suppressed by the ectopically expressed pB318L. Together, these results suggest that ASFV pB318L considerably reduces the production of IFN-I induced by cGAS-STING *in vitro*.

### ASFV pB318L reduces STING-mediated IFN production

**Fig 3:**
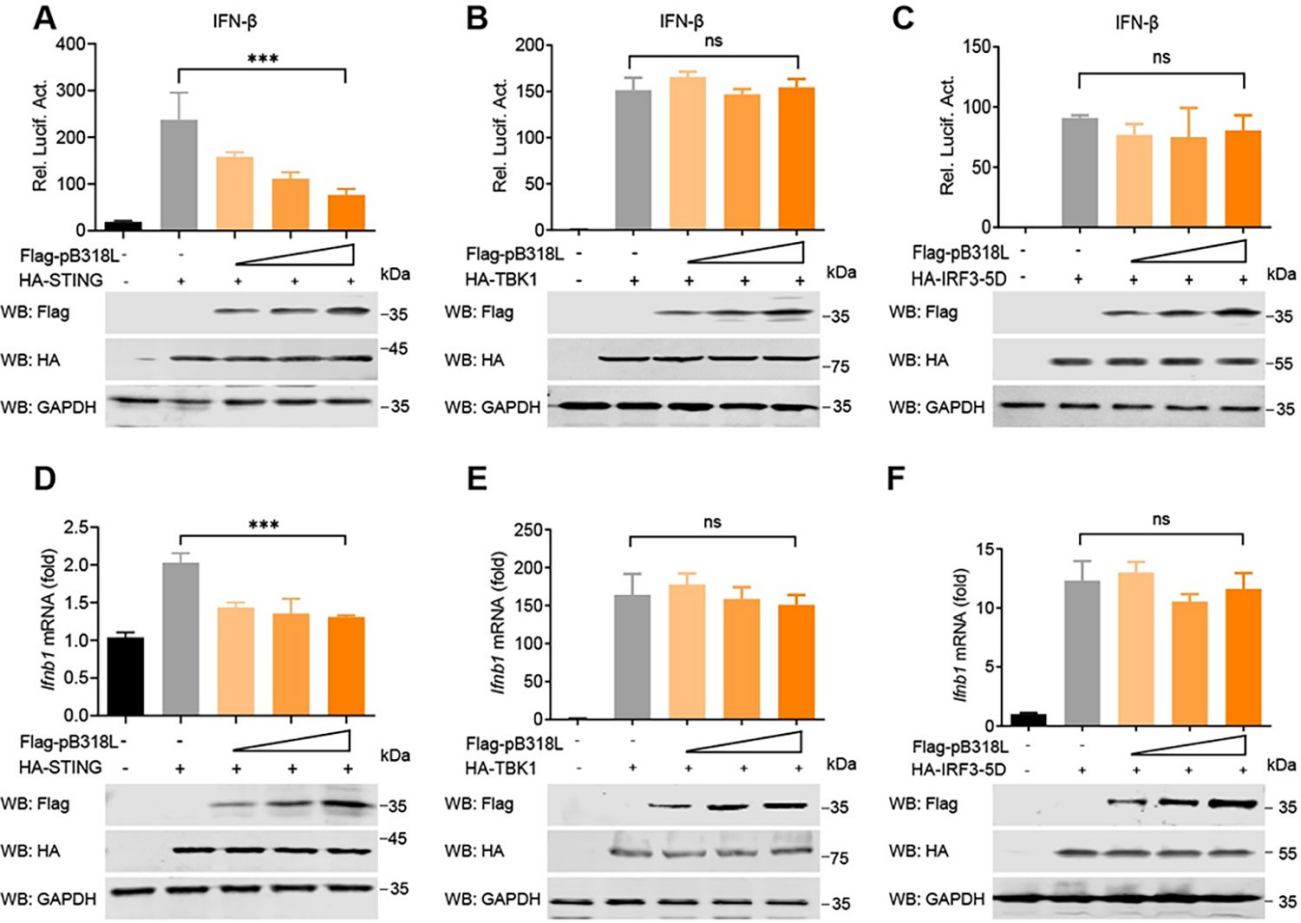
ASFV pB318L reduces STING-mediated IFN production. **(A-C)** HEK293T cells were transfected with an IFN-β luciferase reporter, a Renilla-TK reporter, a plasmid expressing HA-STING (A), HA-TBK1 (B), or HA-IRF3-5D (C), together with increase amounts (0, 100, 200, and 400 ng) of a plasmid expressing Flag-pB318L. The luciferase activities were detected after 24 h. **(D-F)** HEK293T cells were transfected with a plasmid expressing HA-STING (D), HA-TBK1 (E), or HA-IRF3-5D (F), together with increased amounts (0, 100, 200, and 400 ng) of a plasmid expressing Flag-pB318L. The mRNA levels of *Ifnb1* were analyzed by qPCR. The expressions of STING, TBK1, IRF3-5D, pB318L, and GAPDH were detected by Western blotting. Data are representative of three independent experiments with three biological replicates (mean ± s.d.). Ns, not significantly, *** *p* < 0.001 (one-way ANOVA).

To identify the targeting molecules by ASFV pB318L in the cGAS-STING signaling pathway, the key molecules in the cGAS-STING signaling pathway, such as STING, TBK1, and IRF3-5D (a constitutively active IRF3 [23]), were co-expressed with ASFV pB318L in HEK293T cells, respectively. The IFN-β promoter activity was analyzed at 24 hours post-transfection (hpt). We found that ectopically expressed pB318L significantly reduced the activation of the IFN-β promoter reporter induced by STING in a dose-dependent manner (Fig 3A) but not TBK1 and IRF3-5D (Fig 3B–3C). Consistent with these results, pB318L reduced the mRNA levels of *Ifnb1* induced by STING (Fig 3D) but not TBK1 and IRF3-5D (Fig 3E and 3F). Meanwhile, the western blotting results indicated that ASFV pB318L did not affect the expression levels of these indicated proteins. These findings were consistent with the results shown in Fig 2G and 2H, further suggesting that ASFV pB318L functions upstream of TBK1 and may target STING to reduce the production of IFN-I.

### ASFV pB318L interacts with STING and reduces STING translocation to Golgi

**Fig 4:**
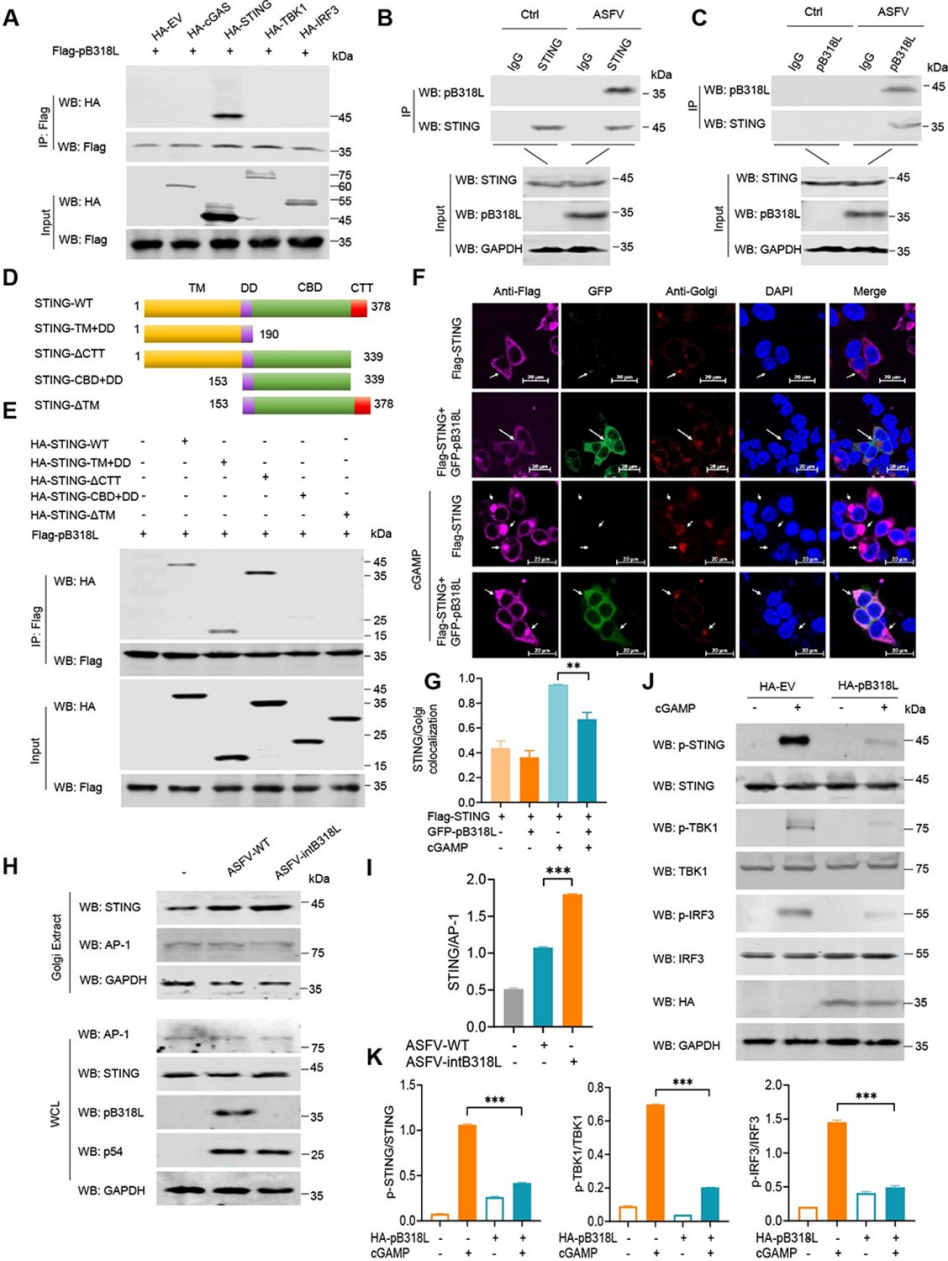
ASFV pB318L interacts with STING and reduces the location of STING on Golgi. **(A)** Co-IP analysis of the interaction between pB318L and key molecules in the cGAS-STING signaling pathway. HEK293T cells were transfected with plasmids expressing Flag-pB318L (2 μg) and HA-tagged cGAS, STING, TBK1, or IRF3 (2 μg/each) as indicated, respectively. Then, cell lysates were incubated with anti-flag (M2) beads and analyzed through Western blotting. **(B-C)** Co-IP analysis of the interaction between ASFV pB318L and endogenous STING in PAMs that were mock infected or infected with ASFV (MOI = 1). Anti-STING was used for the Co-IP in (B), and anti-pB318L was used in (C). **(D)** Construction of plasmids expressing HA-STING and its truncated mutants. **(E)** HEK293T cells were transfected with a plasmid expressing Flag-pB318L (2 μg), along with plasmids expressing HA-STING-WT, HA-STING-TM+DD, HA-STING-ΔCTT, HA-STING-CBD+DD, HA-STING-ΔTM (2 μg/each), respectively. The cells were collected at 24 hpt, and the interactions of STING and its deleted mutants with pB318L were analyzed by Co-IP and Western blotting. **(F-G)** The subcellular localization of GFP-pB318L (1 μg) and Flag-STING (1 μg) in CRL-2843 cells were detected by immunofluorescence microscopy (F). Scale bars, 20 μm. The fluorescence intensities of the images were analyzed using the Zeiss processing system (G). **(H-I)** PAMs were infected with ASFV-WT or ASFV-intB318L (MOI = 1). At 24 hpi, the cells were collected and treated with Golgi Apparatus Enrichment reagent. The levels of STING on Golgi were detected by Western blotting (WCL refers to whole cell lysates) (H). The quantitation ratio of STING on Golgi was analyzed with image J analysis (I). **(J-K)** HeLa cells were transfected with a plasmid expressing HA-B318L (2 μg) for 24 h, and then treated with cGAMP (10 μg/mL) for another 12 h. The cells were collected and lysed, and the phosphorylation levels of STING, TBK1, and IRF3 were detected by Western blotting (J). Quantitation of p-STING, p-TBK1, and p-IRF3 ratio were analyzed with image J (K). Data are representative of three independent experiments with three biological replicates (mean ± s.d.). ** *p* < 0.01. *** *p* < 0.001.

To detect which protein in cGAS-STING signaling is targeted by and interacts with ASFV pB318L, HEK293T cells were transfected with plasmids expressing pB318L and cGAS, STING, TBK1, and IRF3, respectively. Co-IP results showed that pB318L only co-immunoprecipitated with STING but not cGAS, TBK1, or IRF3 (Figs 4A, S3A and S3B). To further evaluate the interaction between pB318L and STING in the context of ASFV infection, PAMs were infected with ASFV, and the cell lysates were immunoprecipitated with anti-STING or anti-pB318L antibodies, respectively. The results showed that ASFV pB318L interacted with endogenous STING in ASFV-infected PAMs (Fig 4B and 4C). To investigate whether pB318L directly binds to STING, a GST pull-down assay was performed using GST-pB318L purified from *E*. *coli* and HA-STING purified from HEK293T cells. We found that STING directly interacts with GST-pB318L but not GST (S3C Fig). These results suggest that pB318L directly interacts with STING.

Porcine STING contains an N-terminal transmembrane domain (TM) (aa 1–153), a dimerization domain (DD) (aa 154–190), a CDN binding domain (CBD) (aa 191–339), a C-terminal TBK1 and IRF3 binding domain (CTT) (aa 340–378). To identify which domain of STING is necessary for its interaction with pB318L, four truncated mutants of STING (STING-TM+DD, STING-ΔCTT, STING-DD+CBD, STING-ΔTM) were constructed (Fig 4D). Domain-mapping studies revealed that pB318L interacted with STING-WT, STING-TM+DD and STING-ΔCTT, but not STING-DD+CBD and STING-ΔTM, suggesting that the TM domain of STING is required for its interaction with pB318L (Fig 4E). Based on analysis of the homologous enzyme of pB318L [18], we found that pB318L also contains a transmembrane domain (TM) and four conserved domains. Six plasmids expressing the truncated mutants of pB318L were constructed (S3D Fig), and domain-mapping studies revealed that except for the TM domain, all other mutants of pB318L interacted with STING (S3E Fig).

STING is localized to organelles through its transmembrane region, and the translocation of STING from ER to Golgi apparatus is necessary for its activation in cGAS-STING signaling [8]. To explore the effect of pB318L on STING localization, confocal immunofluorescence and Golgi apparatus isolation were performed. We found that cGAMP (an endogenous second messenger that activates STING) promoted the translocation of STING from ER to the Golgi apparatus, which was strongly suppressed by pB318L (Fig 4F and 4G). Consistent with these results, pB318L also reduced the location of STING on the Golgi apparatus induced by diABZI, a STING agonist (S3F and S3G Fig). To further confirm the inhibition effect of *B318L* during ASFV infection, PAMs were infected with ASFV-WT or ASFV-intB318L, and then the Golgi apparatus was extracted to analyze STING levels. We noticed more STING on the Golgi apparatus in ASFV-intB318L-infected PAMs compared with ASFV-WT infection (Fig 4H and 4I). The location of STING on the Golgi apparatus recruits and phosphorylates TBK1, leading to the phosphorylation of STING and IRF3 [24]. Consistent with our expectations, we found that ectopically expressed pB318L reduced the phosphorylation of STING, TBK1, and IRF3 induced by cGAMP (Fig 4J and 4K). Together, these results indicate that pB318L interacts with the transmembrane region of STING to reduce its translocation from ER to the Golgi apparatus.

### ASFV pB318L reduces IFN-I production dependent on its enzymatic activity

**Fig 5:**
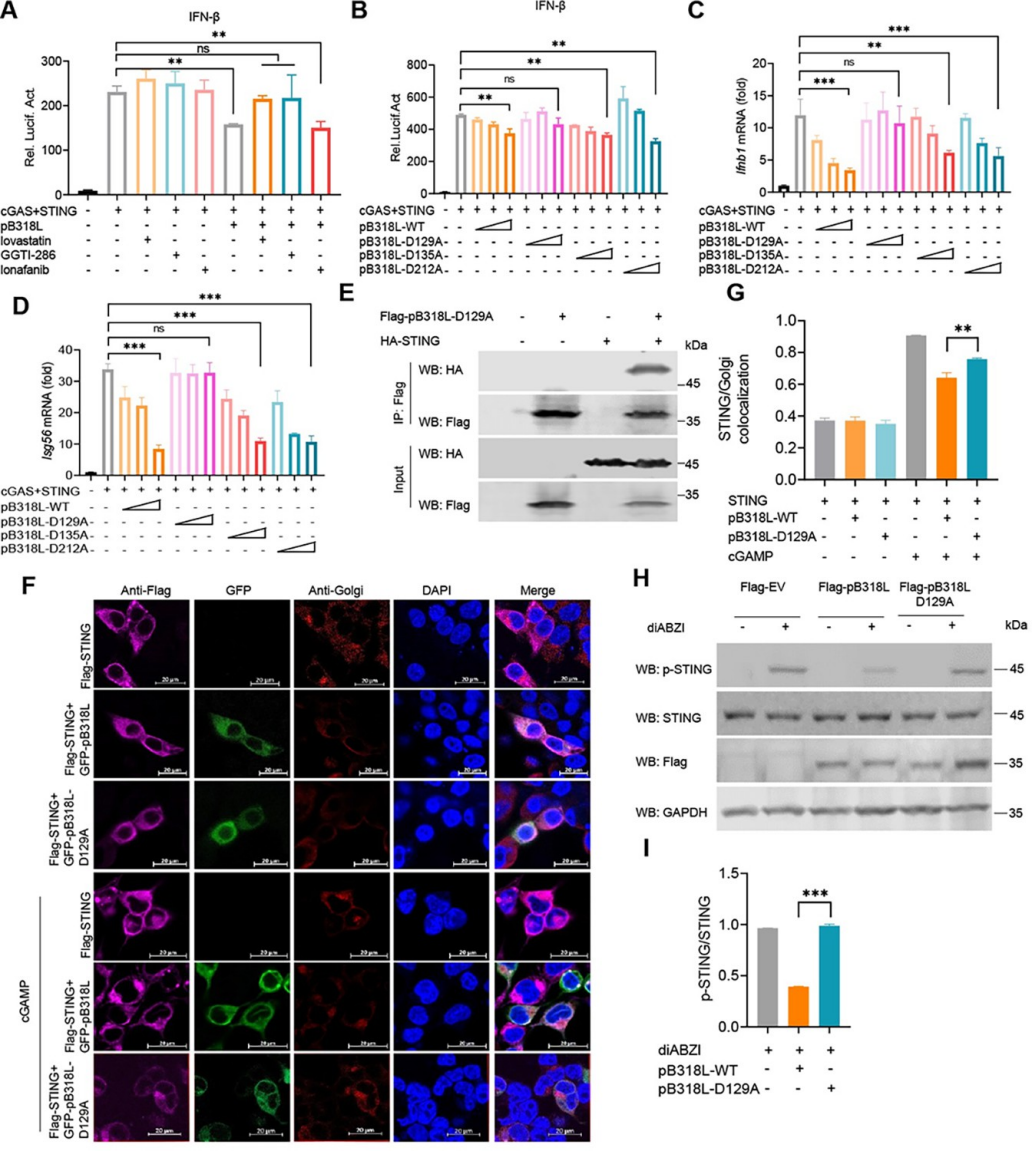
Asp129 of ASFV pB318L affects its inhibition of STING phosphorylation and translocation. **(A)** HEK293T cells were transfected with an IFN-β luciferase reporter, a Renilla-TK reporter, and plasmids expressing HA-cGAS and HA-STING, together with a plasmid expressing Flag-pB318L. After 24 h, the cells were treated with Lovastatin, Lonfarnib, GGTI-286 for 12 h, then the luciferase activities were detected. **(B)** HEK293T cells were transfected with an IFN-β luciferase reporter, a Renilla-TK reporter, and plasmids expressing HA-cGAS and HA-STING, together with increased amount (100 ng, 200 ng, 400 ng) of a plasmid expressing Flag-pB318L-WT or Flag-pB318L-Mut, the luciferase activities were detected after 24 h. **(C-D)** HEK293T cells were transfected with plasmids expressing HA-cGAS and HA-STING, together with increase amount (100 ng, 200 ng, 400 ng) of a plasmid expressing Flag-pB318L-WT (100 ng, 200 ng, 400 ng) or Flag-pB318L-Mut, the mRNA levels of *Ifnb1* and *Isg56* were analyzed by qPCR. **(E)** HEK293T cells were transfected with plasmids expressing Flag-pB318L-D129A and HA-STING. Co-IP analysis was performed to detect the interaction between pB318L-D129A and STING after 24 h. **(F-G)** CRL2843 cells were transfected with plasmids expressing HA-STING and GFP-pB318L or GFP-pB318L-D129A as indicated. At 24 hpt, the cells were stimulated with cGAMP (10 μg/mL) for another 12 h. The subcellular localization of STING was visualized by immunofluorescence microscopy. Scale bars, 20 μm (F). The fluorescence intensity of STING was analyzed using the Zeiss processing system (G). **(H-I)** HeLa cells were transfected with a plasmid expressing Flag-pB318L or Flag-pB318L-D129A for 24 h, and then treated with the STING agonist for another 6 h. The cells were collected and lysed, and the phosphorylation of STING was detected by Western blotting (H). Quantitation of p-STING/STING ratio was analyzed with Image J (I). Data are representative of three independent experiments with three biological replicates (mean ± s.d.). Ns, not significantly, ** p < 0.01, *** p < 0.001 (one-way ANOVA).

It has been reported that pB318L is a geranylgeranyl diphosphate synthase [18]. Lovastatin is an inhibitor of HMG-CoA reductase, which reduces the production of mevalonate, thereby inhibiting the prenylation modification. Lonafarnib is an inhibitor of FTase, and GGTI-286 is an inhibitor of GGTase-I. To test whether ASFV pB318L negatively regulates IFN-I production through its geranylgeranyl diphosphate synthase activity, HEK293T cells were transfected with IFN-β luciferase reporter and plasmids expressing STING and pB318L as indicated in the presence of three prenylation inhibitors, respectively. We found that both Lovastatin and GGTI-286 could rescue the suppression of cGAS-STING-induced IFN-β promoter activity by pB318L, while Lonafarnib couldn’t (Fig 5A). These results suggest that the geranylgeranyl diphosphate synthase activity of pB318L is required for its suppressing IFN-I production.

Based on the amino acids of the active center of the homologous structure of pB318L [25], we analyzed the active center of pB318L and predicted that D129, D135, and D212 may affect the enzyme activity. To map the key amino acid residues that are related to pB318L enzymatic activity on reducing IFN-І production, three plasmids expressing ASFV pB318L mutants such as pB318L-D129A, pB318L-D135A, and pB318L-D212A, were generated. The results showed that except for pB318L-D129A, pB318L-WT and all the other mutants reduced both the promoter activity of IFN-β (Fig 5B) and the mRNA expression levels of *Ifnb1* and *Isg56* (Fig 5C and 5D) induced by expressed cGAS-STING, which indicates that D129 of ASFV pB318L plays an important role in reducing IFN-I production. Additionally, we found that although pB318L-D129A still interacted with STING, but it lost its inhibition of the Golgi apparatus translocation and phosphorylation of STING (Fig 5E–5I). RhoA is a type of Rho GTPase that has been shown to be isoprenylated [26]. To demonstrate that Asp129 is required for the enzyme activity of pB318L, the effect of B318L-WT and pB318L-D129A on the isoprenylation of RhoA was detected. We found that pB318L promoted the isoprenylation of RhoA; however, the effect of B318L-D129A is relatively weak (S3H Fig), suggesting that Asp129 affects but not determines the enzyme activity of pB318L. Taken together, our findings suggest that the enzymatic activity of pB318L is related to its function in suppressing IFN-I production, and the Asp129 plays a key role in the process.

### ASFV pB318L reduces the phosphorylation and translocation of STATs

**Fig 6:**
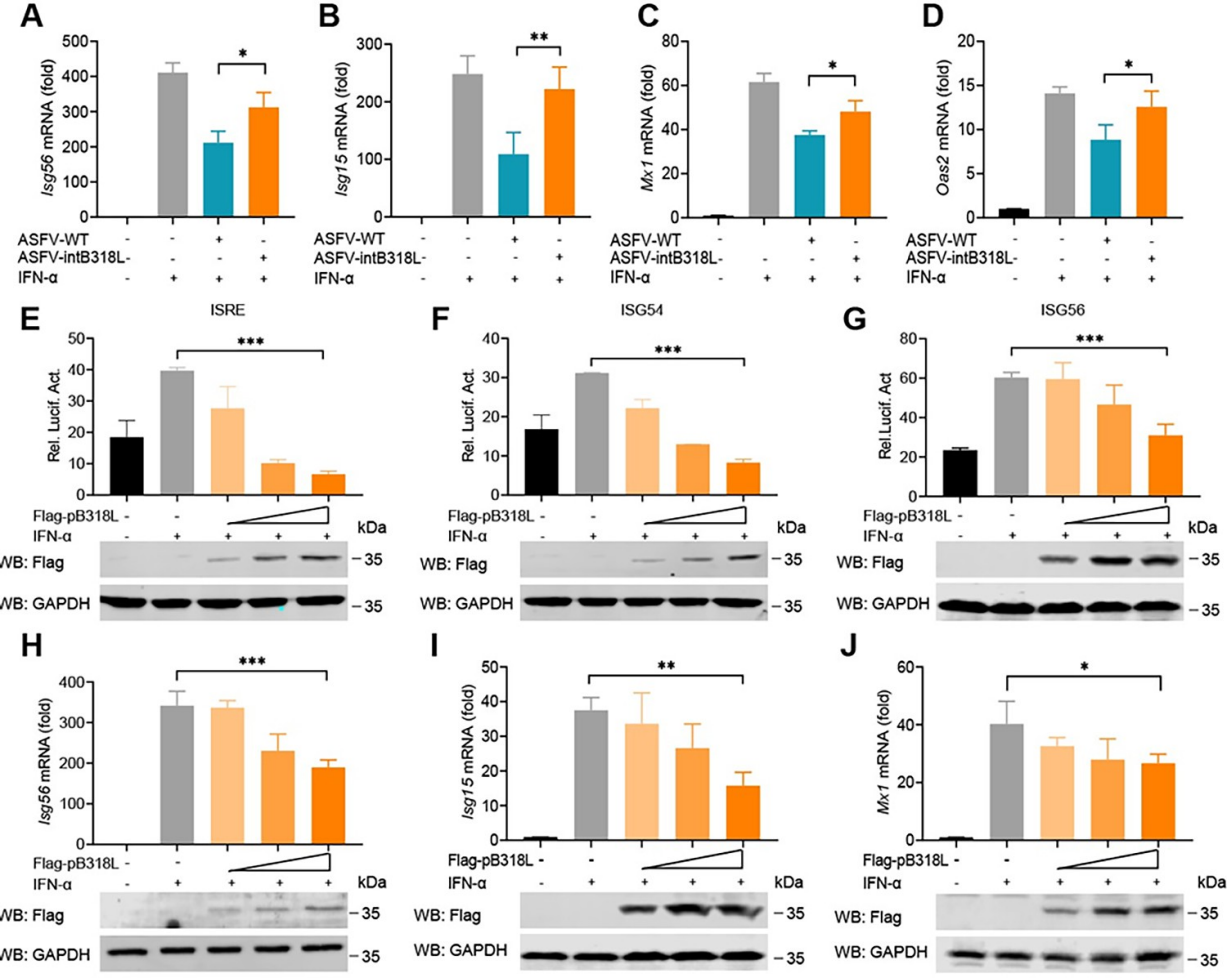
ASFV pB318L reduces the production of ISGs. **(A-D)** PAMs were infected with ASFV-WT or ASFV-intB318L for 24 h (MOI = 1) and then treated with IFN-α (1 μg/mL) for another 12 h. The mRNA levels of *Isg56* (A), *Isg15* (B), *Mx1* (C), and *Oas2* (D) were analyzed by qPCR. (**E-G)** HEK293T cells were co-transfected with ISRE-luciferase (E), ISG54-luciferase (F), ISG56-luciferase (G) reporters, and a Renilla-TK reporter along with different doses of a plasmid (0, 100, 200, 400 ng) expressing Flag-pB318L. At 24 hpt, the cells were treated with IFN-α (1 μg/mL) for another 12 h. The cells were collected to detect the luciferase activity. The expressions of pB318L and GAPDH were analyzed by Western blotting. **(H-J)** Different doses of a plasmid (0, 100, 200, 400 ng) expressing Flag-pB318L were transfected into HEK293T cells for 24 h, and then the cells were stimulated with IFN-α (1 μg/mL) for 12 h. The mRNA levels of *Isg56* (H), *Isg15* (I), and *Mx1* (J) were analyzed by qPCR. Data are representative of three independent experiments with three biological replicates (mean ± s.d.). * *p* < 0.05, ** *p* < 0.01, *** *p* < 0.001 (one-way ANOVA).

We noticed that ASFV pB318L also reduces the mRNA of *Isg54* and *Isg56* in HEK293T cells (Fig 2E and 2F). We next explored whether pB318L affects the expression of ISGs during ASFV infection. PAMs were infected with ASFV-WT or ASFV-intB318L for 24 h (MOI = 1) and then treated with IFN-α for an additional 12 h. The mRNA levels of several ISGs were detected by qPCR. We found that ASFV-WT infection strongly suppressed the mRNA levels of *Isg56*, *Isg15*, *Mx1*, and *Oas2* induced by IFN-α. In contrast, the inhibitory effect was obviously attenuated upon ASFV-intB318L infection (Fig 6A–6D). To exclude the possibility that the enhanced ISG expressions during ASFV-intB318L infection were due to the higher viral load compared to the ASFV-WT, we detected the mRNA expression of ASFV p72 protein. The results showed that the mRNA level of ASFV p72 in ASFV-intB318L-infected PAMs was significantly lower than that of ASFV-WT in the presence of IFN-α (S4 Fig). Similarly, overexpressed pB318L significantly reduced both the promoter activities of ISRE, ISG54, and ISG56 (Fig 6E–6G) and the mRNA levels of *Isg56*, *Isg15*, and *Mx1* triggered by IFN-α in a dose-dependent manner (Fig 6H–6J).

**Fig 7:**
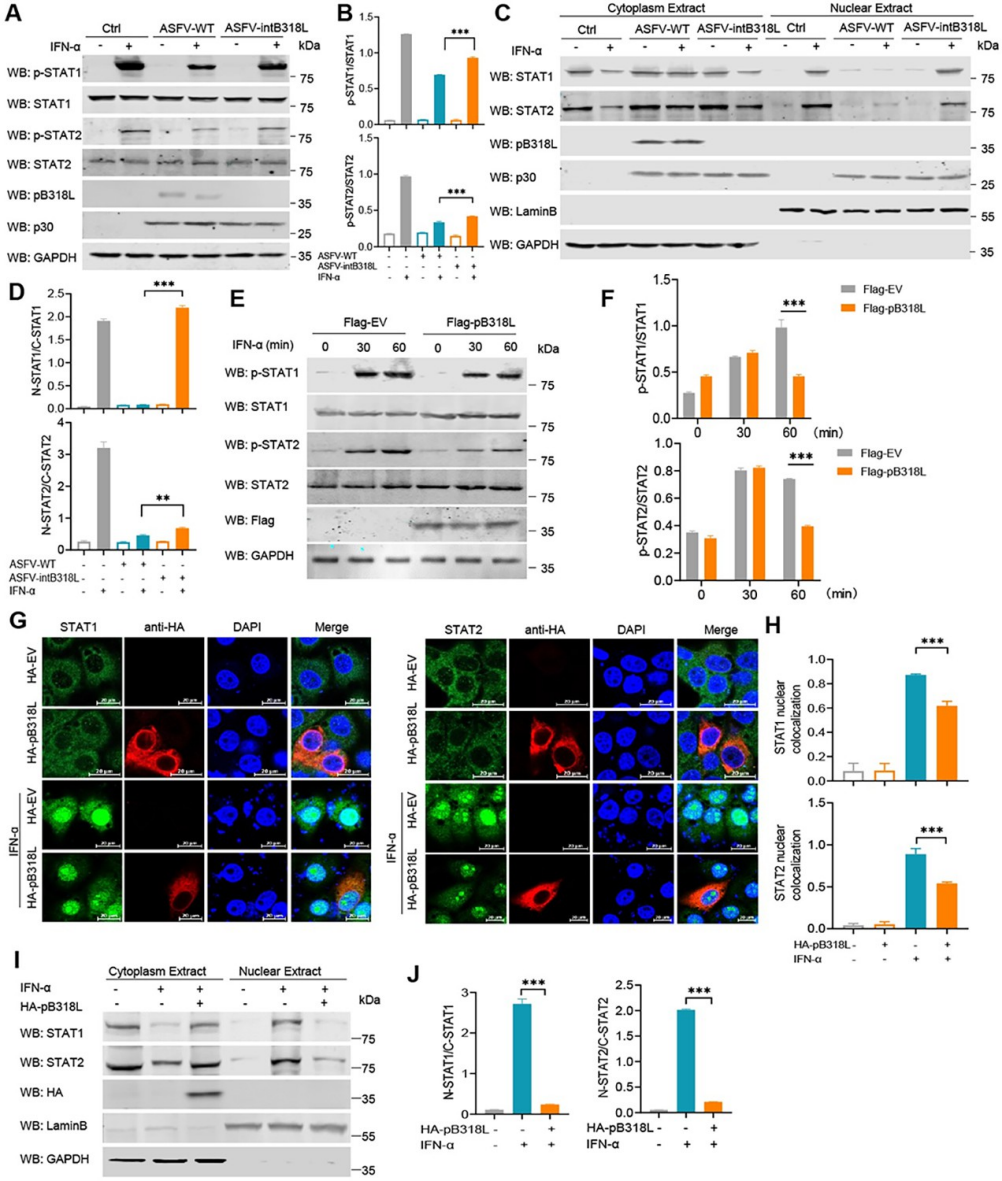
ASFV pB318L reduces phosphorylation and translocation of STATs. **(A-B)** PAMs were infected with ASFV-WT (MOI = 1) or ASFV-intB318L (MOI = 1) for 24 h and then treated with or without IFN-α (1 μg/mL) for 12 h. The cells were collected to test the expressions of STAT1, STAT2, pB318L, p30, GAPDH, and the phosphorylation of STAT1 and STAT2 (A). Quantitation of p-STAT1 and p-STAT2 ratio was analyzed with image J (B). **(C-D)** PAMs were infected with ASFV-WT (MOI = 1) or ASFV-intB318L (MOI = 1) for 24 h and then treated with IFN-α (1 μg/mL) for 12 h. The cells were harvested and processed using nuclear and cytoplasmic extraction reagents to extract the cytoplasmic and nuclear fractions and detect the distribution of STAT1 and STAT2 in the cytoplasm and nucleus, respectively (C). Quantitation of STAT1 and STAT2 in the nuclear ratio was analyzed with Image J (D). **(E-F)** HEK293T cells were transfected with a plasmid expressing Flag-pB318L or empty vector for 24 h. Then, the cells were treated with IFN-α (1 μg/mL) for 30 or 60 min. The cells were collected to analyze the phosphorylation of STAT1 and STAT2 (E). Quantitation of p-STAT1 and p-STAT2 ratios were analyzed with Image J (F). **(G-H)** CRL-2843 cells were transfected with a plasmid expressing HA-pB318L or an empty vector for 24 h, and the cells were stimulated with IFN-α (1 μg/mL) for 60 min. The localizations of STAT1 or STAT2 and pB318L were detected by immunofluorescence microscopy. Scale bars, 20 μm (G). The green fluorescence intensities of the images were analyzed using the Zeiss processing system (H). **(I-J)** HEK293T cells were transfected with a plasmid expressing Flag-pB318L or an empty vector for 24 h. Then, the cells were treated with IFN-α (1 μg/mL) for 60 min. The cells were harvested and processed using nuclear and cytoplasmic extraction reagents to detect the distribution of STAT1 and STAT2 in the cytoplasm and nucleus, respectively (I). Quantitation of STAT1 and STAT2 ratios were analyzed with Image J (J). Data are representative of three independent experiments with three biological replicates (mean ± s.d.). * *p* < 0.05, 0.001 < ** *p* < 0.01, *** *p* < 0.001, (one-way ANOVA).

To examine whether the phosphorylation and subcellular localization of STAT1 and STAT2 are affected by the ASFV pB318L, PAMs were infected with ASFV-WT or ASFV-intB318L and then treated with IFN-α. We found that ASFV-WT infection significantly reduced the phosphorylation and nuclear translocation of STAT1 and STAT2 induced by IFN-α. ASFV-intB318L infection also obviously suppressed the phosphorylation and nuclear translocation of STAT1 and STAT2; the inhibitory effect was significantly lower than that of ASFV-WT infection (Fig 7A–7D). Consistently, ectopically expressed pB318L also significantly reduced the phosphorylation (Fig 7E and 7F) and nuclear translocation (Fig 7G–7J) of STAT1 and STAT2 *in vitro*. These results indicate that ASFV pB318L reduces STAT1/2 phosphorylation and nuclear translocation during ASFV infection.

### ASFV pB318L targets IFNAR1 and IFNAR2 to inhibit IFN-I signaling pathway

**Fig 8:**
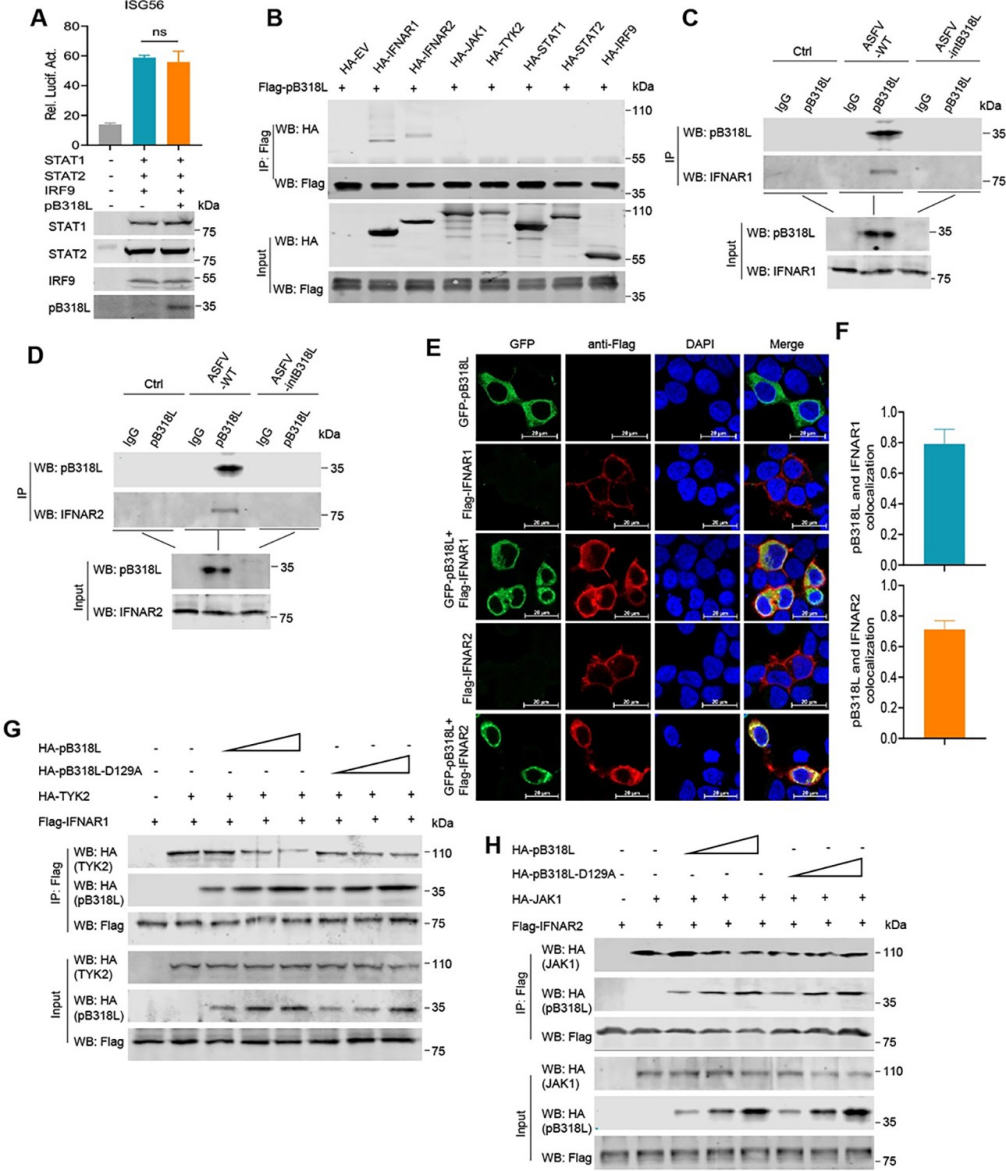
ASFV pB318L interacts with IFNAR1/2 to inhibit IFN signaling pathway. **(A)** HEK293T cells were co-transfected with plasmids expressing pB318L and STAT1, STAT2, IRF9, together with ISG56-luciferase and Renilla-TK, at 24 hpt the cells were collected to analyze the luciferase activity. **(B)** HEK293T cells were transfected with a plasmid expressing Flag-pB318L, along with a plasmid expressing HA-tagged IFNAR1, IFNAR2, JAK1, TYK2, STAT1, STAT2, and IRF9, respectively. At 24 hpt, Co-IP was performed to detect the interaction between pB318L and molecules in the IFNAR-JAK-STAT signaling pathway. **(C-D)** Co-IP analysis of the interaction between pB318L and endogenous IFNAR1/IFNAR2 in PAMs that were mock infected or infected with ASFV or ASFV-intB318L. **(E)** CRL-2843 cells were transfected with plasmids expressing Flag-IFNAR1 or Flag-IFNAR2 and GFP-pB318L. At 24 hpt, the localizations of pB318L, IFNAR1, and IFNAR2 were detected by immunofluorescence microscopy. Scale bars, 20 μm. **(F)** Pearson correlation coefficient between pB318L and IFNAR1/2. **(G)** HEK293T cells were co-transfected with different plasmids expressing HA-pB318L, HA-TYK2, and Flag-IFNAR1 as indicated. Co-IP was performed to detect the interactions among IFNAR1, TYK2 and pB318L. **(H)** HEK293T cells were co-transfected with plasmids expressing HA-pB318L, HA-JAK1, and Flag-IFNAR2 as indicated. Co-IP was performed to detect the interactions among IFNAR2, JAK1, and pB318L. Data are representative of three independent experiments with three biological replicates (mean ± s.d.). *** p < 0.001, (one-way ANOVA).

To further investigate how pB318L represses the transcription of ISGs, HEK293T cells were transfected with plasmids expressing one of ISGF3 components, pB318L and an ISG56 reporter. The results showed that pB318L did not reduce ISG56 reporter activation mediated by the ISGF3 complex (Fig 8A), suggesting that pB318L targets the upstream components of ISGF3 complex in the IFN signaling pathway. To further explore the key step targeted by pB318L in the IFN signaling pathway, HEK293T cells were co-transfected with a plasmid expressing pB318L and a plasmid encoding IFNAR1, IFNAR2, JAK1, TYK2, STAT1, STAT2, and IRF9, respectively. Co-IP results showed that pB318L co-immunoprecipitated with IFNAR1 and IFNAR2 but not other proteins (Figs 8B, S5A and S5B). To further evaluate the interaction between pB318L and IFNAR1 or IFNAR2 during ASFV infection, PAMs were infected with ASFV-WT or ASFV-intB318L, and the cell lysates were immunoprecipitated with anti-pB318L or anti-IFNAR1/2 antibody. We found that ASFV pB318L interacted with endogenous IFNAR1 and IFNAR2 in the ASFV-WT-infected PAMs, not in ASFV-intB318L-infected PAMs (Figs 8C, 8D, S5C and S5D).

Furthermore, we analyzed the localizations of pB318L, IFNAR1, and IFNAR2 in CRL-2843 cells and found that pB318L colocalization with both IFNAR1 and IFNAR2 in the cell membrane. Of note, pB318L colocalized with IFNAR2 and formed foci-like structures in the cytoplasm (Fig 8E and 8F). Consistent with these results, GST pull-down assay showed that GST-pB318L interacted directly with IFNAR1/IFNAR2 *in vitro* (S5E and S5F Fig). To map the binding domains of pB318L to IFNAR1/2, IFNAR1/2 was co-expressed with pB318L or its truncated mutants. We found that only the TM domain is not involved in the interaction with IFNAR1/2 (S5G and S5H Fig). It is worth noting that overexpression of pB318L significantly reduced the interactions of TYK2-IFNAR1 and JAK1-IFNAR2 (Fig 8G and 8H). In summary, these results indicate that ASFV pB318L interacts with IFNAR1 and IFNAR2 to reduce their recruitment of downstream JAK1 and TYK2 kinases, thereby decreasing the production of ISGs.

### The Inhibition of ISGs production by ASFV pB318L depends on its enzymic activity

**Fig 9:**
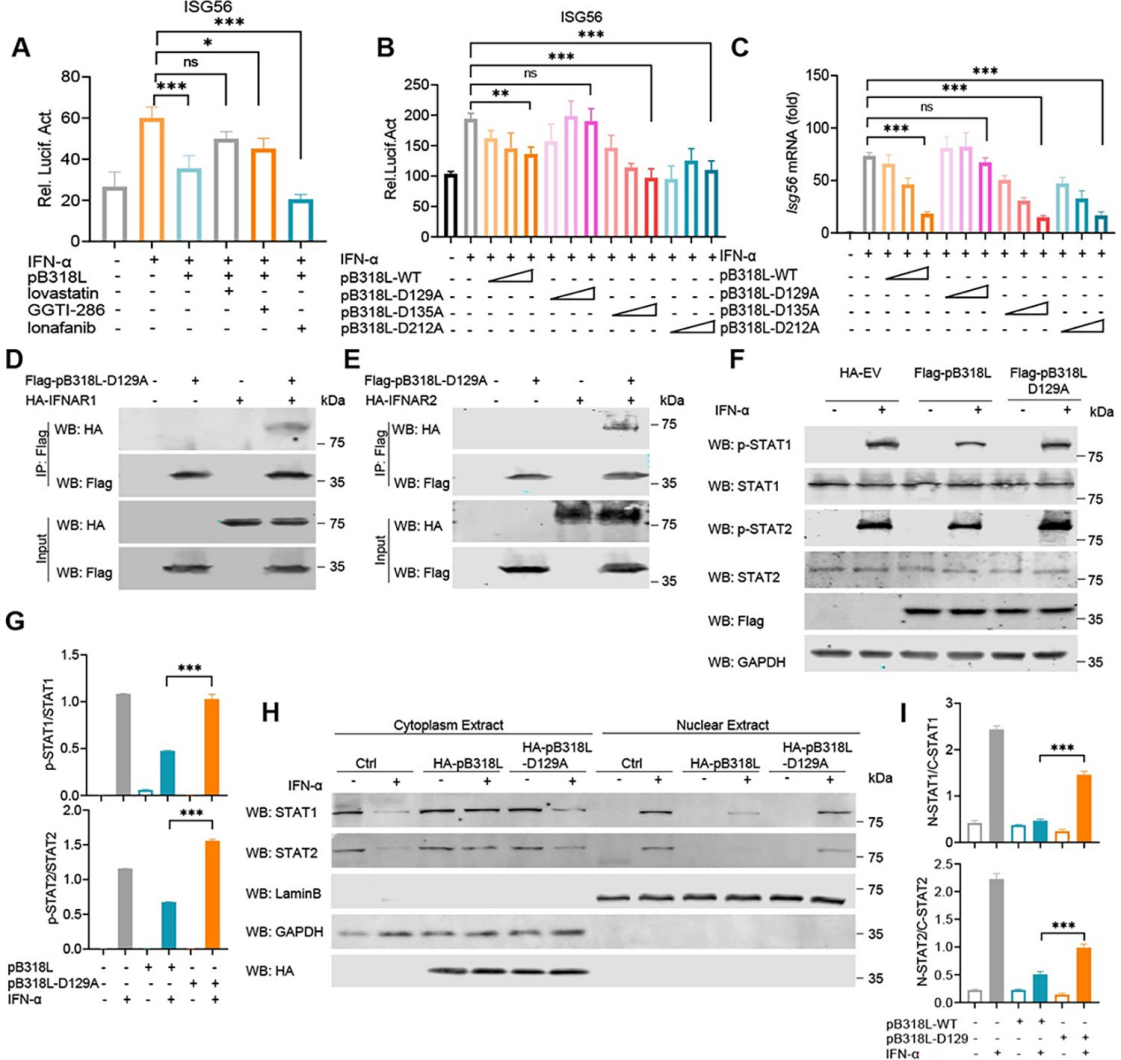
Asp129 of ASFV pB318L affects its inhibition of ISGs expression. **(A)** HEK293T cells were transfected with an ISG56 luciferase reporter and a Renilla-TK reporter, together with a plasmid expressing Flag-pB318L. After 24 h, the cells were treated with IFN-α (1 μg/mL) for 12 h, and then treated with Lovastatin, Lonfarnib, GGTI-286 for another 12 h. After that, the luciferase activities were analyzed. **(B)** HEK293T cells were transfected with an ISG56 luciferase reporter and a Renilla-TK reporter, together with increased amounts (100 ng, 200 ng, 400 ng) of a plasmid expressing Flag-pB318L-WT (100 ng, 200 ng, 400 ng) or Flag-pB318L-Mut. After 24 h, the cells were treated with IFN-α (1 μg/mL) for 12 h, and the luciferase activities were then detected. **(C)** HEK293T cells were transfected with increased amounts (100 ng, 200 ng, 400 ng) of a plasmid expressing Flag-pB318L-WT or Flag-pB318L-Mut, the mRNA level of *Isg56* was analyzed by qPCR. **(D-E)** HEK293T cells were transfected with plasmids expressing Flag-pB318L-D129A and HA-IFNAR1 or HA-IFNAR2 as indicated. Co-IP analysis was performed to detect the interaction between pB318L-D129A and IFNAR1/IFNAR2 after 24 h. **(F-G)** HEK293T cells were transfected with a plasmid expressing Flag-pB318L or Flag-pB318L-D129A for 24 h, and then treated with IFN-α (1 μg/mL) for another 60 min. The cells were collected and lysed, and the phosphorylation levels of STAT1 and STAT2 were detected by Western blotting (F). Quantitation of p-STAT1 and p-STAT2 ratios were analyzed with Image J (G). **(H-I)** HEK293T cells were transfected with plasmids expressing HA-pB318L or HA-pB318L-D129A for 24 h, and the cells were then stimulated with IFN-α (1 μg/mL) for another 12 h. The cells were harvested and processed using nuclear and cytoplasmic extraction reagents to extract the cytoplasmic and nuclear fractions and detect the distribution of STAT1 and STAT2 in the cytoplasm and nucleus, respectively (H). Quantitation of STAT1 and STAT2 in nuclear ratio was analyzed with Image J (I). Data are representative of three independent experiments with three biological replicates (mean ± s.d.). Ns, not significantly, * *p* < 0.05, ** *p* < 0.01, *** *p* < 0.001, (one-way ANOVA).

To detect whether the inhibition of ISGs production by pB318L still depends on its enzymatic activity, HEK293T cells were pretreated with inhibitors Lovastatin, GGTI-286 or Lonafarnib, then stimulated with IFN-α, and the ISG56 reporter activity was detected. The results showed that both Lovastatin and GGTI-286, but not Lonafarnib, strongly rescued the inhibitory effect of pB318L on ISG56 promoter activity induced by IFN-α (Fig 9A). Consistently, pB318L-D129A, but not pB318L-D135A and pB318L-D212A, completely lost its inhibitory effect on the promoter activity and the upregulation of mRNA expression of *Isg56* (Fig 9B and 9C). In addition, we noticed that pB318L-D129A still interacted with IFNAR1/IFNAR2, but it failed to reduce the phosphorylation and nuclear translocation of STAT1 and STAT2 induced by IFN-α (Fig 9D–9I). Taken together, our results suggest that inhibition of IFN-α-mediated ISGs production by pB318L is related to its GGPPS enzymatic activity and that Asp129 plays a role in supporting the pB318L enzyme function.

### The reading frame interruption of *B318L* gene attenuates the pathogenicity of ASFV HLJ/18

**Fig 10:**
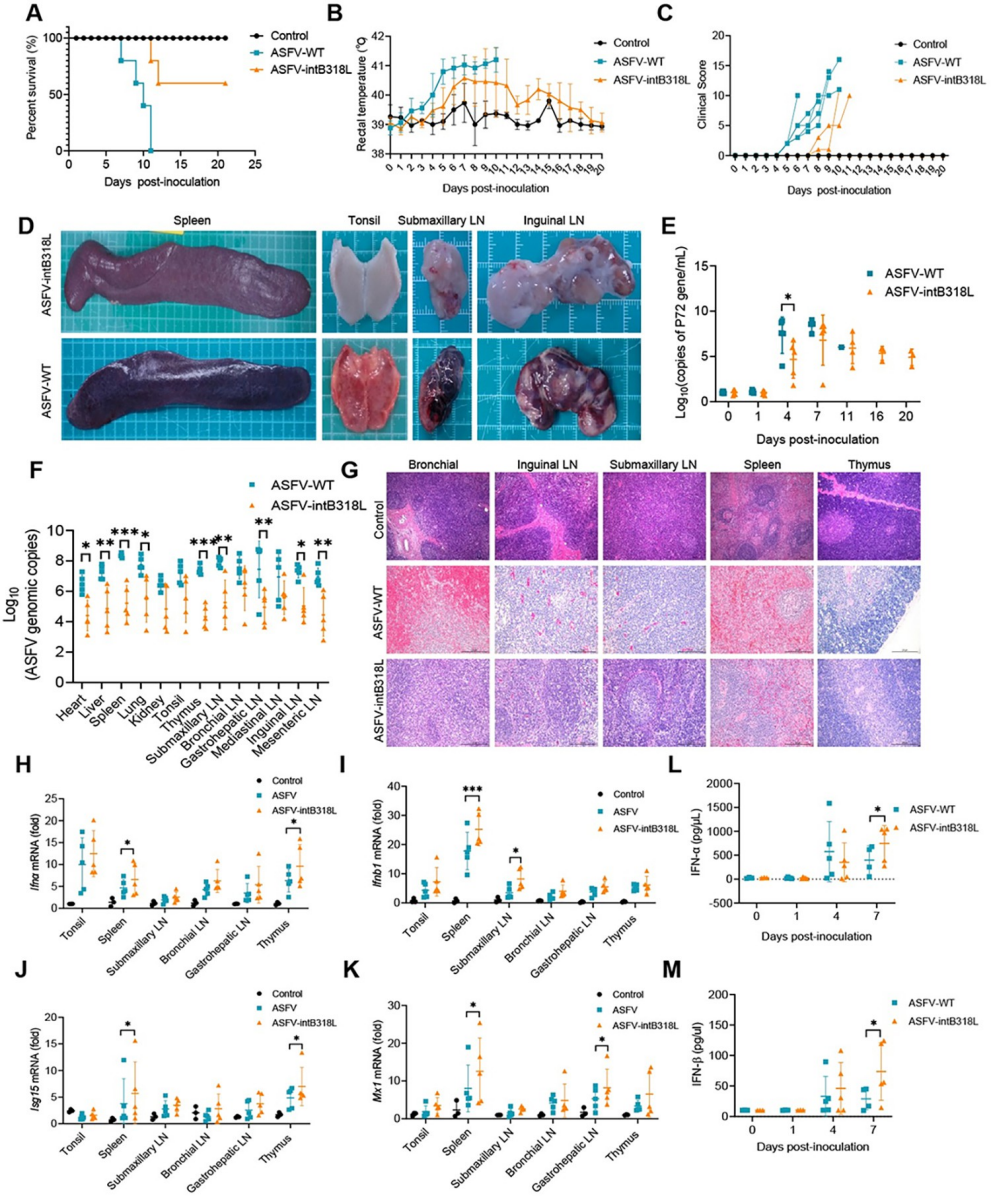
ASFV-intB318L is attenuated in pigs. **(A-C)** Body survival (A), temperatures (B), and clinical score (C) of pigs intramuscularly (IM) inoculated with PBS (n = 3), 10^2.5^ HAD_50_ ASFV-intB318L (n = 5) or ASFV-WT (n = 5). (D) Tissue lesions in the spleens, tonsils, submaxillary lymph nodes, and inguinal lymph nodes of pigs infected with ASFV-WT or ASFV-intB318L. **(E)** qPCR analysis of ASFV genomic DNA copy number in blood samples obtained from pigs at 0, 1, 4, 7, 11, 16, and 21 days after being infected with either ASFV-intB318L or ASFV-WT. **(F)** qPCR analysis of ASFV genomic DNA copy number in the tissues as indicated obtained from pigs that were infected with either ASFV-intB318L or parental ASFV-WT. **(G)** Histopathological section of thymus, spleen, submaxillary lymph nodes, inguinal lymph nodes, and bronchial lymph nodes. **(H-K)** qPCR analysis of mRNA levels of *Ifnα*(H), *Ifnb1*(I), *Isg15* (J), and *Mx1* (K) in the spleen, tonsil, thymus, submaxillary lymph node, bronchial lymph node, and gastrohepatic lymph node obtained from pigs mock infected or infected with either of ASFV-intB318L or parental ASFV-WT. **(L-M)** The protein levels of IFN-α and IFN-β in peripheral serum samples from ASFV-intB318L or ASFV-WT-challenged pigs were monitored at 0, 1, 4, and 7 dpi by ELISA. Data represent three independent experiments with three biological replicates (mean ± s.d.). Ns, not significantly, * *p* < 0.05, ** *p* < 0.01, *** *p* < 0.001, (one-way ANOVA).

To examine the role of the *B318L* gene in ASFV pathogenicity, SPF pigs were incubated with 10^2.5^ HAD_50_ ASFV-WT or ASFV-intB318L, respectively. All pigs infected with ASFV-WT developed a fever at 4 days post inoculation (dpi), were depressed, and had a reduced appetite at 5 dpi. ASFV-WT-infected pigs started to die from 7 dpi, and all died within 11 dpi (Fig 10A–10C). In comparison, only two pigs infected with ASFV-intB318L developed a fever at 5 dpi, and then showed significant depression of spirit and loss of appetite at 10 dpi (Fig 10A and 10B). ASFV-intB318L infected pigs started to die from 12 dpi, and 60% of pigs continued to survive until 21 dpi (Fig 10A and 10C). The spleens, tonsil, submandibular lymph nodes, and inguinal lymph nodes from pigs challenged with ASFV-WT showed extensive bleeding, while these tissues from pigs challenged with ASFV-intB318L showed no obvious lesions (Fig 10D).

Pigs in ASFV-WT inoculated group presented viremia at 4 dpi, while only two pigs in ASFV-intB318L-challenged group presented viremia at 10 dpi (Fig 10E). Viral DNA copy numbers in organs/tissues including heart, liver, lung, spleen, kidney, tonsil, and six lymph nodes (mediastinal, mesenteric, inguinal, submandibular, bronchial, and gastrohepatic) detected in ASFV-intB318L-infected pigs were significantly lower than that detected from the ASFV-WT-infected group (Fig 10F). Histopathological sections showed massive necrosis and decrease of lymphocytes in thymus, bronchial lymph nodes, inguinal lymph nodes and submandibular lymph nodes and increased hematocrit in spleen in ASFV-WT infection group. However, these pathological phenomena were significantly milder in the ASFV-intB318L infection group (Fig 10G).

To compare the innate immune responses of pigs inoculated with ASFV-WT or ASFV-intB318L, the mRNA levels of IFN-I and ISGs in the immune tissues as indicated were detected by qPCR. The results showed that the mRNA levels of *Ifnα*, *Ifnb1*, *Mx1*, and *Isg15* in the tissues of pigs challenged with ASFV-intB318L were significantly higher than those in the tissues of pigs challenged with ASFV-WT (Fig 10H–10K). The protein levels of IFN-α and IFN-β in peripheral serum samples were also monitored at 0, 1, 4, and 7 dpi using ELISA. The results showed that the secretions of IFN-α and IFN-β in the ASFV-intB318L-inoculated group were 2.0 and 2.5 folds higher than those in the ASFV-WT-inoculated group at 7 dpi, respectively (Fig 10L and 10M).

### Antiviral immune responses induced by ASFV-intB318L infection are related to the viral virulence

To elucidate the correlation between pB318L-suppressed ISGs production and the virulence of ASFV, we detected and compared the effect of IFN-α on the replications of ASFV-WT and ASFV-intB318L. We found that IFN-α reduced the replication of ASFV-intB318L more significantly than that of ASFV-WT, and treatment with anti-IFNAR1 and anti-IFNAR2 antibodies rescued the inhibitory effect of IFN-α (S6A Fig). In addition, deucravacitinib, a TYK2-specific inhibitor, was used to confirm the effect of IFN-α-induced JAK-STAT signaling on ASFV replication. We found that deucravacitinib strongly reduced IFN-α-induced ISRE promoter activity and *Isg*15 mRNA levels (S6B and S6C Fig), and is non-toxic to cells at the concentrations of 10 nM (S6D Fig), suggesting that deucravacitinib functions well to antagonize the JAK-STAT signaling. As we expected, deucravacitinib also rescued the inhibition of IFN-α on the replications of ASFV-WT and ASFV-intB318L (S6E Fig). These results suggest that ASFV-intB318L is more sensitive to IFN-α than ASFV-WT, and IFN-α reduced the replication of the ASFV through inducing the production of ISGs.

In summary, all these results showed that ASFV-intB318L infection induced more IFN-I and ISGs in the infected pigs compared with its parental ASFV-WT. As a result, the ASFV-intB318L is partially attenuated in pigs because the immunized pigs had less viral load and a higher survival rate. We concluded that B318L gene is not a key virulence-related gene.

## Discussion

The first ASF case was reported in China in 2018, and the ASFV HLJ/18 isolate was subsequently confirmed to be a highly virulent strain with 100% mortality in domestic pigs [27]. Until now, ASF has still caused huge economic losses to the world pig industry due to a lack of safe, effective commercial vaccines and drugs. It has been reported that the ability of ASFV to escape host antiviral immune responses is closely related to its pathogenicity. Therefore, deletion of ASFV virulence-related gene(s) may represent a reasonable strategy to develop live attenuated vaccines. Previous studies showed that ASFV infection inhibits the activation of the JAK-STAT signaling pathway and cGAS-STING signaling pathway [28,29]. In this study, we found that ASFV pB318L reduces the production of ISGs by targeting STING in the cGAS-STING pathway and IFNAR1/IFNAR2 in the IFNAR-JAK-STAT pathway, interruption of the reading frame of *B318L* gene attenuated ASFV pathogenicity. Our findings provide a paradigm to screen and identify key virulence genes for ASFV.

Previous studies showed that IFN-I production is critical for host antiviral immune responses, and ASFV-encoded proteins inhibit the production of IFN-I and the expression of ISGs to evade the host antiviral immune responses, which benefits viral replication [30,31]. For example, ASFV pDP96R reduces TBK1 and IKKβ-induced activation of the NF-κB promoter, thereby reducing IFN-I production [32]. ASFV pE120R interferes with the recruitment of IRF3 to TBK1 and inhibits the activation of IRF3, thereby negatively regulating the production of IFN-I [33]. Recently, we reported that ASFV pI215L reduces IFN-I production by recruiting the E3 ubiquitin ligase RNF138 to degrade RNF128 and inhibits RNF128-mediated K63-linked polyubiquitination of TBK1 [22]. cGAS-STING signaling plays an important role in inducing the production of IFN-I during DNA virus infection. Several viruses were reported to reduce IFN-I production through targeting STING. For example, murine cytomegalovirus (MCMV) m152 inhibits IRF3 activation and negatively regulates IFN-I production through delaying STING translocation [34]. Herpes simplex virus type 1 (HSV-1) VP11/12 interacts with STING to promote the degradation of STING, thereby reducing the production of IFN-I [35]. HCMV UL48 inhibits STING ubiquitination, thereby inhibiting STING activation and IFN-I production [36]. Previous studies also showed that ASFV pMGF505-7R promotes ULK1-mediated STING degradation, which inhibits the phosphorylation and nuclear transport of IRF3, thereby reducing IFN-I production [37]. In the rest state, STING is located on ER through its transmembrane region, and the translocation of STING from ER to Golgi apparatus is necessary for its activation in cGAS-STING signaling. In this study, we found that pB318L interacts with the transmembrane region of STING. STING is localized on organelles through its transmembrane region, and the translocation of STING from ER to Golgi apparatus is necessary for its activation in cGAS-STING signaling [8]. Furthermore, we demonstrated that pB318L reduces the translocation of STING to the Golgi apparatus, thereby reducing the activation of downstream TBK1 and IRF3, resulting in the inhibition of IFN-I production (Figs 4 and S3). Taken together, like other viruses, ASFV also targets cGAS-STING-TBK1-IRF3 axis to inhibit IFN-I production, resulting in escaping host innate immune responses.

IFNs play potent antiviral roles by inducing ISGs production through activating IFNAR-JAK-STAT signaling. Previous studies showed that a variety of viruses develop multiple strategies to inhibit the IFNAR-JAK-STAT signaling pathway. For example, Zika virus (ZIKV) NS5 promotes the degradation of STAT2 through the proteasome pathway, thereby inhibiting the formation of ISGF3 and reducing the production of ISGs [38]. Rotavirus NSP1 mediates IRF9 degradation and inhibits IFN-mediated STAT1 phosphorylation [39]. Recently, ASFV pMGF360-9L was found to promote STAT1 and STAT2 degradation via apoptosis and ubiquitin-proteasome pathways, respectively, thereby inhibiting the production of ISGs [40]. In this study, we clearly demonstrated that ASFV pB318L interacts with IFNAR1 and IFNAR2 to inhibit the interaction of IFNAR1-TYK2 and IFNAR2-JAK1, leading to reduced production of ISGs induced by IFN-α (Fig 8). These data indicated that more than one of ASFV proteins inhibit antiviral ISGs production by targeting the IFNAR-JAK-STAT signaling.

It has been reported that most of the small GTPase family members, including Rab, Ras, and Rho, can be modified by prenylation, which is involved in viral infection [12, 41]. Incorporation of prenyl groups into Rho GTPases plays a crucial role in several stages of ASFV infection since both geranylgeranyl and farnesyl pyrophosphates are required for viral replication. Interestingly, inhibition of Rho GTPase impairs virus morphogenesis and results in an abnormal viral factory size with an accumulation of envelope precursors and immature virions, suggesting that Rho GTPase is involved in ASFV infection, invasion, and virion assembly [42]. Rac1, a member of Rho-family small GTPases, is activated at the early stage of ASFV infection, and inhibition of Rac1 does not affect the viral entry but impairs subsequent virus production by inhibiting microtubule acetylation and viral intracellular transport [42]. Rab1B, another member of the Rab family, functions in the early secretory pathway and is essential for vesicle transport between the ER and Golgi [43–45]. Monogeranylgeranylated Rab1B can reversibly bind to a negatively charged model membrane. Recently, Rab1B was found to promote antiviral innate immunity by interacting with TNF receptor-associated factor 3 (TRAF3) [46]. ASFV pB318L is homologous to GGPPS. In this study, we demonstrated that ASFV pB318L negatively regulates IFN-I and ISGs expression, which depends on its enzymatic activity (Figs 5 and 9). Moreover, we demonstrated that Asp129 of pB318L is crucial for its function, and this site was also identified as a key site of pB318L in a previous article [19]. However, the specific mechanism through which the GGPPS activity of B318L affects the production of IFN-I and ISGs is not known. Previous studies have shown that pB318L plays a role in the formation of virus morphology [20]. As a GGPPS, pB318L may play an important role in other biological processes to regulate the interaction between ASFV and the host. The inhibitory effects of IFN and ISGs studied in this article may not be the main factor affecting the pathogenicity of ASFV, which prompts us to further investigate in the future.

Accumulating evidence showed that deletion of ASFV genes that are involved in inhibiting the production of IFN-I and ISGs results in a decrease in viral virulence [37,47,48]. Recently, we reported that pMGF505-7R reduces type I IFN production and IL-1β release. Consequently, the virulence of ASFV-ΔMGF505-7R is reduced in piglets compared with its parental ASFV HLJ/18 strain, which may be due to induction of higher type I IFN production [49]. In this study, pB318L was found to be a negative regulator of IFN-I and ISGs production. To study the effect of *B318L* gene during ASFV infection, a recombinant virus that interrupted the reading frame of *B318L* gene (ASFV-intB318L) was generated. Unexpectedly, the single nucleotide insertion near the terminus of the *D1133L* gene occurred in ASFV-intB318L except for interrupted *B318L*. However, the single nucleotide insertion in the D1133L gene resulted in a gene sequence that is entirely identical to the *D1133L* gene of the Georgia 2007/1 strain that belongs to genotype II with the HLJ/18 strain. Therefore, the mutation of *D1133L* neither affects its original function nor the expression of the downstream *D339L* gene. Pigs infected with ASFV-intB318L had a 60% survival rate, while all pigs infected with ASFV-WT died within 11 days (Fig 10A), suggesting that the virulence of ASFV-intB318L is significantly lower than its parental virus. Although the mortality rate of ASFV-intB318L is still 40%, it is worth noting that the pigs in ASFV-intB318L infection group had significantly lower viral load than that in the ASFV-WT infection group, and higher levels of IFN-I and ISGs (Fig 9E, 9F and 9H–9M), suggesting that combined deletion of *B318L* gene and other virulence-related gene(s) may be a viable strategy for developing live attenuated vaccines.

In conclusion, we presented evidence that ASFV pB318L reduces the production of IFN-I through interacting with STING and thus reducing its translocation from the endoplasmic reticulum to the Golgi apparatus. Additionally, pB318L binds to IFNAR1/IFNAR2 and impairs the interactions between IFNAR1 and TYK2 and IFNAR2 and JAK1, thereby negatively regulating IFNAR-JAK-STAT signaling (S7 Fig). Interestingly, the inhibition of IFN-I and ISGs production by pB318L is related to its GGPPS enzymatic activity. Interruption of the reading frame of *B318L* gene partially attenuates the pathogenicity of ASFV HLJ/18 due to the induction of higher levels of IFN-I and ISGs. Overall, our results reveal that *B318L* gene is not a key virulence-related gene, which can be used as a candidate gene for the development of live attenuated vaccines with other genes.

## Materials and methods

### Ethics statements

All experiments with ASFV HLJ/18 (Genbank accession number: MK333180.1) and ASFV-intB318L were conducted within the enhanced biosafety level 3 (P3+) and level 4 (P4) facilities in the Harbin Veterinary Research Institute (HVRI) of the Chinese Academy of Agricultural Sciences (CAAS) approved by the Ministry of Agriculture and Rural Affairs. This study was performed in accordance with the Guide for the Care and Use of Laboratory Animals of the Ministry of Science and Technology of the People’s Republic of China. The protocols were approved by the Committee on the Ethics of Animal Experiments of the HVRI of CAAS (Approval numbers: 220113-03-GJ).

### Reagents and antibodies

Dulbecco’s Modified Eagle’s Medium (DMEM) (C11995500CP), RPMI 1640 (C11875500CP), and fetal bovine serum (FBS) (10091–148) were purchased from GIBCO (Grand Island, NE, USA). cGAMP (SML1232), poly(dA:dT) (P0883-10UN), GGTI-286 (345878-250UG), and anti-Flag (M2) beads (M8823) were purchased from Sigma-Aldrich (St. Louis, MO, USA). Lovastatin (S2061) and Lonafarnib (S2797) were purchased from Selleckchem (Houston, TX, USA). Deucravacitinib (M4138) was purchased from Abmole (Houston, TX, USA). Protease inhibitor Cocktail (4693132001) was purchased from Roche (Basel, Switzerland). Dual-Luciferase Reporter Assay System (E1910) was purchased from Promega (Madison, MI, USA). PrimeScript RT Reagent Kit (RR037A) and SYBR Premix Ex Taq II (RR820A) were purchased from Takara (Shiga, Japan). The following antibodies were purchased from Sigma-Aldrich (St. Louis, MO, USA): rabbit anti-Flag (F7425-2MG), mouse anti-Flag (F1804-1MG), rabbit anti-HA (SAB4300603), mouse anti-HA (HS658-2ML), mouse anti-AP-1 (A4200). The following antibodies were purchased from Proteintech (Wuhan, China): Mouse anti-GAPDH (60004-1-Ig), rabbit anti-Lamin B (12987-1-AP), rabbit anti-TBK1 (28397-1-AP), rabbit anti-IFNAR1 (13083-1-AP) and rabbit anti-IFNAR2 (10522-1-AP). The following antibodies were purchased from Cell Signaling Technology (Danvers, MA, USA): rabbit anti-STING (13647), rabbit anti-Phospho-STING (19781), rabbit anti-Phospho-TBK1 (5483), rabbit anti-IRF3 (11904), rabbit anti-Phospho-IRF3 (29043), rabbit anti-STAT1 (9172S), rabbit anti-Phospho-STAT1 (9167S), rabbit anti-STAT2 (72604) and rabbit anti-Phospho-STAT2 (88410). pGEX-6p-1-B318L was constructed and then transformed into BL21 (DE3) to express recombinant pB318L protein. BALB/c mice were immunized with GST affinity chromatography column purified pB318L protein to generate anti-pB318L antibody. Anti-p30, anti-p54, and anti-pig STING antibodies are all produced and stored in our laboratory. The IRDye 800CW goat anti-rabbit IgG (H+L) (925–32211) and IRDye 800CW goat anti-mouse IgG (H+L) (925–32210) were purchased from LI-COR (Lincoln, NE, USA). Alexa Flour 488 goat anti-Rabbit IgG(H+L) (A11008) and Alexa Flour 594 goat anti-Mouse IgG(H+L) (A11032) were purchased from Thermo Fisher Scientific (Waltham, MA, USA). Porcine IFN-β ELISA Kit (orb549069) was purchased from Biorbyt (Cambridge, UK). Porcine IFN-α ELISA Kit (ELP-IFNα) was purchased from RayBio (Norcross, GA, USA). Minute Golgi Apparatus Enrichment Kit (GO-037) was purchased from Invent Biotechnologies, Inc. (Eden Prairie, MN, USA). Nuclear and Cytoplasmic Extraction Reagents (VL315819) were purchased from Thermo Fisher Scientific (Waltham, MA, USA)

### Plasmids

The IFN-α, IFN-β, NF-κB, ISRE, ISG54, ISG56 reporters, and TK-Renilla reporter were obtained from Professor Hong Tang. To construct plasmids expressing Flag-tagged or HA-tagged proteins in the cGAS-STING signaling and IFN signaling, the cDNAs corresponding to these swine genes were amplified by RT-PCR using total RNA extracted from PAMs as templates and were then cloned into the pCAGGS-Flag or pCAGGS-HA vector, respectively. To express EGFP-tagged pB318L, Flag-tagged pB318L, and HA-tagged STING, the cDNAs of these genes were amplified and cloned into the pEGFP, pCAGGS-Flag vector or pCAGGS-HA vector, respectively. To express Flag-tagged pB318L mutants, we designed primers for the nucleotide sequences, including Asp129, Asp135, and Asp212. By using these primers, we mutated the base that was originally translated as Asp to the base translated as Ala. The mutated fragments were amplified and cloned into the pCAGGS-Flag vector. All constructs were validated by DNA sequencing. The primers used in this study are listed in the S2 Table.

### Cell lines and viruses

HEK293T cells and CRL-2843 cells were purchased from American type culture collection (ATCC, Rockville, MD, USA) and cultured in DMEM. Primary pulmonary alveolar macrophages (PAMs) isolated from specific pathogen-free (SPF) piglets (without ASFV, PRRSV, PRV, PCV2, and other 28 pathogens) were cultured in RPMI 1640 supplemented with 10% FBS, 100 U/mL penicillin, and 100 μg/mL streptomycin at 37°C with 5% CO_2_. ASFV HLJ/18 (GenBank accession number: MK333180.1) strain was isolated from a pig sample from an ASF outbreak farm in China [27].

### Generation of B318L gene-interrupted ASFV (ASFV-intB318L)

A recombinant ASFV was generated by homologous recombination in PAMs. Plasmid pBluscript II KS (+) was used as a backbone, a cassette containing the overlap sequence of *B318L* and *B438L*, two LoxP sequences and the fluorescent gene *EGFP* under the control of the ASFV p72 promoter was inserted into the upstream of the *B318L* gene, and the first, and tenth nucleotide “A,” “C” of *B318L* ORF was deleted. Recombinant transfer vector (pB-intB318L-eGFP) containing about 800 bp left homologous arm at the left of 96238 site of ASFV/HLJ/18 genome, a reporter gene cassette, followed by about 800 bp right homologous arm at the right of 96239 site of ASFV/HLJ/18 genome. The recombinant ASFV was constructed and purified as described before [49]. The DNA sequence covering the modified region was amplified and sequenced (S1 Table). The whole genome sequence of ASFV-intB318L was sequenced and aligned with that of ASV-HLJ/18 (S1 Appendix and S2 Appendix). The primers used to construct recombinant viruses are listed in the S2 Table.

### ASFV complete genome sequencing and analysis

PAMs were infected with ASFV-WT or ASFV-intB318L, and viral DNA was extracted from the cell supernatant. Whole genome sequencing was performed on ASFV. Viral DNA was broken into segments by ultrasonic crusher. In addition, DNA libraries were prepared through end repair, addition of sequencing connectors, purification, and PCR amplification. Then, the insertion fragment size of the library was detected using Agilent 2100. Accurately quantify the effective concentration of the library by qPCR to ensure library quality. Sequencing of DNA library using Illumina HiSeq. The mutation of *B318L* in ASFV-intB318L was confirmed through whole genome sequencing (S1 Table and S2–S3 Appendix).

### Virus cultivation and purification

PAMs (Approximately 1× 10^7^ cells) were infected with ASFV (MOI = 1) and amplified at 37°C. The supernatant was collected at 3 days post-infection and centrifuged at 5000×g for 15 minutes to remove cells and other debris. Then, the supernatant was further purified through a 30% (w/v) sucrose buffer to centrifuge at 15000×g for 3 hours. Virus-containing particles were resuspended in PBS and loaded onto a sucrose density gradient of 25%-56% (w/v) to be centrifuged at 80000×g for 1.5 hours. Virus band was extracted from gradients and diluted with PBS, and centrifuged at 15000×g for 1.5 hours to precipitate virus particles. Virus particles were resuspended in 10 mL RPMI 1640 and stored at -80°C.

### Co-immunoprecipitation (Co-IP) and immunoblot analysis

Co-IP and immunoblot analysis were performed, as mentioned previously [50]. In brief, for Co-IP, the cells were collected and lysed in lysis buffer (50 mM Tris-HCl, pH 7.4, 150 mM NaCl, 5 mM MgCl_2_, 1 mM EDTA, 1% Tris-HCl, and 10% glycerol) containing 1 mM PMSF and 1 × protease inhibitor cocktail (Basel, Switzerland, Roche). Then, cell lysates were incubated with anti-flag (M2) beads (Sigma-Aldrich, St. Louis, MO, USA) or added indicated antibodies and protein A+G Plus-Agarose (Santa Cruz Biotechnology, Dallas, TX, USA). After incubation 4–8 h at 4°C, the beads were washed three times with lysis buffer. For immunoblot analysis, the samples were separated by 10–12% sodium sulfate polyacrylamide gel electrophoresis (SDS-PAGE) and then transferred to a polyvinyl difluoride (PVDF) membrane (Sigma-Aldrich, St. Louis, MO, USA). After incubation with primary and secondary antibodies, the membrane is visualized by the Odyssey Dual Color Infrared Fluorescence Imaging System (LI-COR, Lincoln, NE, USA).

### Confocal microscopy and co-localization analysis

The cells were transfected with indicated plasmids, and the cell supernatant was discarded at 24 hpt. The cells were fixed for 10 min in 4% paraformaldehyde and then permeabilized for 15 min with 0.3% Triton X-100. After blocking in 1 × PBS with 10% FBS for 30 min, the cells were incubated with the appropriate primary antibodies and then stained with secondary antibodies as indicated. The subcellular co-localization was visualized using a Zeiss LSM-880 laser scanning fluorescence microscope (Carl Zeiss AG, Oberkochen, Germany) under a 63 × oil objective. Zeiss processing system software was used to determine the degree of pB318L and STING or IFNAR1/IFNAR2 co-localization. Calculate the Pearson correlation coefficient of the two colors in the region by selecting the area where two protein staining overlaps.

### Luciferase reporter gene assay

HEK293T cells were co-transfected with the indicated plasmids. After 24 h, the cells were lysed in lysis buffer, and luciferase activities of IFN-β-, NF-κB-, ISG56-, ISG54, ISRE-luciferase-reporter (50 ng), and TK-Renilla reporter (50 ng) were measured with a Dual-Luciferase Reporter Assay System (Promega, Madison, MI, USA) according to the manufacturer’s instructions. The data were normalized to the transfection efficiency by dividing the firefly luciferase activity by the Renilla luciferase activity. Each experiment was conducted three times independently, and the representative results were shown.

### RNA extraction and qPCR

Total RNA was extracted using TRIzol reagent (Thermo Fisher Scientific, Waltham, MA, USA), and reverse transcription was accomplished with the PrimeScript RT Reagent Kit (Takara, Shiga, Japan). The reverse transcription products were amplified using the Agilent-Strata gene Mx Real-Time qPCR system with SYBR Premix Ex Taq II (Takara, Shiga, Japan) according to the manufacturer’s instructions. The data were normalized according to the level of β-actin expression in each individual sample. All experiments were performed at least in triplicate. For ASFV genome DNA copy detection, ASFV genomic DNA was extracted using DNA Mini Kit (Qiagen, Dusseldorf, Germany) from cells, tissue homogenates, or EDTA-treated whole peripheral blood. ASFV genomic DNA and standard sample were amplified using the Agilent-Strata gene Mx Real-Time qPCR system with Premix Ex Taq (Takara, Shiga, Japan) according to the manufacturer’s instructions, and ASFV genome DNA copy numbers were calculated from the standard curve based on the Ct value. All the qPCR primers are listed in S3 Table.

### GST pull-down assay

The ASFV pB318L transmembrane region-removed sequence was cloned into the pGEX-6p-1 vector and transformed into BL21 competent cells. The obtained bacteria were expanded, cultured, and induced with 0.5 mM IPTG for 20 h. After centrifugation, the cells were resuspended in PBS and then sonicated to obtain bacterial lysates containing GST-pB318L. HA-tagged STING was transfected into HEK293T cells, and cells were lysed in lysis buffer (50 mM Tris-HCl, pH 7.4, 150 mM NaCl, 5 mM MgCl_2_, 1 mM EDTA, 1% Tris-HCl and 10% glycerol) containing 1 mM PMSF and 1 × protease inhibitor cocktail (Basel, Switzerland, Roche) at 24 hpt. The cell lysates were mixed with bacterial lysates containing GST-pB318L and added to GST beads (GenScript, NJ, USA) to incubate for 6–8 h at 4°C. The beads were washed five times with lysis buffer and then subjected to Western blotting analysis.

### ELISA

The concentrations of IFN-α (Ray Biotech, Norcross, GA) and IFN-β (Biorbyt, Cambridge, UK) in the cell culture supernatants and sera were measured by ELISA kits according to the manufacturer’s instructions.

### Nuclear and cytoplasmic extraction

HEK293T cells were transfected with the indicated plasmids for 24 h and then stimulated with IFN-α for another 12 h. After that, the cells were harvested and processed using Nuclear and Cytoplasmic Extraction Reagent (Thermo Scientific, Waltham, MA, USA). PAMs were infected with ASFV-WT or ASFV-intB318L for 24 h (MOI = 1) and processed using Nuclear and Cytoplasmic Extraction Reagent. Nuclear and cytoplasmic extracts were examined by Western blotting.

### Golgi Apparatus Enrichment assay

HEK293T cells were transfected with the indicated plasmids for 24 h and then treated with STING agonist for another 6 h. After that, the cells were harvested, and Golgi Apparatus was isolated with an enrichment reagent (Invent Biotechnologies, Eden Prairie, MN, USA) according to the instructions. PAMs were infected with ASFV-WT or ASFV-intB318L for 24 h (MOI = 1), and isolated Golgi Apparatus with Enrichment reagent. Golgi extracts were examined by Western blotting.

### The virulence of ASFV-intB318L in domestic pigs

Animal experiments were performed within the animal biosafety level 4 facilities at HVRI following a protocol approved by the Animal Ethics Committee of HVRI of CAAS and the Animal Ethics Committee of Heilongjiang Province, China (Approval numbers: 220113-03-GJ). Thirteen 8-week-old healthy SPF piglets were randomly assigned into three groups (5 piglets infected with ASFV-intB318L, 10^2.5^ HAD_50_/piglet; 5 piglets infected with ASFV-WT, 10^2.5^ HAD_50_/piglet; 3 piglets inoculated with PBS). The piglets were monitored daily for clinical signs prior to feeding, including anorexia, lethargy, fever, and emaciation. The blood samples were collected at 0, 1, 4, 7, 11, 16, and 21 days post-infection (dpi) for virus load detection, and the serum samples were collected at days 0, 1, 4, and 7 dpi to detect IFN-β and IFN-α using ELISA kits. ASFV- infected piglets were euthanized in the moribund stage. At 21 dpi, all surviving piglets were euthanized. The tissue samples from the heart, liver, spleen, lung, kidney, tonsil, thymus, and six lymph nodes (inguinal lymph node, submaxillary lymph node, bronchial lymph node, gastrohepatic lymph node and mesenteric lymph nodes, mediastinal lymph nodes) were collected for ASFV detection.

### Statistical analysis

Statistical analysis was conducted using the unpaired Student’s t-test and one-way analysis of variance (ANOVA) followed by the Bonferroni post-test. P values less than 0.05 were considered statistically significant. Sample sizes were chosen by standard methods to ensure adequate power, and no exclusion, randomization of weight, sex, or blinding was used for the animal studies.

## Supporting information

S1 Fig Generation of recombinant ASFV-intB318L virus. **(A)** Schematic representation of the generation of ASFV-intB318L virus. **(B)** PCR verification of the insertion of the p72-EGFP reporter gene cassette in the genome of ASFV-intB318L virus. PAMs infected with ASFV-WT or ASFV-intB318L, at 24 hpi, the virus DNA was extracted and amplified with primers (sequences in ASFV *B119L* and ASFV *B438L* respectively). **(C)** PAMs were infected with ASFV-WT or ASFV-intB318L (MOI = 1). At 24 hpi, cells were lysed and the expression of pB318L was detected by Western blotting. **(D)** PAMs were infected with ASFV-WT or ASFV-intB318L. At 24 hpi, the cells were observed by microscope for GFP expression. **(E)** Growth kinetics of ASFV-WT and ASFV-intB318L in PAMs. PAMs were infected with ASFV-intB318L or ASFV-WT, and the TCID_50_ was monitored at 24, 48, 72, and 96 hpi, respectively.
(TIF)

S2 Fig The effect of I215L and H171R on IFN-β-Luc activity induced by cGAS-STING. (**A)** HEK293T cells were transfected with an IFN-β-Luc reporter, a Renilla-TK reporter, and plasmids expressing HA-cGAS and HA-STING, together with increasing amounts (100 ng, 200 ng, 400 ng) of a plasmid expressing Flag-I215L or Flag-H117R. Luciferase activities were analyzed at 24 hpt. Expressions of the proteins were analyzed by Western Blotting. Data are representative of three independent experiments with three biological replicates (mean ± s.d.). Ns, not significantly, *** *p* < 0.001 (one-way ANOVA).
(TIF)

S3 Fig ASFV pB318L interacts with STING and inhibits the location of STING on Golgi. **(A)** HEK293T cells were transfected with plasmids expressing Flag-pB318L and HA-STING. Co-IP analysis was performed using Flag-beads to detect the interaction between pB318L and STING after 24 h. **(B)** HEK293T cells were transfected with plasmids expressing Flag-pB318L and HA-STING. Co-IP analysis was performed using HA-beads to detect the interaction between pB318L and STING after 24 h. **(C)** Direct interaction between STING and ASFV pB318L was detected by GST pull-down assay. **(D)** Schematic diagram of full-length pB318L and its truncated mutants. **(E)** HEK293T cells were transfected with a plasmid expressing Flag-STING (2 μg), along with plasmids expressing HA-pB318L-WT, HA-pB318L-D1, HA-pB318L-D2, HA-pB318L-D3, HA-pB318L-D4, HA-pB318L-D5, HA-pB318L-D6 (2 μg/each), respectively. The cells were collected at 24 hpt, and the interactions of pB318L and its deleted mutants with STING were analyzed by Co-IP and Western blotting. The asterisk represents non-specific bands. **(F-G)** HeLa cells were transfected with HA-pB318L or HA-vector for 24 h, and stimulated with STING agonist for an extra 6 h. Then, the cells were harvested and treated with Golgi Apparatus Enrichment reagent to detect STING on Golgi **(F)**. The quantitation ratio of STING on Golgi was analyzed with image J **(G)**. **(H)** HEK293T cells were transfected with plasmids expressing Flag-Rho A and HA-pB318L or HA-pB318L-D129A. Co-IP analysis was performed to detect prenylation of Rho A after 24 h. Data are representative of three independent experiments with three biological replicates (mean ± s.d.). *** *p* < 0.001 (one-way ANOVA).
(TIF)

S4 Fig IFN-α treatment does not affect the mRNA level of p72 of ASFV and ASFV-intB318L. PAMs were infected with ASFV-WT or ASFV-intB318L (MOI = 1) for 24 h, and then treated with IFN-α (1 μg/mL) for another 12 h. The mRNA levels of ASFV p72 were analyzed by qPCR. Data represent three independent experiments with three biological replicates (mean ± s.d.). * *p* < 0.05 (one-way ANOVA).
(TIF)

S5 Fig ASFV pB318L interacts with IFNAR1 and IFNAR2. **(A)** HEK293T cells were transfected with plasmids expressing HA-IFNAR1 (up) or HA-IFNAR2 (down) and Flag-pB318L. At 24 hpt, the cells were lysed and combined with Flag-beads, and the interaction between pB318L and IFNAR1 was detected by Co-IP. **(B)** HEK293T cells were transfected with plasmids expressing HA-IFNAR1 (up) or HA-IFNAR2 (down) and Flag-pB318L. At 24 hpt, the cells were lysed and combined with HA beads, and the interaction between pB318L and IFNAR1 was detected by Co-IP. **(C-D)** PAMs were mock infected or infected with ASFV or ASFV-intB318L. At 24 hpt, cells are lysed and incubated with antibodies against IFNAR1 (C) or IFNAR2 (D), and the interaction between pB318L and endogenous IFNAR1/IFNAR2 was detected by Co-IP. **(E-F)** HEK293T cells were transfected with plasmids expressing HA-IFNAR1 (E) or HA-IFNAR2 (F), at 24 hpt, the cells are lysed and incubated with GST or GST-pB318L. Direct interaction of IFNAR1 or IFNAR2 with ASFV pB318L was detected by GST pull-down assay. **(G-H)** HEK293T cells were transfected with a plasmid expressing Flag-IFNAR1 (G) or IFNAR2 (H) (2 μg), along with plasmids expressing HA-pB318L-WT, HA-pB318L-D1, HA-pB318L-D2, HA-pB318L-D3, HA-pB318L-D4, HA-pB318L-D5, HA-pB318L-D6 (2 μg), respectively. The cells were collected at 24 hpt and the interactions of pB318L and its deleted mutants with IFNAR1 or IFNAR2 were analyzed by Co-IP and Western blotting.
(TIF)

S6 Fig The *B318L* gene antagonizes the inhibitory effect of IFN-α on ASFV replication. **(A)** PAMs were pre-incubated with anti-IFNAR1 and anti-IFNAR2 antibodies for 2 h, followed by treatment with IFN-α (1 μg/mL) for 12 h, and then infected with ASFV-WT or ASFV-intB318L. At 24 hpi, qPCR was performed to analyze the ASFV genomic DNA copy numbers in the cells. **(B)** HEK293T cells were transfected with an ISRE-Luc reporter and a Renilla-TK reporter. At 24 hpt, the cells were treated with different doses of deucravacitinib (2.5 nM, 5 nM, and 10 nM) for 6 h, and then treated with IFN-α (1 μg/mL) for another 12 h. After that, the luciferase activities were analyzed. **(C)** PAMs were treated with different doses of deucravacitinib (2.5 nM, 5 nM, and 10 nM) for 6 h, and then treated with IFN-α (1 μg/mL) for another 12 h. The mRNA levels of *Isg15* were analyzed by qPCR. **(D)** PAMs were treated with different doses of deucravacitinib (2.5 nM, 5 nM, and 10 nM) for 24 h; LDH assay was then performed according to the manufacturer’s instructions. **(E)** PAMs were pretreated with deucravacitinib (10 nM) for 6 h, followed by treatment with IFN-α (1 μg/mL) for 12 h, and then infected with ASFV-WT or ASFV-intB318L. At 24 hpi, qPCR was performed to detect ASFV genomic DNA copy numbers in the cells. Data are representative of three independent experiments with three biological replicates (mean ± s.d.). Ns, not significantly, * *p* < 0.05, ** *p* < 0.01, *** *p* < 0.001 (one-way ANOVA).
(TIF)

S7 Fig Schematic representation of the negative regulation of ASFV pB318L in the cGAS-STING and JAK-STAT signaling pathways. Following ASFV infection, host cGAS senses ASFV genomic DNA, which promotes cGAMP production. cGAMP binds to STING to promote STING activation and translocation from the ER to the Golgi apparatus. Active TBK1 phosphorylates IRF3, promotes the translocation of IRF3 to the nucleus, and initiates the transcription of IFN-I. Secreted IFN-I binds to IFNAR1 and IFNAR2, resulting in activation of JAK1 and TYK2 kinases. The phosphorylated STAT1/2 and IRF9 form a heterotrimeric complex ISGF3. Subsequently, ISGF3 enters the nucleus to bind ISRE to regulate the transcription of ISGs. ASFV pB318L interacts with STING and inhibits the transfer of STING from the endoplasmic reticulum to the Golgi apparatus, thereby inhibiting IFN-I production. On the other side, pB318L binds to IFNAR1/IFNAR2 and blocks the phosphorylation of TYK2 and JAK1, thereby negatively inhibiting IFN-mediated ISGs production.
(TIF)

S1 Table Primers used for plasmid construction in this study. (DOCX)

S2 Table The DNA sequence covering the modified region of ASFV. (DOCX)

S3 Table Primers used for qPCR in this study. (DOCX)

S1 Appendix Whole genome sequencing of ASFV-intB318L. (DNA)

S2 Appendix Whole genome sequence alignment. (HTM)

S3 Appendix Mutation position sequence alignment. (DOCX)

## References

[1] Costard S, Wieland B, de Glanville W, Jori F, Rowlands R, Vosloo W, et al. African swine fever: how can global spread be prevented? Philos Trans R Soc Lond B Biol Sci. 2009;364(1530):2683–96. doi: 10.1098/rstb.2009.0098 . 19687038 PMC2865084

[2] Eustace Montgomery R. On A Form of Swine Fever Occurring in British East Africa (Kenya Colony),. J Comp Pathol. 1921;34(0368–1742,):159–91.

[3] Dixon LK, Chapman DA, Netherton CL, Upton C. African swine fever virus replication and genomics. Virus Res. 2013;173(1):3–14. Epub 2012/11/13. doi: 10.1016/j.virusres.2012.10.020 . 23142553

[4] Zheng X, Nie S, Feng WH. Regulation of antiviral immune response by African swine fever virus (ASFV). Virol Sin. 2022;37(2):157–67. Epub 2022/03/13. doi: 10.1016/j.virs.2022.03.006 ; PubMed Central PMCID: PMC9170969. 35278697 PMC9170969

[5] Palm NW, Medzhitov R. Pattern recognition receptors and control of adaptive immunity. Immunol Rev. 2009;227(1):221–33. Epub 2009/01/06. doi: 10.1111/j.1600-065X.2008.00731.x . 19120487

[6] Takeuchi O, Akira S. Pattern recognition receptors and inflammation. Cell. 2010;140(6):805–20. Epub 2010/03/23. doi: 10.1016/j.cell.2010.01.022 . 20303872

[7] Wu J, Sun L, Chen X, Du F, Shi H, Chen C, et al. Cyclic GMP-AMP is an endogenous second messenger in innate immune signaling by cytosolic DNA. Science. 2013;339(6121):826–30. Epub 2012/12/22. doi: 10.1126/science.1229963 ; PubMed Central PMCID: PMC3855410. 23258412 PMC3855410

[8] Ishikawa H, Barber GN. The STING pathway and regulation of innate immune signaling in response to DNA pathogens. Cell Mol Life Sci. 2011;68(7):1157–65. Epub 2010/12/17. doi: 10.1007/s00018-010-0605-2 ; PubMed Central PMCID: PMC3056141. 21161320 PMC3056141

[9] Yum S, Li M, Fang Y, Chen ZJ. TBK1 recruitment to STING activates both IRF3 and NF-κB that mediate immune defense against tumors and viral infections. Proc Natl Acad Sci USA. 2021;118(14):e2100225118. Epub 2021/04/01. doi: 10.1073/pnas.2100225118 . 33785602 PMC8040795

[10] Katze MG, He Y, Gale M. Viruses and interferon: a fight for supremacy. Nat Rev Immunol. 2002;2(9):675–87. doi: 10.1038/nri888 12209136

[11] Sadler AJ, Williams BRG. Interferon-inducible antiviral effectors. Nat Rev Immunol. 2008;8(7):559–68. doi: 10.1038/nri2314 18575461 PMC2522268

[12] Casey PJ. Biochemistry of protein prenylation. J Lipid Res. 1992;33(12):1731–40. Epub 1992/12/01. . 1479283

[13] Goldstein JL, Brown MS. Regulation of the mevalonate pathway. Nature. 1990;343(6257):425–30. Epub 1990/02/01. doi: 10.1038/343425a0 . 1967820

[14] Benetka W, Koranda M, Eisenhaber F. Protein Prenylation: An (Almost) Comprehensive Overview on Discovery History, Enzymology, and Significance in Physiology and Disease. Monatsh Chem. 2006;137(10):1241. doi: 10.1007/s00706-006-0534-9

[15] Maurer-Stroh S, Eisenhaber F. Refinement and prediction of protein prenylation motifs. Genome Biol. 2005;6(6):R55. doi: 10.1186/gb-2005-6-6-r55 15960807 PMC1175975

[16] Palsuledesai CC, Distefano MD. Protein prenylation: enzymes, therapeutics, and biotechnology applications. ACS Chem Biol. 2015;10(1):51–62. Epub 2014/11/18. doi: 10.1021/cb500791f ; PubMed Central PMCID: PMC4301080. 25402849 PMC4301080

[17] Yáñez RJ, Rodríguez JM, Nogal ML, Yuste L, Enríquez C, Rodriguez JF, et al. Analysis of the complete nucleotide sequence of African swine fever virus. Virology. 1995;208(1):249–78. Epub 1995/04/01. doi: 10.1006/viro.1995.1149 . 11831707

[18] Alejo A, Yáñez RJ, Rodríguez JM, Viñuela E, Salas ML. African swine fever virus trans-prenyltransferase. J Biol Chem. 1997;272(14):9417–23. Epub 1997/04/04. doi: 10.1074/jbc.272.14.9417 . 9083080

[19] Zhao H, Zhang H, She Z, Gao Z, Wang Q, Geng Z, et al. Exploring AlphaFold2’s Performance on Predicting Amino Acid Side-Chain Conformations and Its Utility in Crystal Structure Determination of B318L Protein. Int J Mol Sci. 2023;24(3). Epub 2023/02/12. doi: 10.3390/ijms24032740 ; PubMed Central PMCID: PMC9916901. 36769074 PMC9916901

[20] Alejo A, Andrés G, Viñuela E, Salas ML. The African swine fever virus prenyltransferase is an integral membrane trans-geranylgeranyl-diphosphate synthase. J Biol Chem. 1999;274(25):18033–9. Epub 1999/06/11. doi: 10.1074/jbc.274.25.18033 . 10364254

[21] Yang S, Harding AT, Sweeney C, Miao D, Swan G, Zhou C, et al. Control of antiviral innate immune response by protein geranylgeranylation. Sci Adv. 2019;5(5):eaav7999. Epub 2019/06/01. doi: 10.1126/sciadv.aav7999 ; PubMed Central PMCID: PMC6541464. 31149635 PMC6541464

[22] Huang L, Xu W, Liu H, Xue M, Liu X, Zhang K, et al. African Swine Fever Virus pI215L Negatively Regulates cGAS-STING Signaling Pathway through Recruiting RNF138 to Inhibit K63-Linked Ubiquitination of TBK1. J Immunol. 2021:ji2100320. doi: 10.4049/jimmunol.2100320 PubMed Central PMCID: PMC. 34759016

[23] Lin R, Mamane Y, Hiscott J. Structural and Functional Analysis of Interferon Regulatory Factor 3: Localization of the Transactivation and Autoinhibitory Domains. Mol Cell Biol. 1999;19(4):2465–74. doi: 10.1128/MCB.19.4.2465 10082512 PMC84039

[24] Zhang C, Shang G, Gui X, Zhang X, Bai XC, Chen ZJ. Structural basis of STING binding with and phosphorylation by TBK1. Nature. 2019;567(7748):394–8. Epub 2019/03/08. doi: 10.1038/s41586-019-1000-2 . 30842653 PMC6862768

[25] Wang C, Chen Q, Fan D, Li J, Wang G, Zhang P. Structural Analyses of Short-Chain Prenyltransferases Identify an Evolutionarily Conserved GFPPS Clade in Brassicaceae Plants. Mol Plant. 2016;9(2):195–204. Epub 2015/11/06. doi: 10.1016/j.molp.2015.10.010 . 26537048

[26] Tnimov Z, Abankwa D, Alexandrov K. RhoGDI facilitates geranylgeranyltransferase-I-mediated RhoA prenylation. Biochem Biophys Res Commun. 2014;452(4):967–73. Epub 2014/09/17. doi: 10.1016/j.bbrc.2014.09.024 . 25223799

[27] Zhao D, Liu R, Zhang X, Li F, Wang J, Zhang J, et al. Replication and virulence in pigs of the first African swine fever virus isolated in China. Emerg Microbes Infect. 2019;8(1):438–47. Epub 2019/03/23. doi: 10.1080/22221751.2019.1590128 ; PubMed Central PMCID: PMC6455124. 30898043 PMC6455124

[28] Riera E, Pérez-Núñez D, García-Belmonte R, Miorin L, García-Sastre A, Revilla Y. African Swine Fever Virus Induces STAT1 and STAT2 Degradation to Counteract IFN-I Signaling. Front Microbiol. 2021;12:722952. Epub 2021/09/14. doi: 10.3389/fmicb.2021.722952 ; PubMed Central PMCID: PMC8427279. 34512601 PMC8427279

[29] García-Belmonte R, Pérez-Núñez D, Pittau M, Richt JA, Revilla Y. African Swine Fever Virus Armenia/07 Virulent Strain Controls Interferon Beta Production through the cGAS-STING Pathway. J Virol. 2019;93(12). Epub 2019/03/29. doi: 10.1128/JVI.02298-18 ; PubMed Central PMCID: PMC6613762. 30918080 PMC6613762

[30] Takamatsu HH, Denyer MS, Lacasta A, Stirling CM, Argilaguet JM, Netherton CL, et al. Cellular immunity in ASFV responses. Virus Res. 2013;173(1):110–21. Epub 2012/12/04. doi: 10.1016/j.virusres.2012.11.009 . 23201582

[31] Dixon LK, Islam M, Nash R, Reis AL. African swine fever virus evasion of host defences. Virus Res. 2019;266:25–33. Epub 2019/04/09. doi: 10.1016/j.virusres.2019.04.002 ; PubMed Central PMCID: PMC6505686. 30959069 PMC6505686

[32] Wang X, Wu J, Wu Y, Chen H, Zhang S, Li J, et al. Inhibition of cGAS-STING-TBK1 signaling pathway by DP96R of ASFV China 2018/1. Biochem Biophys Res Commun. 2018;506(3):437–43. Epub 2018/10/24. doi: 10.1016/j.bbrc.2018.10.103 . 30348523

[33] Liu H, Zhu Z, Feng T, Ma Z, Xue Q, Wu P, et al. African Swine Fever Virus E120R Protein Inhibits Interferon Beta Production by Interacting with IRF3 To Block Its Activation. J Virol. 2021;95(18):e0082421. Epub 2021/07/01. doi: 10.1128/JVI.00824-21 ; PubMed Central PMCID: PMC8387055. 34190598 PMC8387055

[34] Stempel M, Chan B, Juranić Lisnić V, Krmpotić A, Hartung J, Paludan SR, et al. The herpesviral antagonist m152 reveals differential activation of STING-dependent IRF and NF-κB signaling and STING’s dual role during MCMV infection. Embo j. 2019;38(5). Epub 2019/01/31. doi: 10.15252/embj.2018100983 ; PubMed Central PMCID: PMC6396373. 30696688 PMC6396373

[35] Deschamps T, Kalamvoki M. Evasion of the STING DNA-Sensing Pathway by VP11/12 of Herpes Simplex Virus 1. J Virol. 2017;91(16). Epub 2017/06/09. doi: 10.1128/JVI.00535-17 ; PubMed Central PMCID: PMC5533902. 28592536 PMC5533902

[36] Kumari P, Saha I, Narayanan A, Narayanan S, Takaoka A, Kumar NS, et al. Essential role of HCMV deubiquitinase in promoting oncogenesis by targeting anti-viral innate immune signaling pathways. Cell Death Dis. 2017;8(10):e3078-e. doi: 10.1038/cddis.2017.461 28981114 PMC5680583

[37] Li D, Yang W, Li L, Li P, Ma Z, Zhang J, et al. African Swine Fever Virus MGF-505-7R Negatively Regulates cGAS-STING-Mediated Signaling Pathway. J Immunol. 2021;206(8):1844–57. Epub 2021/03/14. doi: 10.4049/jimmunol.2001110 ; PubMed Central PMCID: PMC8023146. 33712518 PMC8023146

[38] Grant A, Ponia SS, Tripathi S, Balasubramaniam V, Miorin L, Sourisseau M, et al. Zika Virus Targets Human STAT2 to Inhibit Type I Interferon Signaling. Cell Host Microbe. 2016;19(6):882–90. Epub 2016/05/24. doi: 10.1016/j.chom.2016.05.009 ; PubMed Central PMCID: PMC4900918. 27212660 PMC4900918

[39] Sen A, Rott L, Phan N, Mukherjee G, Greenberg HB. Rotavirus NSP1 protein inhibits interferon-mediated STAT1 activation. J Virol. 2014;88(1):41–53. Epub 2013/10/18. doi: 10.1128/JVI.01501-13 ; PubMed Central PMCID: PMC3911692. 24131713 PMC3911692

[40] Zhang K, Yang B, Shen C, Zhang T, Hao Y, Zhang D, et al. MGF360-9L Is a Major Virulence Factor Associated with the African Swine Fever Virus by Antagonizing the JAK/STAT Signaling Pathway. mBio. 2022;13(1):e0233021. Epub 2022/01/26. doi: 10.1128/mbio.02330-21 ; PubMed Central PMCID: PMC8788333. 35076286 PMC8788333

[41] Song S, Cong W, Zhou S, Shi Y, Dai W, Zhang H, et al. Small GTPases: Structure, biological function and its interaction with nanoparticles. Asian J Pharm Sci. 2019;14(1):30–9. doi: 10.1016/j.ajps.2018.06.004 ; PubMed Central PMCID: PMC PMC7032109. 32104436 PMC7032109

[42] Quetglas JI, Hernáez B, Galindo I, Muñoz-Moreno R, Cuesta-Geijo MA, Alonso C. Small rho GTPases and cholesterol biosynthetic pathway intermediates in African swine fever virus infection. J Virol. 2012;86(3):1758–67. Epub 2011/11/25. doi: 10.1128/JVI.05666-11 ; PubMed Central PMCID: PMC3264358. 22114329 PMC3264358

[43] Slavin I, García IA, Monetta P, Martinez H, Romero N, Alvarez C. Role of Rab1b in COPII dynamics and function. Eur J Cell Biol. 2011;90(4):301–11. Epub 2010/11/26. doi: 10.1016/j.ejcb.2010.10.001 . 21093099

[44] Monetta P, Slavin I, Romero N, Alvarez C. Rab1b interacts with GBF1 and modulates both ARF1 dynamics and COPI association. Mol Biol Cell. 2007;18(7):2400–10. Epub 2007/04/13. doi: 10.1091/mbc.e06-11-1005 ; PubMed Central PMCID: PMC1924811. 17429068 PMC1924811

[45] García IA, Martinez HE, Alvarez C. Rab1b regulates COPI and COPII dynamics in mammalian cells. Cell Logist. 2011;1(4):159–63. Epub 2012/01/27. doi: 10.4161/cl.1.4.18221 ; PubMed Central PMCID: PMC3265928. 22279615 PMC3265928

[46] Beachboard DC, Park M, Vijayan M, Snider DL, Fernando DJ, Williams GD, et al. The small GTPase RAB1B promotes antiviral innate immunity by interacting with TNF receptor-associated factor 3 (TRAF3). J Biol Chem. 2019;294(39):14231–40. Epub 2019/08/04. doi: 10.1074/jbc.RA119.007917 . 31375559 PMC6768648

[47] Ran Y, Li D, Xiong MG, Liu HN, Feng T, Shi ZW, et al. African swine fever virus I267L acts as an important virulence factor by inhibiting RNA polymerase III-RIG-I-mediated innate immunity. PLoS Pathog. 2022;18(1):e1010270. Epub 2022/01/29. doi: 10.1371/journal.ppat.1010270 . 35089988 PMC8827485

[48] O’Donnell V, Holinka LG, Gladue DP, Sanford B, Krug PW, Lu X, et al. African Swine Fever Virus Georgia Isolate Harboring Deletions of MGF360 and MGF505 Genes Is Attenuated in Swine and Confers Protection against Challenge with Virulent Parental Virus. J Virol. 2015;89(11):6048–56. Epub 2015/03/27. doi: 10.1128/JVI.00554-15 ; PubMed Central PMCID: PMC4442422. 25810553 PMC4442422

[49] Li J, Song J, Kang L, Huang L, Zhou S, Hu L, et al. pMGF505-7R determines pathogenicity of African swine fever virus infection by inhibiting IL-1β and type I IFN production. PLoS Pathog. 2021;17(7):e1009733. Epub 2021/07/27. doi: 10.1371/journal.ppat.1009733 . 34310655 PMC8341718

[50] Huang L, Liu H, Zhang K, Meng Q, Hu L, Zhang Y, et al. Ubiquitin-Conjugating Enzyme 2S Enhances Viral Replication by Inhibiting Type I IFN Production through Recruiting USP15 to Deubiquitinate TBK1. Cell Rep. 2020;32(7):108044. Epub 2020/08/20. doi: 10.1016/j.celrep.2020.108044 . 32814047

