# Supplementary figures and images for: African swine fever virus pB318L, a trans-geranylgeranyl-diphosphate synthase, negatively regulates cGAS-STING and IFNAR-JAK-STAT signaling pathways
Source: PLoS Pathog. 2024 Apr 15;20(4):e1012136. doi: 10.1371/journal.ppat.1012136 (PMC11018288; doi:10.1371/journal.ppat.1012136)

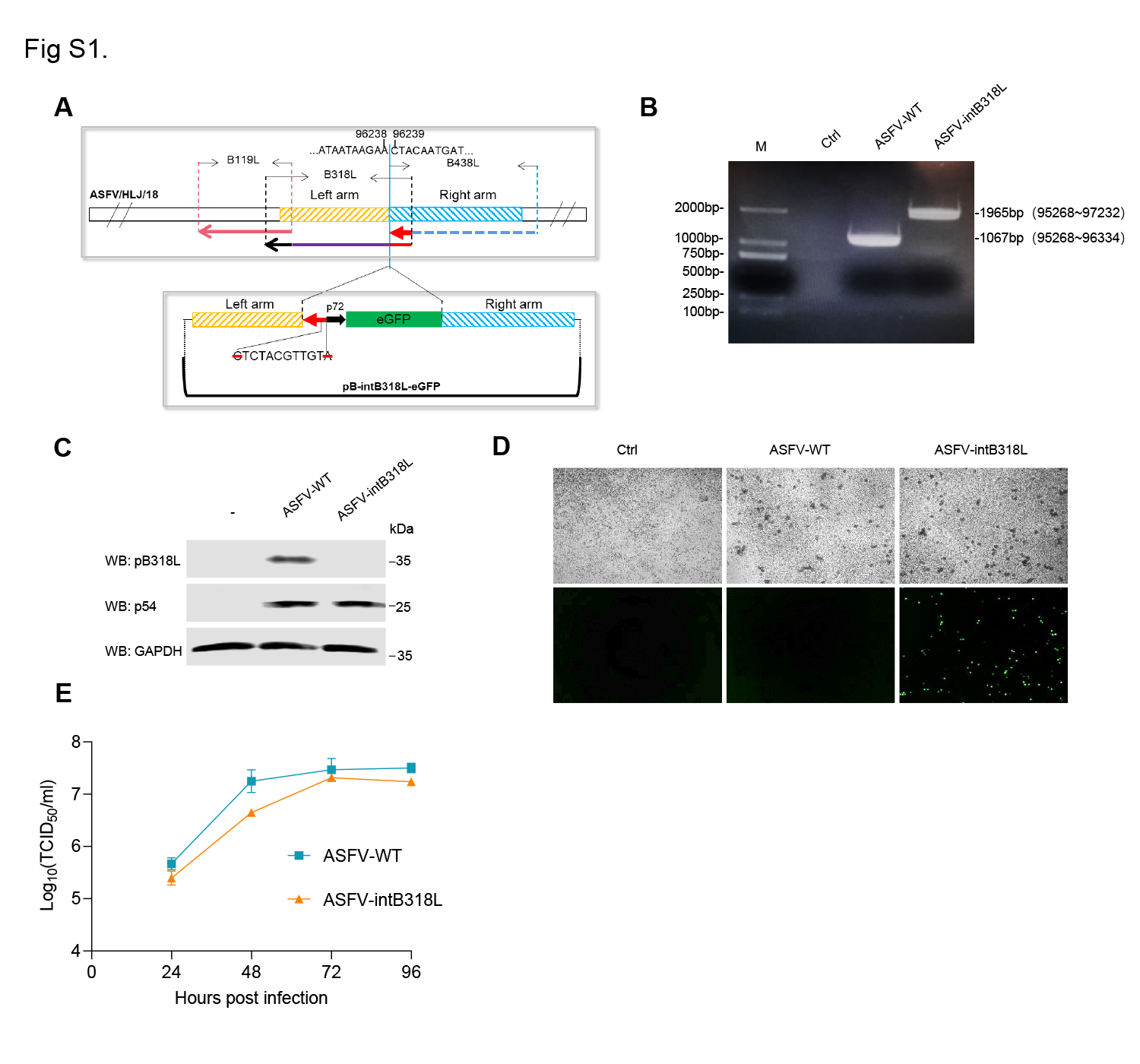

Supplement: S1 Fig — (A) Schematic representation of the generation of ASFV-intB318L virus. (B) PCR verification of the insertion of the p72-EGFP reporter gene cassette in the genome of ASFV-intB318L virus. PAMs infected with ASFV-WT or ASFV-intB318L, at 24 hpi, the virus DNA was extracted and amplified with primers (sequences in ASFV B119L and ASFV B438L respectively). (C) PAMs were infected with ASFV-WT or ASFV-intB318L (MOI = 1). At 24 hpi, cells were lysed and the expression of pB318L was detected by Western blotting. (D) PAMs were infected with ASFV-WT or ASFV-intB318L. At 24 hpi, the cells were observed by microscope for GFP expression. (E) Growth kinetics of ASFV-WT and ASFV-intB318L in PAMs. PAMs were infected with ASFV-intB318L or ASFV-WT, and the TCID50 was monitored at 24, 48, 72, and 96 hpi, respectively. (TIF) [file ppat.1012136.s001.tif]

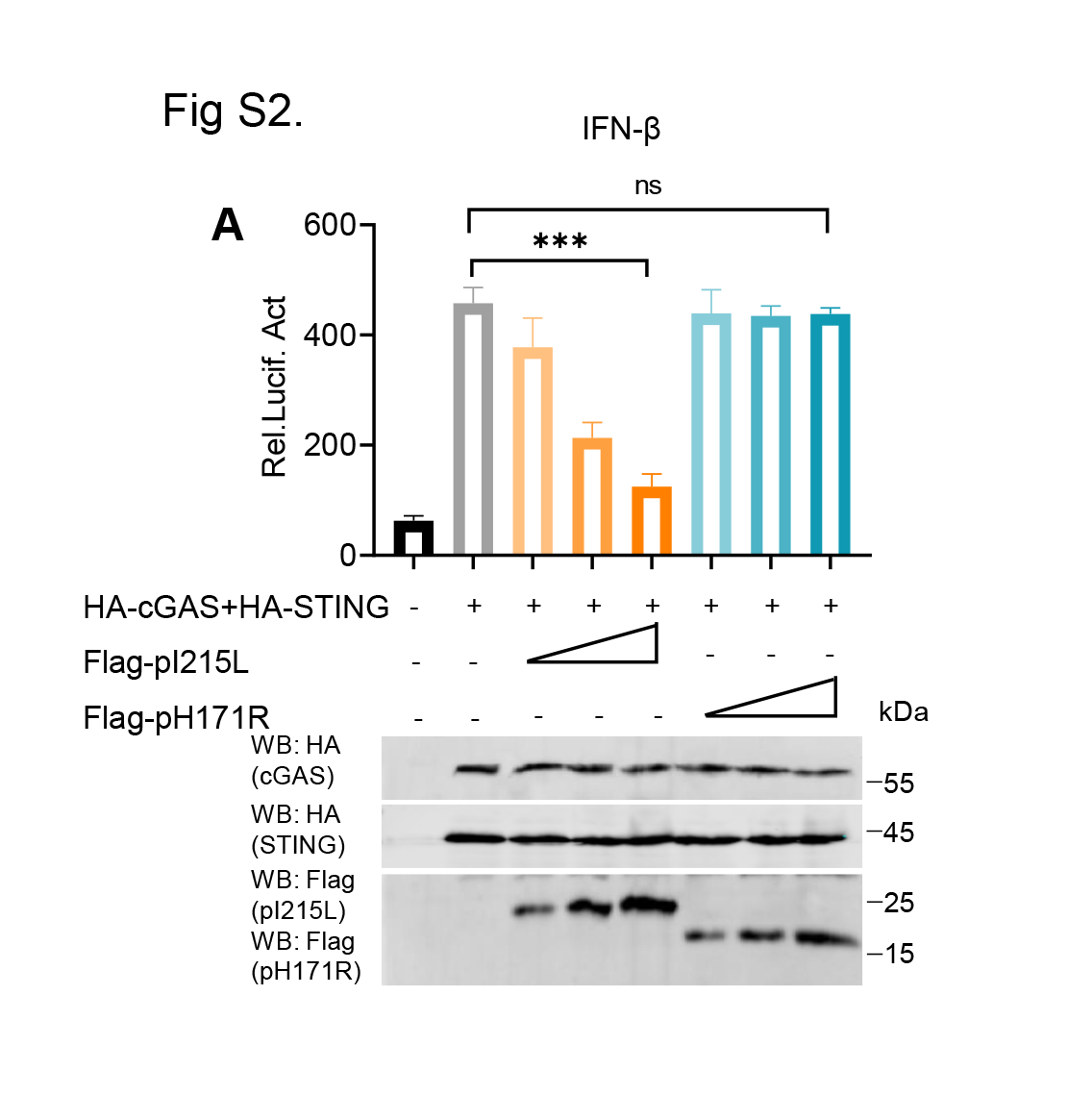

Supplement: S2 Fig — (A) HEK293T cells were transfected with an IFN-β-Luc reporter, a Renilla-TK reporter, and plasmids expressing HA-cGAS and HA-STING, together with increasing amounts (100 ng, 200 ng, 400 ng) of a plasmid expressing Flag-I215L or Flag-H117R. Luciferase activities were analyzed at 24 hpt. Expressions of the proteins were analyzed by Western Blotting. Data are representative of three independent experiments with three biological replicates (mean ± s.d.). Ns, not significantly, *** p < 0.001 (one-way ANOVA). (TIF) [file ppat.1012136.s002.tif]

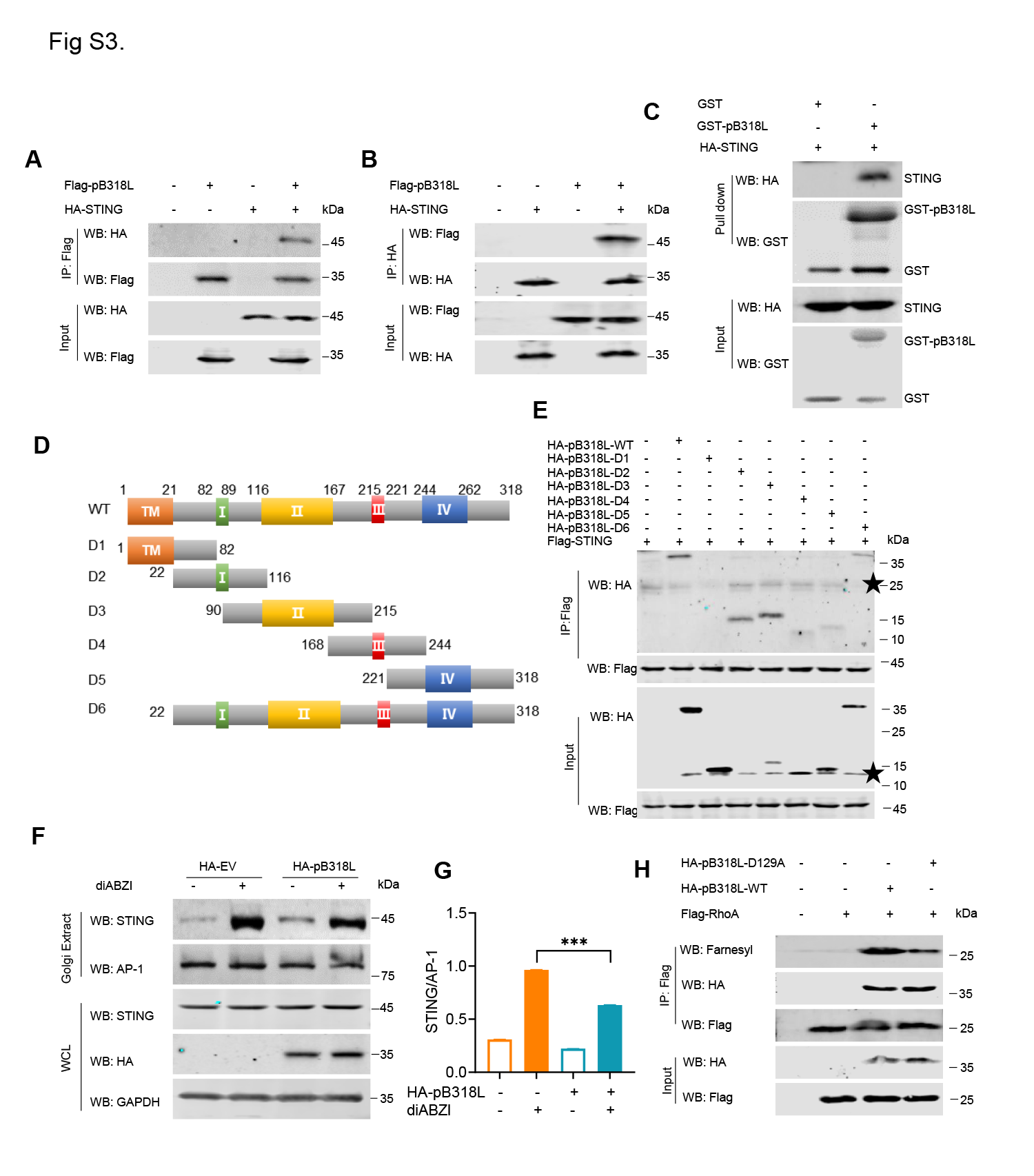

Supplement: S3 Fig — (A) HEK293T cells were transfected with plasmids expressing Flag-pB318L and HA-STING. Co-IP analysis was performed using Flag-beads to detect the interaction between pB318L and STING after 24 h. (B) HEK293T cells were transfected with plasmids expressing Flag-pB318L and HA-STING. Co-IP analysis was performed using HA-beads to detect the interaction between pB318L and STING after 24 h. (C) Direct interaction between STING and ASFV pB318L was detected by GST pull-down assay. (D) Schematic diagram of full-length pB318L and its truncated mutants. (E) HEK293T cells were transfected with a plasmid expressing Flag-STING (2 μg), along with plasmids expressing HA-pB318L-WT, HA-pB318L-D1, HA-pB318L-D2, HA-pB318L-D3, HA-pB318L-D4, HA-pB318L-D5, HA-pB318L-D6 (2 μg/each), respectively. The cells were collected at 24 hpt, and the interactions of pB318L and its deleted mutants with STING were analyzed by Co-IP and Western blotting. The asterisk represents non-specific bands. (F-G) HeLa cells were transfected with HA-pB318L or HA-vector for 24 h, and stimulated with STING agonist for an extra 6 h. Then, the cells were harvested and treated with Golgi Apparatus Enrichment reagent to detect STING on Golgi (F). The quantitation ratio of STING on Golgi was analyzed with image J (G). (H) HEK293T cells were transfected with plasmids expressing Flag-Rho A and HA-pB318L or HA-pB318L-D129A. Co-IP analysis was performed to detect prenylation of Rho A after 24 h. Data are representative of three independent experiments with three biological replicates (mean ± s.d.). *** p < 0.001 (one-way ANOVA). (TIF) [file ppat.1012136.s003.tif]

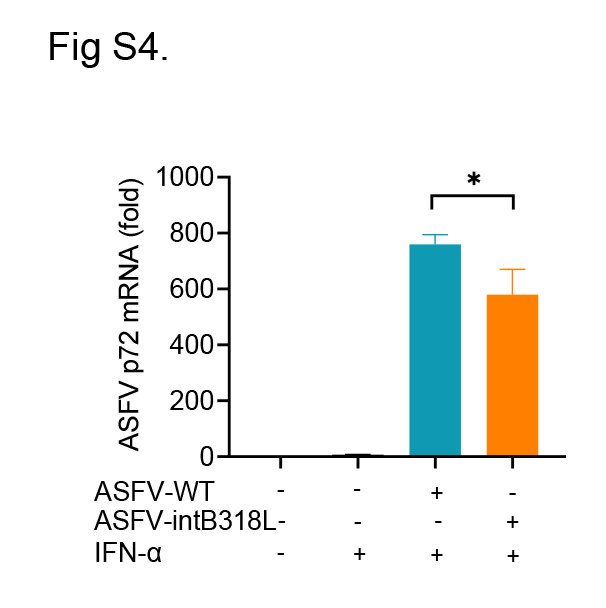

Supplement: S4 Fig — PAMs were infected with ASFV-WT or ASFV-intB318L (MOI = 1) for 24 h, and then treated with IFN-α (1 μg/mL) for another 12 h. The mRNA levels of ASFV p72 were analyzed by qPCR. Data represent three independent experiments with three biological replicates (mean ± s.d.). * p < 0.05 (one-way ANOVA). (TIF) [file ppat.1012136.s004.tif]

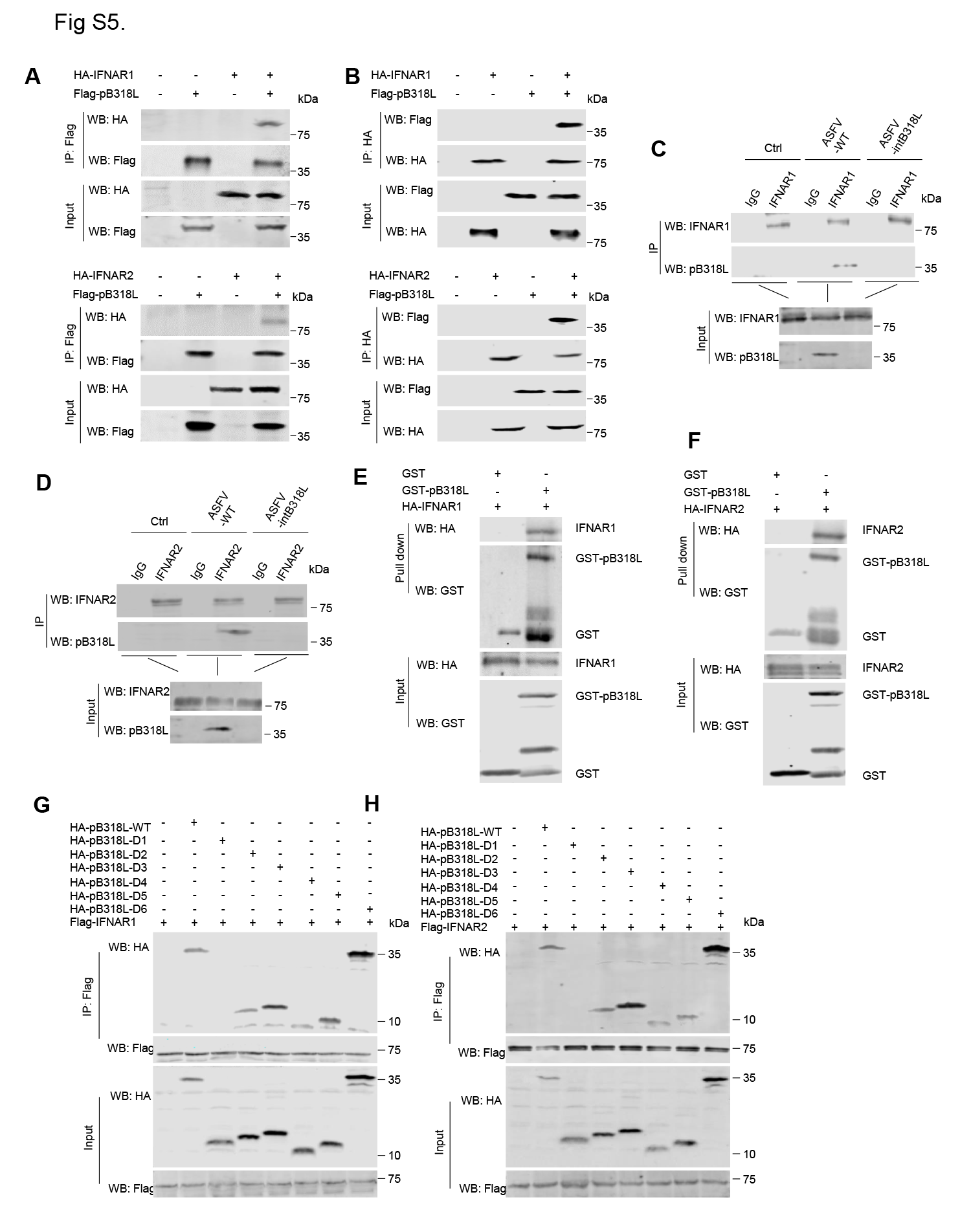

Supplement: S5 Fig — (A) HEK293T cells were transfected with plasmids expressing HA-IFNAR1 (up) or HA-IFNAR2 (down) and Flag-pB318L. At 24 hpt, the cells were lysed and combined with Flag-beads, and the interaction between pB318L and IFNAR1 was detected by Co-IP. (B) HEK293T cells were transfected with plasmids expressing HA-IFNAR1 (up) or HA-IFNAR2 (down) and Flag-pB318L. At 24 hpt, the cells were lysed and combined with HA beads, and the interaction between pB318L and IFNAR1 was detected by Co-IP. (C-D) PAMs were mock infected or infected with ASFV or ASFV-intB318L. At 24 hpt, cells are lysed and incubated with antibodies against IFNAR1 (C) or IFNAR2 (D), and the interaction between pB318L and endogenous IFNAR1/IFNAR2 was detected by Co-IP. (E-F) HEK293T cells were transfected with plasmids expressing HA-IFNAR1 (E) or HA-IFNAR2 (F), at 24 hpt, the cells are lysed and incubated with GST or GST-pB318L. Direct interaction of IFNAR1 or IFNAR2 with ASFV pB318L was detected by GST pull-down assay. (G-H) HEK293T cells were transfected with a plasmid expressing Flag-IFNAR1 (G) or IFNAR2 (H) (2 μg), along with plasmids expressing HA-pB318L-WT, HA-pB318L-D1, HA-pB318L-D2, HA-pB318L-D3, HA-pB318L-D4, HA-pB318L-D5, HA-pB318L-D6 (2 μg), respectively. The cells were collected at 24 hpt and the interactions of pB318L and its deleted mutants with IFNAR1 or IFNAR2 were analyzed by Co-IP and Western blotting. (TIF) [file ppat.1012136.s005.tif]

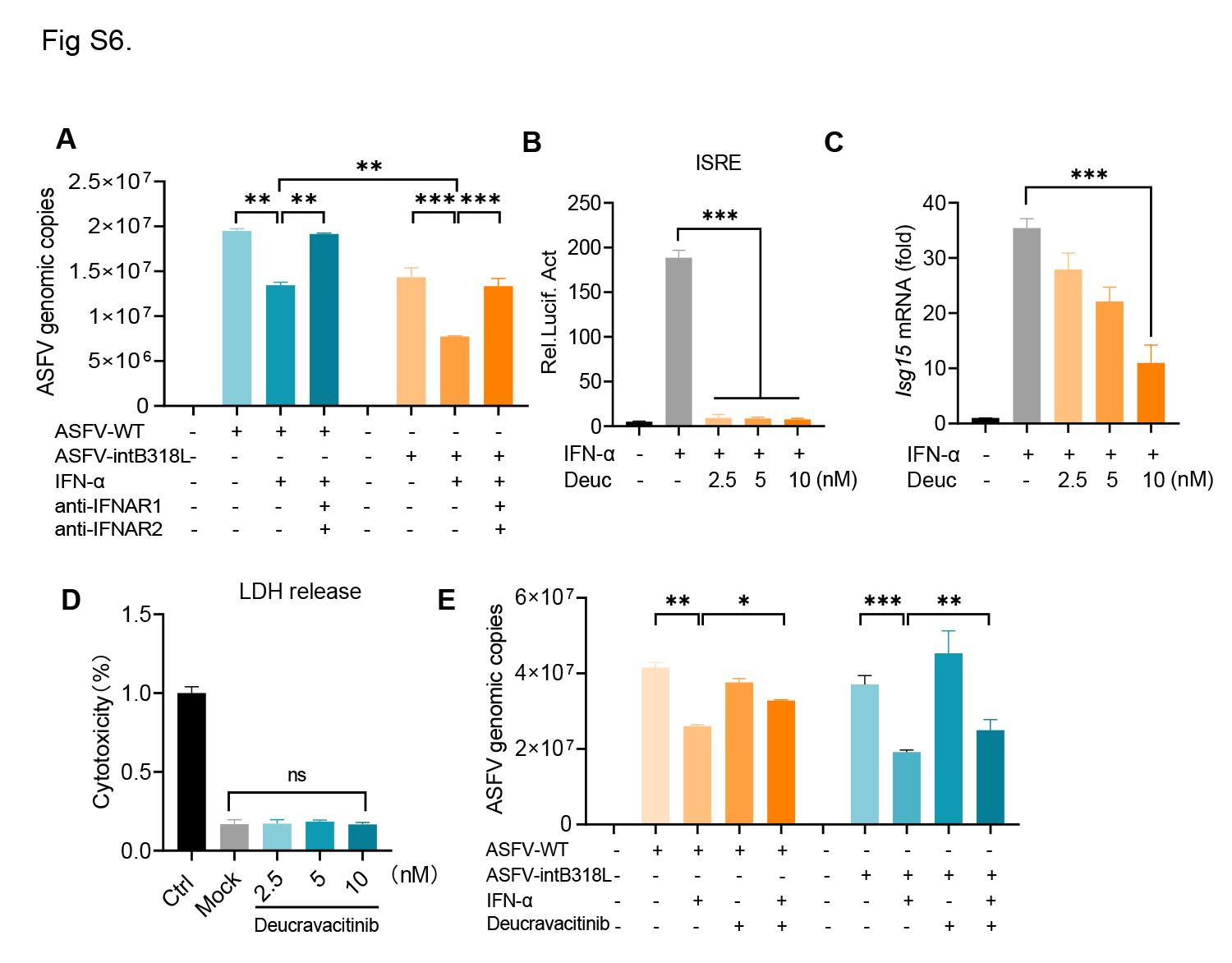

Supplement: S6 Fig — (A) PAMs were pre-incubated with anti-IFNAR1 and anti-IFNAR2 antibodies for 2 h, followed by treatment with IFN-α (1 μg/mL) for 12 h, and then infected with ASFV-WT or ASFV-intB318L. At 24 hpi, qPCR was performed to analyze the ASFV genomic DNA copy numbers in the cells. (B) HEK293T cells were transfected with an ISRE-Luc reporter and a Renilla-TK reporter. At 24 hpt, the cells were treated with different doses of deucravacitinib (2.5 nM, 5 nM, and 10 nM) for 6 h, and then treated with IFN-α (1 μg/mL) for another 12 h. After that, the luciferase activities were analyzed. (C) PAMs were treated with different doses of deucravacitinib (2.5 nM, 5 nM, and 10 nM) for 6 h, and then treated with IFN-α (1 μg/mL) for another 12 h. The mRNA levels of Isg15 were analyzed by qPCR. (D) PAMs were treated with different doses of deucravacitinib (2.5 nM, 5 nM, and 10 nM) for 24 h; LDH assay was then performed according to the manufacturer’s instructions. (E) PAMs were pretreated with deucravacitinib (10 nM) for 6 h, followed by treatment with IFN-α (1 μg/mL) for 12 h, and then infected with ASFV-WT or ASFV-intB318L. At 24 hpi, qPCR was performed to detect ASFV genomic DNA copy numbers in the cells. Data are representative of three independent experiments with three biological replicates (mean ± s.d.). Ns, not significantly, * p < 0.05, ** p < 0.01, *** p < 0.001 (one-way ANOVA). (TIF) [file ppat.1012136.s006.tif]

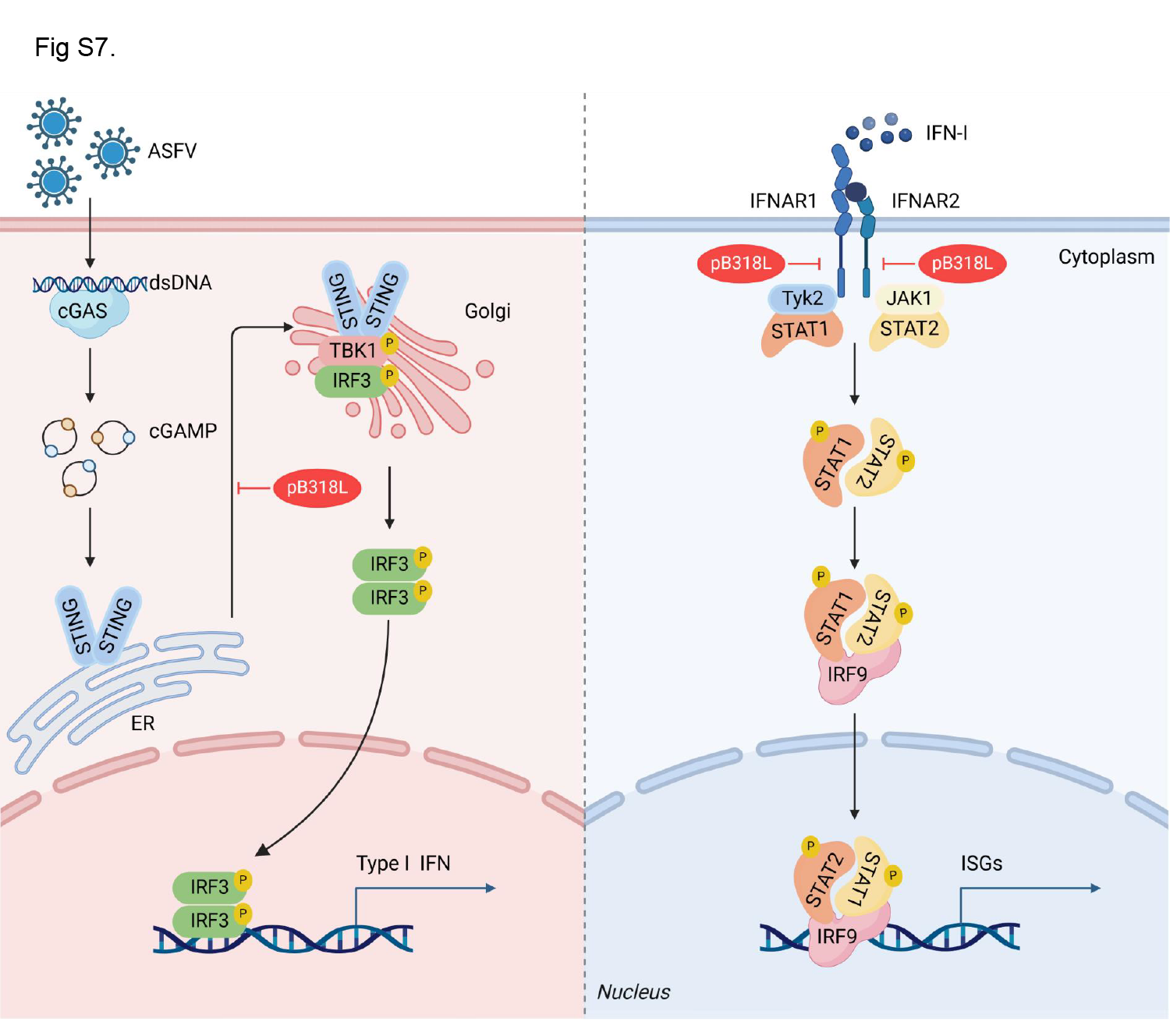

Supplement: S7 Fig — Following ASFV infection, host cGAS senses ASFV genomic DNA, which promotes cGAMP production. cGAMP binds to STING to promote STING activation and translocation from the ER to the Golgi apparatus. Active TBK1 phosphorylates IRF3, promotes the translocation of IRF3 to the nucleus, and initiates the transcription of IFN-I. Secreted IFN-I binds to IFNAR1 and IFNAR2, resulting in activation of JAK1 and TYK2 kinases. The phosphorylated STAT1/2 and IRF9 form a heterotrimeric complex ISGF3. Subsequently, ISGF3 enters the nucleus to bind ISRE to regulate the transcription of ISGs. ASFV pB318L interacts with STING and inhibits the transfer of STING from the endoplasmic reticulum to the Golgi apparatus, thereby inhibiting IFN-I production. On the other side, pB318L binds to IFNAR1/IFNAR2 and blocks the phosphorylation of TYK2 and JAK1, thereby negatively inhibiting IFN-mediated ISGs production. (TIF) [file ppat.1012136.s007.tif]
