# Supplementary material for: African swine fever virus pB318L, a trans-geranylgeranyl-diphosphate synthase, negatively regulates cGAS-STING and IFNAR-JAK-STAT signaling pathways
Source: PLoS Pathog. 2024 Apr 15;20(4):e1012136. doi: 10.1371/journal.ppat.1012136 (PMC11018288; doi:10.1371/journal.ppat.1012136)
Supplement: S1 Table — (DOCX) [file ppat.1012136.s008.docx]

**S1 Table. The DNA sequence covering the modified region of ASFV**

| Recombinant ASFV | Sequence (5’-3’) |
| --- | --- |
| ASFV-ΔB318L | GCCTCCTCGGTACCGTTAACAAGCGCGGAAAAGGACCATTCCCTGCGGGGAGACAACTCAGCATGTTGCATCTCATCTATATCTCCATCATTGTAGATAACTTCGTATAATGTATGCTATACGAAGTTAT**TTACTTGTACAGCTCGTCCATGCCGAGAGTGATCCCGGCGGCGGTCACGAACTCCAGCAGGACCATGTGATCGCGCTTCTCGTTGGGGTCTTTGCTCAGGGCGGACTGGGTGCTCAGGTAGTGGTTGTCGGGCAGCAGCACGGGGCCGTCGCCGATGGGGGTGTTCTGCTGGTAGTGGTCGGCGAGCTGCACGCTGCCGTCCTCGATGTTGTGGCGGATCTTGAAGTTCACCTTGATGCCGTTCTTCTGCTTGTCGGCCATGATATAGACGTTGTGGCTGTTGTAGTTGTACTCCAGCTTGTGCCCCAGGATGTTGCCGTCCTCCTTGAAGTCGATGCCCTTCAGCTCGATGCGGTTCACCAGGGTGTCGCCCTCGAACTTCACCTCGGCGCGGGTCTTGTAGTTGCCGTCGTCCTTGAAGAAGATGGTGCGCTCCTGGACGTAGCCTTCGGGCATGGCGGACTTGAAGAAGTCGTGCTGCTTCATGTGGTCGGGGTAGCGGCTGAAGCACTGCACGCCGTAGGTCAGGGTGGTCACGAGGGTGGGCCAGGGCACGGGCAGCTTGCCGGTGGTGCAGATGAACTTCAGGGTCAGCTTGCCGTAGGTGGCATCGCCCTCGCCCTCGCCGGACACGCTGAACTTGTGGCCGTTTACGTCGCCGTCCAGCTCGACCAGGATGGGCACCACCCCGGTGAACAGCTCCTCGCCCTTGCTCACCAT**GGTGGC**TATATAATGTTATAAAAATAATTTATTGTTTTTATTAAATATGGCGGTTTATGCGAAGGATCTTGATAATAACAAA**TAACTTCGTATAATGTATGCTATACGAAGTTAT***TGTTGCAT*TCATCTATATCTCCATCATTGTAGTTCTTATTATTATTTTAATATCCTATACGCGCAAACCTAAGTATTTTAGAATCACAGCACCGCGTAGTGTCGCGCTTTTTCATGGAATACATCCACTGAATCCTAAAAATTATAAAACCTTTAGTGAAGAGTTTGAGACCATCTTAAATAATGCTATTGAAGATGGGGACTTTAAGGGACAACTAACAGAGCCTTGCTCTTACGCTCTAAGAGGTGGGAAGTATATCCGTCCCATTATCCTGATGGAAATTGTTCGGGCCTGTCAGTTGCAACATTCCTTTGGGGCGCCTATTTATCCGGCAGAGGCCGCCCTAGCAGCGGAGTACTTTCACGTAGCTTCTTTGATTATCGATGATATGCCCTCCTTTGACAACGATGTGAAGCGGCGAAATAAAGATACAGTGTGGGCCCGCTTTGGCGTAGCCAAGGCGCAGATGAGCGCTCTGGCGCTAACCATGCAAGGATTTCAAAATATTTGCCGGCAAATCGACTGGATTAAGGAACACTGTCCAAGGTTTCCGGATCCCAACCAACTGGGAGCGCTGCTGTGTACCTTTGTAAGCCACTCTCTAAACAGCGCGGGTTCCGGTCAGTTAGTAGACACTCCAGAAAAAACCATTCCCTTTTTTAAGATCGCGTTTATTATGGGCTGGGTTTTAGGAACTGGATCCGTTGAAGACATTGGGATGATTGAAAGGGCTGCCCATTGTTTTGGACATGCCTTTCAGTTAGCGGATGACATTAAGGACCATGACACAGATACTGGCTGGAATTACGCCAAAATACATGGAAAACAAAAAACATTTGACGATGTGGCGCAATCCCTTCAAGAGTGCAAAAAAATTCTCCATGGAAAAAAAATATTTACCTCTATATGGAATGAGATTTTTCAAAAGGTTATAAATGTTGCATTGGGGACCTAA**ATACTGGCGATCCTTGCATCTATATGCTATCTTTTTTCAGACGCTCC  (**Green: EGFP;** RED: p72 promoter; BLUE: pB318L**)** |
| ASFV-HLJ/18 | GCCTCCTCGGTACCGTTAACAAGCGCGGAAAAGGACCATTCCCTGCGGGGAGACAACTCAGC**ATGTTGCATCTCATCTATATCTCCATCATTGTAGTTCTTATTATTATTTTAATATCCTATACGCGCAAACCTAAGTATTTTAGAATCACAGCACCGCGTAGTGTCGCGCTTTTTCATGGAATACATCCACTGAATCCTAAAAATTATAAAACCTTTAGTGAAGAGTTTGAGACCATCTTAAATAATGCTATTGAAGATGGGGACTTTAAGGGACAACTAACAGAGCCTTGCTCTTACGCTCTAAGAGGTGGGAAGTATATCCGTCCCATTATCCTGATGGAAATTGTTCGGGCCTGTCAGTTGCAACATTCCTTTGGGGCGCCTATTTATCCGGCAGAGGCCGCCCTAGCAGCGGAGTACTTTCACGTAGCTTCTTTGATTATCGATGATATGCCCTCCTTTGACAACGATGTGAAGCGGCGAAATAAAGATACAGTGTGGGCCCGCTTTGGCGTAGCCAAGGCGCAGATGAGCGCTCTGGCGCTAACCATGCAAGGATTTCAAAATATTTGCCGGCAAATCGACTGGATTAAGGAACACTGTCCAAGGTTTCCGGATCCCAACCAACTGGGAGCGCTGCTGTGTACCTTTGTAAGCCACTCTCTAAACAGCGCGGGTTCCGGTCAGTTAGTAGACACTCCAGAAAAAACCATTCCCTTTTTTAAGATCGCGTTTATTATGGGCTGGGTTTTAGGAACTGGATCCGTTGAAGACATTGGGATGATTGAAAGGGCTGCCCATTGTTTTGGACATGCCTTTCAGTTAGCGGATGACATTAAGGACCATGACACAGATACTGGCTGGAATTACGCCAAAATACATGGAAAACAAAAAACATTTGACGATGTGGCGCAATCCCTTCAAGAGTGCAAAAAAATTCTCCATGGAAAAAAAATATTTACCTCTATATGGAATGAGATTTTTCAAAAGGTTATAAATGTTGCATTGGGGACCTAA**ATACTGGCGATCCTTGCATCTATATGCTATCTTTTTTTCAGACGCTCC  (BLUE: pB318L) |
