# Supplementary material for: African swine fever virus pB318L, a trans-geranylgeranyl-diphosphate synthase, negatively regulates cGAS-STING and IFNAR-JAK-STAT signaling pathways
Source: PLoS Pathog. 2024 Apr 15;20(4):e1012136. doi: 10.1371/journal.ppat.1012136 (PMC11018288; doi:10.1371/journal.ppat.1012136)
Supplement: S2 Table — (DOCX) [file ppat.1012136.s009.docx]

**S2. Table. Primers used for plasmid construction in this study.**

| Plasmids | Primers (5'-3') |
| --- | --- |
| pCAGGS-HA-cGAS  pCAGGS-HA-STING  pCAGGS-HA-TBK1  pCAGGS-IFNAR1  (Flag, HA) | F: 5'-CCGGAATTCGCAGACTCTTGTGTGCCCGC-3'  R: 5'-CGGGGTACCAAAAATTCAACTCCAATTTATTC-3'  F: 5'-CCGGAATTCATGCCCCACTCCAGCCTGCAT-3'  R: 5'-CGGGGTACCTCAAGAGAAATCCGTGCGGAG-3'  F: 5'-CCGGAATTCATGCAGAGCACTTCTAATCATCTTTGG-3'  R: 5'-CGGGGTACCCTAAAGACAGTCAACATTGCGAAGGC-3'  F: 5'-ATGCTCGGGCTTCTGGGTGC-3'  R: 5'-TCACACGGCTGCCTGTCGCAGAA-3' |
| pCAGGS-IFNAR2  (Flag, HA) | F: 5'-ATGCTTTTGAGCCAGAATGTCTCCG-3'  R: 5'-TCATCTCATGATATATCCATCCC-3' |
| pCAGGS-JAK1  (Flag, HA) | F: 5'-ATGGCTTTTTGTGCTAAAA-3'  R: 5'- TTATTTTAAAAGTGCTT-3' |
| pCAGGS-Tyk2  (Flag, HA) | F: 5'- ATGCCTCTGTGCCATTGGGGAGC-3'  R: 5'- TCAGCAGACACTGAACACTGAG-3' |
| pCAGGS-STAT1  (Flag, HA) | F: 5'- ATGTCCCAGTGGTATGAGCT-3'  R: 5'- TTAGTCAAGGTTCATAGTTCCAG-3' |
| pCAGGS-STAT2  (Flag, HA) | F: 5'- ATGGCGCAGTGGGAGATGC-3'  R: 5'- CTAGTAGTCAGAAGGAATC-3' |
| PCAGGS-IRF9  (Flag, HA) | F: 5'- ATGGCTTCAGGCAGGGCTCG-3'  R: 5'-TCAAAGCAGATAGGAGGAGCA-3' |
| pCAGGS-Flag-B318L  -D129A | F1: 5'-TTCGAGCTCATCGATGGTACCATGTTGCATCTCATCTATATCTCC-3'  R1:5'-TGTCAAAGGAGGGCATATCGGCGATAATCAAAGAAGCTACGTGAAAGTAC-3'  F2: 5'-GTACTTTCACGTAGCTTCTTTGATTATCGCCGATATGCCCTCCTTTGACA-3'  R2: 5'-CCATAGATCTGCTAGCTCGAGTTAGGTCCCCAATGCAACATTT-3' |
| pCAGGS-Flag-B318L  -D135A | F1: 5'-TTCGAGCTCATCGATGGTACCATGTTGCATCTCATCTATATCTCC-3'  R1:5'-ATTTCGCCGCTTCACATCGTTGGCAAAGGAGGGCATATCGTCGATAATCAAA-3'  F2:5'-TTTGATTATCGACGATATGCCCTCCTTTGCCAACGATGTGAAGCGGCGAAAT-3'  R2: 5'-CCATAGATCTGCTAGCTCGAGTTAGGTCCCCAATGCAACATTT-3' |
| pCAGGS-Flag-B318L  -D212A | F1: 5'-TTCGAGCTCATCGATGGTACCATGTTGCATCTCATCTATATCTCC-3'  R1: 5'-TAAAAAAGGGAATGGTTTTTTCTGGAGTGTCTACTAACTGACCGGAACCCG-3'  F2: 5'-CGGGTTCCGGTCAGTTAGTAGCCACTCCAGAAAAAACCATTCCCTTTTTTA-3'  R2: 5'-CCATAGATCTGCTAGCTCGAGTTAGGTCCCCAATGCAACATTT-3' |
| pEGFP-B318L | F: 5'- AGTCCGGACTCAGATCTCGAGATGTTGCATCTCATCTATATCTCC -3' |
|  | R: 5'- GTACCGTCGACTGCAGAATTCTTAGGTCCCCAATGCAACATTT -3' |
| pCAGGS-HA-STING-TM+DD | F: 5'- TTCGAGCTCATCGATGGTACCATGCCCTACTCCAGCCTGCATCCA -3' |
|  | R: 5'- ATTAAGatctgctagctcgagGAGTACGTTCTTGTGGCGCTGAT -3' |
| pCAGGS-HA-STING-ΔCTT | F: 5'-TTCGAGCTCATCGATGGTACCATGCCCTACTCCAGCCTGCATCCA -3' |
|  | R:5'-ATTAAGATCTGCTAGCTCGAGCTCCCTTTCCTCCTGCCGAAGGTG -3' |
| pCAGGS-HA-STING-CBD+DD | F: 5'-TTCGAGCTCATCGATGGTACCTTCAACGTGGCTCATGGACTGGCC -3' |
|  | R:5'-ATTAAGatctgctagctcgagCTCCCTTTCCTCCTGCCGAAGGTG -3' |
| pCAGGS-HA-STING-ΔTM | F: 5'-TTCGAGCTCATCGATGGTACCTTCAACGTGGCTCATGGACTGGCC -3' |
| pGEX-6p-1-B318L  pB-ΔB318L-eGFP | R:5'-ATTAAGatctgctagctcgagTCAGAAGATATCTGAGCGGAGTG -3'  F: 5'-CCCCTGGGATCCCCGGAATTCATGCGCAAACCTAAGTATTTTAGAA-3'  R: 5'-GTCACGATGCGGCCGCTCGAGGGTCCCCAATGCAACATTTATA-3'  F-L: 5'-CCGGGCTGCAGGAATTCGATCATGGTCCTTAATGTCATC-3'  F-R-loxp: 5'-AAGGATCTTGATAATAACAAATAACTTCGTATAATGTATG  CTATACGAAGTTATTGTTGCATCTCATCTATATCTCCATC-3'  F-L-loxp: 5'-CATGGACGAGCTGTACAAGTAAATAACTTCGTATAGCATA  CATTATACGAAGTTATCTACAATGATGGAGATATAG-3'  F-R: 5'-ACGGTATCGATAAGCTTGATAATAGTTCGGTTCCTTCTAC-3'  p72GFP-F: 5'-TTGTTATTATCAAGATCCTTC-3'  p72GFP-F: 5'-TTACTTGTACAGCTCGTCCATG-3' |
