# Supplementary material for: African swine fever virus pB318L, a trans-geranylgeranyl-diphosphate synthase, negatively regulates cGAS-STING and IFNAR-JAK-STAT signaling pathways
Source: PLoS Pathog. 2024 Apr 15;20(4):e1012136. doi: 10.1371/journal.ppat.1012136 (PMC11018288; doi:10.1371/journal.ppat.1012136)
Supplement: S3 Table — (DOCX) [file ppat.1012136.s010.docx]

**S3 Table. Primers used for qPCR in this study.**

| Gene name | Primers | Sequence (5'-3') |
| --- | --- | --- |
| Human-IFN-β | h-IFN-β-F | ATGACCAACAAGTGTCTCCTCC |
|  | h-IFN-β-R | GCTCATGGAAAGAGCTGTAGTG |
| Human-β-actin | h-β-actin-F | CCTTCCTGGGCATGGAGTCCTG |
|  | h-β-actin-R | GGAGCAATGATCTTGATCTTC |
| Human-ISG56 | h-ISG56-F | TTGATGACGATGAAATGCCTGA |
|  | h-ISG56-R | CAGGTCACCAGACTCCTCAC |
| Human-ISG54 | h-ISG54-F | AAGCACCTCAAAGGGCAAAAC |
|  | h-ISG54-R | TCGGCCCATGTGATAGTAGAC |
| Swine-IFN-β | sq-IFN-β-F | AGCACTGGCTGGAATGAAACCG |
|  | sq-IFN-β-R | CTCCAGGTCATCCATCTGCCCA |
| Swine-IFN-α | sq-IFN-α-F | CTGCTGCCTGGAATGAGAGCC |
|  | sq-IFN-α-R | TGACACAGGCTTCCAGGTCCC |
| Swine-β-actin | sq-β-actin-F | TGAGAACAGCTGCATCCACTT |
|  | sq-β-actin-R | CGAAGGCAGCTCGGAGTT |
| Swine-ISG15 | sq-ISG15-F | GGTGCAAAGCTTCAGAGACC |
|  | sq-ISG15-R | GTCAGCCAGACCTCATAGGC |
| Swine-ISG56 | sq-ISG56-F | TCAGAGGTGAGAAGGCTGGT |
|  | sq-ISG56-R | GCTTCCTGCAAGTGTCCTTC |
| Swine-Mx1 | sq-Mx1-F | AGCGCAGTGACACCAGCGAC |
|  | sq-Mx1-R | GCCCGGTTCAGCCTGGGAAC |
| Swine-OAS2 | sq-OAS2-F | CACAGCTCAGGGATTTCAGA |
|  | sq-OAS2-R | TCCAACGACAGGGTTTGTAA |
| ASFV-B646L | ASFV-B646L-F | CTGCTCATGGTATCAATCTTATCGA |
|  | ASFV-B646L-R | GATACCACAAGATCAGCCGT |
|  | Probe | CCACGGGAGGAATACCAACCCAGTG |
