# Supplementary material for: African swine fever virus pB318L, a trans-geranylgeranyl-diphosphate synthase, negatively regulates cGAS-STING and IFNAR-JAK-STAT signaling pathways
Source: PLoS Pathog. 2024 Apr 15;20(4):e1012136. doi: 10.1371/journal.ppat.1012136 (PMC11018288; doi:10.1371/journal.ppat.1012136)
Supplement: S3 Appendix — (DOCX) [file ppat.1012136.s013.docx]

# Further validated by PCR amplification and sequencing of the Four mutations

## Sites 14500-15000 (contains mutation sites 14720-14721, with red letter and green background)

ASFV-intB318L TGGTAAAATTTGTTTCGTATAAAAGATGGAAAGGGGTCGATTTAAAACTGGCTGTGCTAA 14560

ASFV-HLJ/18 TGGTAAAATTTGTTTCGTATAAAAGATGGAAAGGGGTCGATTTAAAACTGGCTGTGCTAA 14560

************************************************************

ASFV-intB318L CCAAACCAAAATACTCAAAAGAACGAAAAGTTTCATGGTTGTACTCAGACGCAGATTCTT 14620

ASFV-HLJ/18 CCAAACCAAAATACTCAAAAGAACGAAAAGTTTCATGGTTGTACTCAGACGCAGATTCTT 14620

************************************************************

ASFV-intB318L ACAAAGCGCACATACAAAGCAGCCTGTATATGCAATACCAATGATGAAATAGAGACAGTA 14680

ASFV-HLJ/18 ACAAAGCGCACATACAAAGCAGCCTGTATATGCAATACCAATGATGAAATAGAGACAGTA 14680

************************************************************

ASFV-intB318L TTGCTTTATAGATAATTGTTGATGGTCACCCCCCCCCCCCCCCCATGTTTGCATGAATAT 14740

ASFV-HLJ/18 TTGCTTTATAGATAATTGTTGATGGTCACCCCCCCCCCCCCCCCATGTTTGCATGAATAT 14740

************************************************************

ASFV-intB318L TTCATTTCCTGTATAGGGTCTAGGATGTAAACATTCCATGCTAAAGTGATTAGGCATTTT 14800

ASFV-HLJ/18 TTCATTTCCTGTATAGGGTCTAGGATGTAAACATTCCATGCTAAAGTGATTAGGCATTTT 14800

************************************************************

ASFV-intB318L AGATGAAATTTCATATAAACAGGATTGAGTCTTGGAATCACGGAAAACTCTACAGTTTAC 14860

ASFV-HLJ/18 AGATGAAATTTCATATAAACAGGATTGAGTCTTGGAATCACGGAAAACTCTACAGTTTAC 14860

************************************************************

ASFV-intB318L AATAGAATGATTGGAGTCAATGAAACGAGATTCCGTTATCTTATTTTTGCAAATGCCATC 14920

ASFV-HLJ/18 AATAGAATGATTGGAGTCAATGAAACGAGATTCCGTTATCTTATTTTTGCAAATGCCATC 14920

************************************************************

ASFV-intB318L TTGACAGTCCCAACAGAAATCGCATTGTGGTACATACGTACACCAATATGAAAGCTCACT 14980

ASFV-HLJ/18 TTGACAGTCCCAACAGAAATCGCATTGTGGTACATACGTACACCAATATGAAAGCTCACT 14980

************************************************************

ASFV-intB318L CTTGGGAGGATGCTGGGTTC 15000

ASFV-HLJ/18 CTTGGGAGGATGCTGGGTTC 15000

********************

## Sites 18700-19200 (contains mutation sites 19032-19034, with red letter and green background)

ASFV-intB318L TTTAATGTAGCTATATAAGCTTTCATCAAAATTTAATTTTTTTTTATAAAAATACACGAA 18760

ASFV-HLJ/18 TTTAATGTAGCTATATAAGCTTTCATCAAAATTTAATTTTTTTTTATAAAAATACACGAA 18760

************************************************************

ASFV-intB318L TTAAACTAAAGTCTAAACTTTAGTTTGACTATTTGAGTTAATGATGCTTAACTTATCTTC 18820

ASFV-HLJ/18 TTAAACTAAAGTCTAAACTTTAGTTTGACTATTTGAGTTAATGATGCTTAACTTATCTTC 18820

************************************************************

ASFV-intB318L CATGCTTATCAAGGGGGGGTCCTAATAGTTTTGATACTATTGTTGTGGATTGTTGAATAT 18880

ASFV-HLJ/18 CATGCTTATCAAGGGGGGGTCCTAATAGTTTTGATACTATTGTTGTGGATTGTTGAATAT 18880

************************************************************

ASFV-intB318L AATAAATACTTTATAGATGCTGAAATGTTTGAAAATAATAGTACATCAATGTTGTAAGTT 18940

ASFV-HLJ/18 AATAAATACTTTATAGATGCTGAAATGTTTGAAAATAATAGTACATCAATGTTGTAAGTT 18940

************************************************************

ASFV-intB318L TGATCAAAATTTAATTTCTCATAAAAAAGGTACACATCAACATTGCTCATTTAAGTTTCA 19000

ASFV-HLJ/18 TGATCAAAATTTAATTTCTCATAAAAAAGGTACACATCAACATTGCTCATTTAAGTTTCA 19000

************************************************************

ASFV-intB318L TGATGTTTGATTCATTACTTCCTACAATTACTGGGGGGGGGGGGGGGTCTTTAATAGCTT 19060

ASFV-HLJ/18 TGATGTTTGATTCATTACTTCCTACAATTACTGGGGGGGGGGGGGGGTCTTTAATAGCTT 19060

************************************************************

ASFV-intB318L TAGCATTGTTATGGTTTGCTGACTATTATGTAGAATTCATAGAAGCACGTTTAGATAGTA 19120

ASFV-HLJ/18 TAGCATTGTTATGGTTTGCTGACTATTATGTAGAATTCATAGAAGCACGTTTAGATAGTA 19120

************************************************************

ASFV-intB318L ATATCACTGCAGTGTAGATTATGAAATACATACTAAACTAATTTCAGTATATTTTTTTTG 19180

ASFV-HLJ/18 ATATCACTGCAGTGTAGATTATGAAATACATACTAAACTAATTTCAGTATATTTTTTTTG 19180

************************************************************

ASFV-intB318L TTCATATAAGTTAAGGTACA 19200

ASFV-HLJ/18 TTCATATAAGTTAAGGTACA 19200

********************

## Sites 20500-21000 (contains mutation sites 20835-20836, with red letter and green background)

ASFV-intB318L CATGCTGATTAACGATGTTTTTGAGACAACACGTCGTTAAGGACACCATATTGTCTCCAA 20560

ASFV-HLJ/18 CATGCTGATTAACGATGTTTTTGAGACAACACGTCGTTAAGGACACCATATTGTCTCCAA 20560

************************************************************

ASFV-intB318L TTTGTTAGATAAAAGTCTTTACTAAAAAAATAGATTTTTAGTTTTAACAATCGAGATTTT 20620

ASFV-HLJ/18 TTTGTTAGATAAAAGTCTTTACTAAAAAAATAGATTTTTAGTTTTAACAATCGAGATTTT 20620

************************************************************

ASFV-intB318L ATTATTTGGATGCATCATCAAAAAGATTTATAAGTATAAGAGGTTGTATAAGAAAAAAAA 20680

ASFV-HLJ/18 ATTATTTGGATGCATCATCAAAAAGATTTATAAGTATAAGAGGTTGTATAAGAAAAAAAA 20680

************************************************************

ASFV-intB318L TGATGTTATACTATTTATGTTAAAATTTAATTTATCATATAAAAAGTACAGATTTAATCA 20740

ASFV-HLJ/18 TGATGTTATACTATTTATGTTAAAATTTAATTTATCATATAAAAAGTACAGATTTAATCA 20740

************************************************************

ASFV-intB318L GTTGGTTAAACTATTTAGTTAATTAAACTAAATAGTTTAACCATTTAGTCAGACTACTTG 20800

ASFV-HLJ/18 GTTGGTTAAACTATTTAGTTAATTAAACTAAATAGTTTAACCATTTAGTCAGACTACTTG 20800

************************************************************

ASFV-intB318L GTTAGCAATGTTTGAGCTTTCTTCCATTCTTATCCGGGGGGGGGGGTCCTAATCGTTCTA 20860

ASFV-HLJ/18 GTTAGCAATGTTTGAGCTTTCTTCCATTCTTATCCGGGGGGGGGGGTCCTAATCGTTCTA 20860

************************************************************

ASFV-intB318L ATACTATTGTGGATAGTTGAATATAATGAAGACTTTATAGATGCTATAATGATGAATTCT 20920

ASFV-HLJ/18 ATACTATTGTGGATAGTTGAATATAATGAAGACTTTATAGATGCTATAATGATGAATTCT 20920

************************************************************

ASFV-intB318L AGTATGCCTGTATAAAATAATTAACCTTTTTGATCAAAATTTAATTTTTTTATAAAAAGC 20980

ASFV-HLJ/18 AGTATGCCTGTATAAAATAATTAACCTTTTTGATCAAAATTTAATTTTTTTATAAAAAGC 20980

************************************************************

ASFV-intB318L TACAGAGTAGTGTTTTATTA 20100

ASFV-HLJ/18 TACAGAGTAGTGTTTTATTA 20100

********************

## Sites 141300-141800 (contains mutation sites 141551, with red letter and green background)

ASFV-intB318L tttgtcgttaaaaaggtaaaaccagcggattccatcctgcattccatttggttgattacg 141360

ASFV-HLJ/18 tttgtcgttaaaaaggtaaaaccagcggattccatcctgcattccatttggttgattacg 141360

************************************************************

ASFV-intB318L agcctccatttctttttgcaaaaggttattgcgaatgagtaagcagagcttgatggcact 141420

ASFV-HLJ/18 agcctccatttctttttgcaaaaggttattgcgaatgagtaagcagagcttgatggcact 141420

************************************************************

ASFV-intB318L aatctttgtaaggtttaaacttatgcccaattggtcagcaattttttgttgctcctcccg 141480

ASFV-HLJ/18 aatctttgtaaggtttaaacttatgcccaattggtcagcaattttttgttgctcctcccg 141480

************************************************************

ASFV-intB318L tccgcgtgtttcgcatacggctccccggtttagcatgcgaatatcagtaatctcattctt 141540

ASFV-HLJ/18 tccgcgtgtttcgcatacggctccccggtttagcatgcgaatatcagtaatctcattctt 141540

************************************************************

ASFV-intB318L ttttaaaacctggataggtgggcggattttaaatttaagggcctttcccttgctttccat 141600

ASFV-HLJ/18 ttttaaaacc-ggataggtgggcggattttaaatttaagggcctttcccttgctttccat 141599

********** *************************************************

ASFV-intB318L atagcctatgacgatgtcgttttcttttcgtttaacattaatattaagcatataaagcgg 141660

ASFV-HLJ/18 atagcctatgacgatgtcgttttcttttcgtttaacattaatattaagcatataaagcgg 141659

************************************************************

ASFV-intB318L aatttcatgccaggttttatcttctcgcgaggtaataagtcgcacggagtcctccgtggc 141720

ASFV-HLJ/18 aatttcatgccaggttttatcttctcgcgaggtaataagtcgcacggagtcctccgtggc 141719

************************************************************

ASFV-intB318L atagcccactagagtgttgtcatccccaggcacgtggcttataattttaaaaatgtccgg 141780

ASFV-HLJ/18 atagcccactagagtgttgtcatccccaggcacgtggcttataattttaaaaatgtccgg 141779

************************************************************

ASFV-intB318L aaatggctgaatatcttttt 141800

ASFV-HLJ/18 aaatggctgaatatcttttt 141799

********************

# Sequence alignment of ASFV-intB318L and ASFV-Georgia 2007/1 (contains mutation sites 141551, with red letter and green background)

This sequence contains the mutation site 141551 of ASFV-intB318L, which is located at the end of the D1133L gene. This sequence alignment including *D1133L* and its downstream *D339L*

intB318L ttattcatgtcgggagatggctatttaaaaatcatgtcctatttttctttgctcaataag 60

Georgia ttattcatgtcgggagatggctatttaaaaatcatgtcctatttttctttgctcaataag 60

************************************************************

intB318L catccaaatattttcatggcgttttattaattgttcattattgaacgtatcacaaagatc 120

Georgia catccaaatattttcatggcgttttattaattgttcattattgaacgtatcacaaagatc 120

************************************************************

intB318L atttataaattgcagatagtttattatttctttcaagagagtaacaaacattacttcagc 180

Georgia atttataaattgcagatagtttattatttctttcaagagagtaacaaacattacttcagc 180

************************************************************

intB318L agaacatataataggtaattcagtggcgttaaaagaattttgatcttgttgatacgccaa 240

Georgia agaacatataataggtaattcagtggcgttaaaagaattttgatcttgttgatacgccaa 240

************************************************************

intB318L tggcgaggacttaaggagatttgggggtcttgcccaaaaccctaggctgctgttcttgtt 300

Georgia tggcgaggacttaaggagatttgggggtcttgcccaaaaccctaggctgctgttcttgtt 300

************************************************************

intB318L ttttagggcgtcataaagaaatgaaagcacattgcaaggcttaagccgcgacatctcctt 360

Georgia ttttagggcgtcataaagaaatgaaagcacattgcaaggcttaagccgcgacatctcctt 360

************************************************************

intB318L ccccttgggccctttccatatttttagatctaagatctcatccgagcttatagagtaggt 420

Georgia ccccttgggccctttccatatttttagatctaagatctcatccgagcttatagagtaggt 420

************************************************************

intB318L atagtaaagtttttcaaaaaagcatatctgcttgaagtcttttttagaacgactttcaag 480

Georgia atagtaaagtttttcaaaaaagcatatctgcttgaagtcttttttagaacgactttcaag 480

************************************************************

intB318L aagcatttctataatgttaacaagttttgttaggtttaaggcctgttcctgtgtaagctc 540

Georgia aagcatttctataatgttaacaagttttgttaggtttaaggcctgttcctgtgtaagctc 540

************************************************************

intB318L ctcttgcacgtgatagactgaaaaagtgtgcttaggaatgaaaatactccccgtggcact 600

Georgia ctcttgcacgtgatagactgaaaaagtgtgcttaggaatgaaaatactccccgtggcact 600

************************************************************

intB318L ggcctgttgtctgccaggtatatagtacacgctgctgttagcaagctgtaccggcacaat 660

Georgia ggcctgttgtctgccaggtatatagtacacgctgctgttagcaagctgtaccggcacaat 660

************************************************************

intB318L ttgccccacttctgcaacattattttgcgattcggacgagggtatgacaatagttacggg 720

Georgia ttgccccacttctgcaacattattttgcgattcggacgagggtatgacaatagttacggg 720

************************************************************

intB318L ttcagtcaataggctttcgccgagaataatattactgtcatttttaataattttaacggc 780

Georgia ttcagtcaataggctttcgccgagaataatattactgtcatttttaataattttaacggc 780

************************************************************

intB318L cgctattaaatcaaaggcatttaagtaagaaacaacagcagaaaatcttacatgcatata 840

Georgia cgctattaaatcaaaggcatttaagtaagaaacaacagcagaaaatcttacatgcatata 840

************************************************************

intB318L tcctcttccgctattattcgtacgcataataaaacaaggggagcgttgtataacgccagt 900

Georgia tcctcttccgctattattcgtacgcataataaaacaaggggagcgttgtataacgccagt 900

************************************************************

intB318L aatattaagaataaaactgtttttgaaacacttacccacataaatgttttcaagctcctt 960

Georgia aatattaagaataaaactgtttttgaaacacttacccacataaatgttttcaagctcctt 960

************************************************************

intB318L caaaagatgagcctccacatttgtacaaaaattggtaggatcatcaatattcaacgttgt 1020

Georgia caaaagatgagcctccacatttgtacaaaaattggtaggatcatcaatattcaacgttgt 1020

************************************************************

intB318L ctcaaaaattttttggtcgatcatatctataatatattctgtctatttcaatttaaataa 1080

Georgia ctcaaaaattttttggtcgatcatatctataatatattctgtctatttcaatttaaataa 1080

************************************************************

intB318L tatacgaataaataacgagattattttattaaataagcaatggtgtatacactttgtatt 1140

Georgia tatacgaataaataacgagattattttattaaataagcaatggtgtatacactttgtatt 1140

************************************************************

intB318L tactttgagatatactttgtgtatcacaacgtgccctaagatgtgtgcacaagtgacggc 1200

Georgia tactttgagatatactttgtgtatcacaacgtgccctaagatgtgtgcacaagtgacggc 1200

************************************************************

intB318L attttgtcgttaaaaaggtaaaaccagcggattccatcctgcattccatttggttgatta 1260

Georgia attttgtcgttaaaaaggtaaaaccagcggattccatcctgcattccatttggttgatta 1260

************************************************************

intB318L cgagcctccatttctttttgcaaaaggttattgcgaatgagtaagcagagcttgatggca 1320

Georgia cgagcctccatttctttttgcaaaaggttattgcgaatgagtaagcagagcttgatggca 1320

************************************************************

intB318L ctaatctttgtaaggtttaaacttatgcccaattggtcagcaattttttgttgctcctcc 1380

Georgia ctaatctttgtaaggtttaaacttatgcccaattggtcagcaattttttgttgctcctcc 1380

************************************************************

intB318L cgtccgcgtgtttcgcatacggctccccggtttagcatgcgaatatcagtaatctcattc 1440

Georgia cgtccgcgtgtttcgcatacggctccccggtttagcatgcgaatatcagtaatctcattc 1440

************************************************************

intB318L ttttttaaaacctggataggtgggcggattttaaatttaagggcctttcccttgctttcc 1500

Georgia ttttttaaaacctggataggtgggcggattttaaatttaagggcctttcccttgctttcc 1500

************************************************************

intB318L atatagcctatgacgatgtcgttttcttttcgtttaacattaatattaagcatataaagc 1560

Georgia atatagcctatgacgatgtcgttttcttttcgtttaacattaatattaagcatataaagc 1560

************************************************************

intB318L ggaatttcatgccaggttttatcttctcgcgaggtaataagtcgcacggagtcctccgtg 1620

Georgia ggaatttcatgccaggttttatcttctcgcgaggtaataagtcgcacggagtcctccgtg 1620

************************************************************

intB318L gcatagcccactagagtgttgtcatccccaggcacgtggcttataattttaaaaatgtcc 1680

Georgia gcatagcccactagagtgttgtcatccccaggcacgtggcttataattttaaaaatgtcc 1680

************************************************************

intB318L ggaaatggctgaatatctttttttgaaaaagcgatgaaaaactttttataaacctcgaca 1740

Georgia ggaaatggctgaatatctttttttgaaaaagcgatgaaaaactttttataaacctcgaca 1740

************************************************************

intB318L agggcccccatacctgcaagattatctataataagtgcttctagcatcgtatagtgaaat 1800

Georgia agggcccccatacctgcaagattatctataataagtgcttctagcatcgtatagtgaaat 1800

************************************************************

intB318L gaagcggggtagtggatgagtacctgctccattggctcatcctgaaaatccttctgaaac 1860

Georgia gaagcggggtagtggatgagtacctgctccattggctcatcctgaaaatccttctgaaac 1860

************************************************************

intB318L ttttcatacaatacttgaaagggttctttggtctgcgagtgttcgaggtatttggtaata 1920

Georgia ttttcatacaatacttgaaagggttctttggtctgcgagtgttcgaggtatttggtaata 1920

************************************************************

intB318L cggatgctgtgcatcgcgggaggctgaaaatcccgaatatatgtttcaatatctaatacc 1980

Georgia cggatgctgtgcatcgcgggaggctgaaaatcccgaatatatgtttcaatatctaatacc 1980

************************************************************

intB318L ggttcctttttatggttaagcaccgcagcgacgtacaaatgctcaggctttgccggcaca 2040

Georgia ggttcctttttatggttaagcaccgcagcgacgtacaaatgctcaggctttgccggcaca 2040

************************************************************

intB318L tgcataatggtgcaaagacgattctgtatccataattccttgcactggttttttgagtag 2100

Georgia tgcataatggtgcaaagacgattctgtatccataattccttgcactggttttttgagtag 2100

************************************************************

intB318L catagagaaatgagcgccagcgcgaagttgtcctctgagaagagtttattatcgatggta 2160

Georgia catagagaaatgagcgccagcgcgaagttgtcctctgagaagagtttattatcgatggta 2160

************************************************************

intB318L attccctgtatgagcttgggagtggaaacagccttccatagctcggagtacgtccacacg 2220

Georgia attccctgtatgagcttgggagtggaaacagccttccatagctcggagtacgtccacacg 2220

************************************************************

intB318L gggcgtgccataaacaaagatataataatattagaaattgtttttacctcttgctccccg 2280

Georgia gggcgtgccataaacaaagatataataatattagaaattgtttttacctcttgctccccg 2280

************************************************************

intB318L tatccataggcctcaaaggtattgaggacggtggctccgacgtttgccggcgtgatggat 2340

Georgia tatccataggcctcaaaggtattgaggacggtggctccgacgtttgccggcgtgatggat 2340

************************************************************

intB318L ggactaaggggcagactttccaacataggcttatcaatcttaatctggttggtgaaccca 2400

Georgia ggactaaggggcagactttccaacataggcttatcaatcttaatctggttggtgaaccca 2400

************************************************************

intB318L tcaatggcgtgctttcgcagcgccttatccccctcctgtattaaaatgtattcttttaat 2460

Georgia tcaatggcgtgctttcgcagcgccttatccccctcctgtattaaaatgtattcttttaat 2460

************************************************************

intB318L ttttgtgcgtacttagcgagctctggccctccatcgggtgttgtcgatacgtacaaataa 2520

Georgia ttttgtgcgtacttagcgagctctggccctccatcgggtgttgtcgatacgtacaaataa 2520

************************************************************

intB318L attgtcacgttgcgctcactgggggggagctccatgtgtgaattttttcgcaccaccctc 2580

Georgia attgtcacgttgcgctcactgggggggagctccatgtgtgaattttttcgcaccaccctc 2580

************************************************************

intB318L ccaaatacctgaataagccggggaatatcaaggggcaatgacataatcatctcgtaccgc 2640

Georgia ccaaatacctgaataagccggggaatatcaaggggcaatgacataatcatctcgtaccgc 2640

************************************************************

intB318L acggcctgaaagttcaaaccctccacaatcaccttggacccgatgagaatacgcagctgg 2700

Georgia acggcctgaaagttcaaaccctccacaatcaccttggacccgatgagaatacgcagctgg 2700

************************************************************

intB318L tggccttccaggttggacgaggcgttaaaaagagccaggcttcgttcgcgtacagcgggc 2760

Georgia tggccttccaggttggacgaggcgttaaaaagagccaggcttcgttcgcgtacagcgggc 2760

************************************************************

intB318L tctatttcgctgtgcagaatggtgaaccgtactggaataaactgatggtcgctatgtgtg 2820

Georgia tctatttcgctgtgcagaatggtgaaccgtactggaataaactgatggtcgctatgtgtg 2820

************************************************************

intB318L tgctcatcgcgaatcgcggcgcagatggagcagcgggtcgttcccacaggggacgaaact 2880

Georgia tgctcatcgcgaatcgcggcgcagatggagcagcgggtcgttcccacaggggacgaaact 2880

************************************************************

intB318L tcatttaaaatgccattactttgtaaaatttcttgcaagataagaacccccgacatgcgg 2940

Georgia tcatttaaaatgccattactttgtaaaatttcttgcaagataagaacccccgacatgcgg 2940

************************************************************

intB318L acccgattgtggtaaattaaaattttcccccggccttgccgaataatggaaagaatgtct 3000

Georgia acccgattgtggtaaattaaaattttcccccggccttgccgaataatggaaagaatgtct 3000

************************************************************

intB318L ttcatcatttgagtgtattttccgctataaaaggccaatcccgagatgtgcgttggtggc 3060

Georgia ttcatcatttgagtgtattttccgctataaaaggccaatcccgagatgtgcgttggtggc 3060

************************************************************

intB318L tgcagcgacaaaaagctgccactcacattaaagggggctctacgcgaaggctcaataatc 3120

Georgia tgcagcgacaaaaagctgccactcacattaaagggggctctacgcgaaggctcaataatc 3120

************************************************************

intB318L tgtaccccgttttccagaagccagtctgtgcttgccatagaaagggcggtgggggtttcc 3180

Georgia tgtaccccgttttccagaagccagtctgtgcttgccatagaaagggcggtgggggtttcc 3180

************************************************************

intB318L gtcgagttaaacaggccgtaagccttgggttccgtttgttttgaaaattttgggttggga 3240

Georgia gtcgagttaaacaggccgtaagccttgggttccgtttgttttgaaaattttgggttggga 3240

************************************************************

intB318L aacaccatgtcataaatgctgtacgcattactcgagattttagggtcagggcccagctgt 3300

Georgia aacaccatgtcataaatgctgtacgcattactcgagattttagggtcagggcccagctgt 3300

************************************************************

intB318L ttaagcgtttcaagctgatactcagacatggggcattcgatgaaatgtaagtacggcaat 3360

Georgia ttaagcgtttcaagctgatactcagacatggggcattcgatgaaatgtaagtacggcaat 3360

************************************************************

intB318L gtttcgtctttataggacaacatctttccggcaaatattctttcggggtaaaaattggtg 3420

Georgia gtttcgtctttataggacaacatctttccggcaaatattctttcggggtaaaaattggtg 3420

************************************************************

intB318L ttggtatccaacaaaaaagatacccttccggtgctcagtctttccacaagagctagggcg 3480

Georgia ttggtatccaacaaaaaagatacccttccggtgctcagtctttccacaagagctagggcg 3480

************************************************************

intB318L tcctttttccatttaacggaatgcccactgctgtcaaacagttgctggcgctggaggggc 3540

Georgia tcctttttccatttaacggaatgcccactgctgtcaaacagttgctggcgctggaggggc 3540

************************************************************

intB318L tggccgttgggcagctcatgccgcggaaccaaaaggtttaacaggtcgacgtattccatg 3600

Georgia tggccgttgggcagctcatgccgcggaaccaaaaggtttaacaggtcgacgtattccatg 3600

************************************************************

intB318L acactcccggttacgggcgttgccgacatgaagacggccctgggggcctggtgaggtgga 3660

Georgia acactcccggttacgggcgttgccgacatgaagacggccctgggggcctggtgaggtgga 3660

************************************************************

intB318L aaggcatccaggacatactgtaaagcgatgccataattatttcgttcctggatattgtac 3720

Georgia aaggcatccaggacatactgtaaagcgatgccataattatttcgttcctggatattgtac 3720

************************************************************

intB318L acgttgtgtatttcatccgcaatgagcagtcctcccctaagttgctccatgattttttga 3780

Georgia acgttgtgtatttcatccgcaatgagcagtcctcccctaagttgctccatgattttttga 3780

************************************************************

intB318L ttcacccggatgaggccgtttgtctcggcctcgctaattttttgcacgaactgagatata 3840

Georgia ttcacccggatgaggccgtttgtctcggcctcgctaattttttgcacgaactgagatata 3840

************************************************************

intB318L tcgttctcattcaatgtatcttctgcttcgtcagaacgatgaaacagagaaagcacatca 3900

Georgia tcgttctcattcaatgtatcttctgcttcgtcagaacgatgaaacagagaaagcacatca 3900

************************************************************

intB318L aagtttttctcttcacccttactcgtaatattgaaaagcttggatgcaaattccttatag 3960

Georgia aagtttttctcttcacccttactcgtaatattgaaaagcttggatgcaaattccttatag 3960

************************************************************

intB318L ccgtaaaactgaaaaaagcctccgcggtttctatcggttaaacggcgctttaacgtacta 4020

Georgia ccgtaaaactgaaaaaagcctccgcggtttctatcggttaaacggcgctttaacgtacta 4020

************************************************************

intB318L acgaacccatttagatgccgtgattcgaccgacgtggtgctgccagactgctttgcaatg 4080

Georgia acgaacccatttagatgccgtgattcgaccgacgtggtgctgccagactgctttgcaatg 4080

************************************************************

intB318L tgaagaagccggtgtagctcagcgacctccttgtaagaaacaaatcccagctcaggacgt 4140

Georgia tgaagaagccggtgtagctcagcgacctccttgtaagaaacaaatcccagctcaggacgt 4140

************************************************************

intB318L cttagcatttctgtttgaatgatggcgcgtgtaaagcctaccacaaaaatccagggcgca 4200

Georgia cttagcatttctgtttgaatgatggcgcgtgtaaagcctaccacaaaaatccagggcgca 4200

************************************************************

intB318L ttttcaataaaattcatgtagtggttcataaattgacgcgcgatggcaatcgcggcaatg 4260

Georgia ttttcaataaaattcatgtagtggttcataaattgacgcgcgatggcaatcgcggcaatg 4260

************************************************************

intB318L ctttttcccgtcccggtctgccagtttaataaaagacgcgagtagggcgtgttgggattt 4320

Georgia ctttttcccgtcccggtctgccagtttaataaaagacgcgagtagggcgtgttgggattt 4320

************************************************************

intB318L tgaaagttttggacgaaaagctgggcattatgcaattggagacccttgatggaaggaaag 4380

Georgia tgaaagttttggacgaaaagctgggcattatgcaattggagacccttgatggaaggaaag 4380

************************************************************

intB318L ggcgacgcgtaggggtcacacggaaaaaacgctcgcccccccttctcgcagccaggccca 4440

Georgia ggcgacgcgtaggggtcacacggaaaaaacgctcgcccccccttctcgcagccaggccca 4440

************************************************************

intB318L ccgatctggacaaaatgagcccgcagatcacgaatgagctctttttggtcgacaggaggg 4500

Georgia ccgatctggacaaaatgagcccgcagatcacgaatgagctctttttggtcgacaggaggg 4500

************************************************************

intB318L gaaatcaacgatttaaactcctttcttcgcgccaactgctgcaaaaagtctgcggcatcc 4560

Georgia gaaatcaacgatttaaactcctttcttcgcgccaactgctgcaaaaagtctgcggcatcc 4560

************************************************************

intB318L aattcgggatacgccat 4577

Georgia aattcgggatacgccat 4577

*****************

# Sequence alignment of ASFV-intB318L and ASFV-HLJ/18 (contains mutation sites 141551, with red letter and green background)

This sequence contains the mutation site 141551 of ASFV-intB318L, which is located at the end of the D1133L gene. This sequence alignment including *D1133L* and its downstream *D339L*

intB318L ttattcatgtcgggagatggctatttaaaaatcatgtcctatttttctttgctcaataag 60

HLJ/18 ttattcatgtcgggagatggctatttaaaaatcatgtcctatttttctttgctcaataag 60

************************************************************

intB318L catccaaatattttcatggcgttttattaattgttcattattgaacgtatcacaaagatc 120

HLJ/18 catccaaatattttcatggcgttttattaattgttcattattgaacgtatcacaaagatc 120

************************************************************

intB318L atttataaattgcagatagtttattatttctttcaagagagtaacaaacattacttcagc 180

HLJ/18 atttataaattgcagatagtttattatttctttcaagagagtaacaaacattacttcagc 180

************************************************************

intB318L agaacatataataggtaattcagtggcgttaaaagaattttgatcttgttgatacgccaa 240

HLJ/18 agaacatataataggtaattcagtggcgttaaaagaattttgatcttgttgatacgccaa 240

************************************************************

intB318L tggcgaggacttaaggagatttgggggtcttgcccaaaaccctaggctgctgttcttgtt 300

HLJ/18 tggcgaggacttaaggagatttgggggtcttgcccaaaaccctaggctgctgttcttgtt 300

************************************************************

intB318L ttttagggcgtcataaagaaatgaaagcacattgcaaggcttaagccgcgacatctcctt 360

HLJ/18 ttttagggcgtcataaagaaatgaaagcacattgcaaggcttaagccgcgacatctcctt 360

************************************************************

intB318L ccccttgggccctttccatatttttagatctaagatctcatccgagcttatagagtaggt 420

HLJ/18 ccccttgggccctttccatatttttagatctaagatctcatccgagcttatagagtaggt 420

************************************************************

intB318L atagtaaagtttttcaaaaaagcatatctgcttgaagtcttttttagaacgactttcaag 480

HLJ/18 atagtaaagtttttcaaaaaagcatatctgcttgaagtcttttttagaacgactttcaag 480

************************************************************

intB318L aagcatttctataatgttaacaagttttgttaggtttaaggcctgttcctgtgtaagctc 540

HLJ/18 aagcatttctataatgttaacaagttttgttaggtttaaggcctgttcctgtgtaagctc 540

************************************************************

intB318L ctcttgcacgtgatagactgaaaaagtgtgcttaggaatgaaaatactccccgtggcact 600

HLJ/18 ctcttgcacgtgatagactgaaaaagtgtgcttaggaatgaaaatactccccgtggcact 600

************************************************************

intB318L ggcctgttgtctgccaggtatatagtacacgctgctgttagcaagctgtaccggcacaat 660

HLJ/18 ggcctgttgtctgccaggtatatagtacacgctgctgttagcaagctgtaccggcacaat 660

************************************************************

intB318L ttgccccacttctgcaacattattttgcgattcggacgagggtatgacaatagttacggg 720

HLJ/18 ttgccccacttctgcaacattattttgcgattcggacgagggtatgacaatagttacggg 720

************************************************************

intB318L ttcagtcaataggctttcgccgagaataatattactgtcatttttaataattttaacggc 780

HLJ/18 ttcagtcaataggctttcgccgagaataatattactgtcatttttaataattttaacggc 780

************************************************************

intB318L cgctattaaatcaaaggcatttaagtaagaaacaacagcagaaaatcttacatgcatata 840

HLJ/18 cgctattaaatcaaaggcatttaagtaagaaacaacagcagaaaatcttacatgcatata 840

************************************************************

intB318L tcctcttccgctattattcgtacgcataataaaacaaggggagcgttgtataacgccagt 900

HLJ/18 tcctcttccgctattattcgtacgcataataaaacaaggggagcgttgtataacgccagt 900

************************************************************

intB318L aatattaagaataaaactgtttttgaaacacttacccacataaatgttttcaagctcctt 960

HLJ/18 aatattaagaataaaactgtttttgaaacacttacccacataaatgttttcaagctcctt 960

************************************************************

intB318L caaaagatgagcctccacatttgtacaaaaattggtaggatcatcaatattcaacgttgt 1020

HLJ/18 caaaagatgagcctccacatttgtacaaaaattggtaggatcatcaatattcaacgttgt 1020

************************************************************

intB318L ctcaaaaattttttggtcgatcatatctataatatattctgtctatttcaatttaaataa 1080

HLJ/18 ctcaaaaattttttggtcgatcatatctataatatattctgtctatttcaatttaaataa 1080

************************************************************

intB318L tatacgaataaataacgagattattttattaaataagcaatggtgtatacactttgtatt 1140

HLJ/18 tatacgaataaataacgagattattttattaaataagcaatggtgtatacactttgtatt 1140

************************************************************

intB318L tactttgagatatactttgtgtatcacaacgtgccctaagatgtgtgcacaagtgacggc 1200

HLJ/18 tactttgagatatactttgtgtatcacaacgtgccctaagatgtgtgcacaagtgacggc 1200

************************************************************

intB318L attttgtcgttaaaaaggtaaaaccagcggattccatcctgcattccatttggttgatta 1260

HLJ/18 attttgtcgttaaaaaggtaaaaccagcggattccatcctgcattccatttggttgatta 1260

************************************************************

intB318L cgagcctccatttctttttgcaaaaggttattgcgaatgagtaagcagagcttgatggca 1320

HLJ/18 cgagcctccatttctttttgcaaaaggttattgcgaatgagtaagcagagcttgatggca 1320

************************************************************

intB318L ctaatctttgtaaggtttaaacttatgcccaattggtcagcaattttttgttgctcctcc 1380

HLJ/18 ctaatctttgtaaggtttaaacttatgcccaattggtcagcaattttttgttgctcctcc 1380

************************************************************

intB318L cgtccgcgtgtttcgcatacggctccccggtttagcatgcgaatatcagtaatctcattc 1440

HLJ/18 cgtccgcgtgtttcgcatacggctccccggtttagcatgcgaatatcagtaatctcattc 1440

************************************************************

intB318L ttttttaaaacc**t**ggataggtgggcggattttaaatttaagggcctttcccttgctttcc 1500

HLJ/18 ttttttaaaacc-ggataggtgggcggattttaaatttaagggcctttcccttgctttcc 1499

************ ***********************************************

intB318L atatagcctatgacgatgtcgttttcttttcgtttaacattaatattaagcatataaagc 1560

HLJ/18 atatagcctatgacgatgtcgttttcttttcgtttaacattaatattaagcatataaagc 1559

************************************************************

intB318L ggaatttcatgccaggttttatcttctcgcgaggtaataagtcgcacggagtcctccgtg 1620

HLJ/18 ggaatttcatgccaggttttatcttctcgcgaggtaataagtcgcacggagtcctccgtg 1619

************************************************************

intB318L gcatagcccactagagtgttgtcatccccaggcacgtggcttataattttaaaaatgtcc 1680

HLJ/18 gcatagcccactagagtgttgtcatccccaggcacgtggcttataattttaaaaatgtcc 1679

************************************************************

intB318L ggaaatggctgaatatctttttttgaaaaagcgatgaaaaactttttataaacctcgaca 1740

HLJ/18 ggaaatggctgaatatctttttttgaaaaagcgatgaaaaactttttataaacctcgaca 1739

************************************************************

intB318L agggcccccatacctgcaagattatctataataagtgcttctagcatcgtatagtgaaat 1800

HLJ/18 agggcccccatacctgcaagattatctataataagtgcttctagcatcgtatagtgaaat 1799

************************************************************

intB318L gaagcggggtagtggatgagtacctgctccattggctcatcctgaaaatccttctgaaac 1860

HLJ/18 gaagcggggtagtggatgagtacctgctccattggctcatcctgaaaatccttctgaaac 1859

************************************************************

intB318L ttttcatacaatacttgaaagggttctttggtctgcgagtgttcgaggtatttggtaata 1920

HLJ/18 ttttcatacaatacttgaaagggttctttggtctgcgagtgttcgaggtatttggtaata 1919

************************************************************

intB318L cggatgctgtgcatcgcgggaggctgaaaatcccgaatatatgtttcaatatctaatacc 1980

HLJ/18 cggatgctgtgcatcgcgggaggctgaaaatcccgaatatatgtttcaatatctaatacc 1979

************************************************************

intB318L ggttcctttttatggttaagcaccgcagcgacgtacaaatgctcaggctttgccggcaca 2040

HLJ/18 ggttcctttttatggttaagcaccgcagcgacgtacaaatgctcaggctttgccggcaca 2039

************************************************************

intB318L tgcataatggtgcaaagacgattctgtatccataattccttgcactggttttttgagtag 2100

HLJ/18 tgcataatggtgcaaagacgattctgtatccataattccttgcactggttttttgagtag 2099

************************************************************

intB318L catagagaaatgagcgccagcgcgaagttgtcctctgagaagagtttattatcgatggta 2160

HLJ/18 catagagaaatgagcgccagcgcgaagttgtcctctgagaagagtttattatcgatggta 2159

************************************************************

intB318L attccctgtatgagcttgggagtggaaacagccttccatagctcggagtacgtccacacg 2220

HLJ/18 attccctgtatgagcttgggagtggaaacagccttccatagctcggagtacgtccacacg 2219

************************************************************

intB318L gggcgtgccataaacaaagatataataatattagaaattgtttttacctcttgctccccg 2280

HLJ/18 gggcgtgccataaacaaagatataataatattagaaattgtttttacctcttgctccccg 2279

************************************************************

intB318L tatccataggcctcaaaggtattgaggacggtggctccgacgtttgccggcgtgatggat 2340

HLJ/18 tatccataggcctcaaaggtattgaggacggtggctccgacgtttgccggcgtgatggat 2339

************************************************************

intB318L ggactaaggggcagactttccaacataggcttatcaatcttaatctggttggtgaaccca 2400

HLJ/18 ggactaaggggcagactttccaacataggcttatcaatcttaatctggttggtgaaccca 2399

************************************************************

intB318L tcaatggcgtgctttcgcagcgccttatccccctcctgtattaaaatgtattcttttaat 2460

HLJ/18 tcaatggcgtgctttcgcagcgccttatccccctcctgtattaaaatgtattcttttaat 2459

************************************************************

intB318L ttttgtgcgtacttagcgagctctggccctccatcgggtgttgtcgatacgtacaaataa 2520

HLJ/18 ttttgtgcgtacttagcgagctctggccctccatcgggtgttgtcgatacgtacaaataa 2519

************************************************************

intB318L attgtcacgttgcgctcactgggggggagctccatgtgtgaattttttcgcaccaccctc 2580

HLJ/18 attgtcacgttgcgctcactgggggggagctccatgtgtgaattttttcgcaccaccctc 2579

************************************************************

intB318L ccaaatacctgaataagccggggaatatcaaggggcaatgacataatcatctcgtaccgc 2640

HLJ/18 ccaaatacctgaataagccggggaatatcaaggggcaatgacataatcatctcgtaccgc 2639

************************************************************

intB318L acggcctgaaagttcaaaccctccacaatcaccttggacccgatgagaatacgcagctgg 2700

HLJ/18 acggcctgaaagttcaaaccctccacaatcaccttggacccgatgagaatacgcagctgg 2699

************************************************************

intB318L tggccttccaggttggacgaggcgttaaaaagagccaggcttcgttcgcgtacagcgggc 2760

HLJ/18 tggccttccaggttggacgaggcgttaaaaagagccaggcttcgttcgcgtacagcgggc 2759

************************************************************

intB318L tctatttcgctgtgcagaatggtgaaccgtactggaataaactgatggtcgctatgtgtg 2820

HLJ/18 tctatttcgctgtgcagaatggtgaaccgtactggaataaactgatggtcgctatgtgtg 2819

************************************************************

intB318L tgctcatcgcgaatcgcggcgcagatggagcagcgggtcgttcccacaggggacgaaact 2880

HLJ/18 tgctcatcgcgaatcgcggcgcagatggagcagcgggtcgttcccacaggggacgaaact 2879

************************************************************

intB318L tcatttaaaatgccattactttgtaaaatttcttgcaagataagaacccccgacatgcgg 2940

HLJ/18 tcatttaaaatgccattactttgtaaaatttcttgcaagataagaacccccgacatgcgg 2939

************************************************************

intB318L acccgattgtggtaaattaaaattttcccccggccttgccgaataatggaaagaatgtct 3000

HLJ/18 acccgattgtggtaaattaaaattttcccccggccttgccgaataatggaaagaatgtct 2999

************************************************************

intB318L ttcatcatttgagtgtattttccgctataaaaggccaatcccgagatgtgcgttggtggc 3060

HLJ/18 ttcatcatttgagtgtattttccgctataaaaggccaatcccgagatgtgcgttggtggc 3059

************************************************************

intB318L tgcagcgacaaaaagctgccactcacattaaagggggctctacgcgaaggctcaataatc 3120

HLJ/18 tgcagcgacaaaaagctgccactcacattaaagggggctctacgcgaaggctcaataatc 3119

************************************************************

intB318L tgtaccccgttttccagaagccagtctgtgcttgccatagaaagggcggtgggggtttcc 3180

HLJ/18 tgtaccccgttttccagaagccagtctgtgcttgccatagaaagggcggtgggggtttcc 3179

************************************************************

intB318L gtcgagttaaacaggccgtaagccttgggttccgtttgttttgaaaattttgggttggga 3240

HLJ/18 gtcgagttaaacaggccgtaagccttgggttccgtttgttttgaaaattttgggttggga 3239

************************************************************

intB318L aacaccatgtcataaatgctgtacgcattactcgagattttagggtcagggcccagctgt 3300

HLJ/18 aacaccatgtcataaatgctgtacgcattactcgagattttagggtcagggcccagctgt 3299

************************************************************

intB318L ttaagcgtttcaagctgatactcagacatggggcattcgatgaaatgtaagtacggcaat 3360

HLJ/18 ttaagcgtttcaagctgatactcagacatggggcattcgatgaaatgtaagtacggcaat 3359

************************************************************

intB318L gtttcgtctttataggacaacatctttccggcaaatattctttcggggtaaaaattggtg 3420

HLJ/18 gtttcgtctttataggacaacatctttccggcaaatattctttcggggtaaaaattggtg 3419

************************************************************

intB318L ttggtatccaacaaaaaagatacccttccggtgctcagtctttccacaagagctagggcg 3480

HLJ/18 ttggtatccaacaaaaaagatacccttccggtgctcagtctttccacaagagctagggcg 3479

************************************************************

intB318L tcctttttccatttaacggaatgcccactgctgtcaaacagttgctggcgctggaggggc 3540

HLJ/18 tcctttttccatttaacggaatgcccactgctgtcaaacagttgctggcgctggaggggc 3539

************************************************************

intB318L tggccgttgggcagctcatgccgcggaaccaaaaggtttaacaggtcgacgtattccatg 3600

HLJ/18 tggccgttgggcagctcatgccgcggaaccaaaaggtttaacaggtcgacgtattccatg 3599

************************************************************

intB318L acactcccggttacgggcgttgccgacatgaagacggccctgggggcctggtgaggtgga 3660

HLJ/18 acactcccggttacgggcgttgccgacatgaagacggccctgggggcctggtgaggtgga 3659

************************************************************

intB318L aaggcatccaggacatactgtaaagcgatgccataattatttcgttcctggatattgtac 3720

HLJ/18 aaggcatccaggacatactgtaaagcgatgccataattatttcgttcctggatattgtac 3719

************************************************************

intB318L acgttgtgtatttcatccgcaatgagcagtcctcccctaagttgctccatgattttttga 3780

HLJ/18 acgttgtgtatttcatccgcaatgagcagtcctcccctaagttgctccatgattttttga 3779

************************************************************

intB318L ttcacccggatgaggccgtttgtctcggcctcgctaattttttgcacgaactgagatata 3840

HLJ/18 ttcacccggatgaggccgtttgtctcggcctcgctaattttttgcacgaactgagatata 3839

************************************************************

intB318L tcgttctcattcaatgtatcttctgcttcgtcagaacgatgaaacagagaaagcacatca 3900

HLJ/18 tcgttctcattcaatgtatcttctgcttcgtcagaacgatgaaacagagaaagcacatca 3899

************************************************************

intB318L aagtttttctcttcacccttactcgtaatattgaaaagcttggatgcaaattccttatag 3960

HLJ/18 aagtttttctcttcacccttactcgtaatattgaaaagcttggatgcaaattccttatag 3959

************************************************************

intB318L ccgtaaaactgaaaaaagcctccgcggtttctatcggttaaacggcgctttaacgtacta 4020

HLJ/18 ccgtaaaactgaaaaaagcctccgcggtttctatcggttaaacggcgctttaacgtacta 4019

************************************************************

intB318L acgaacccatttagatgccgtgattcgaccgacgtggtgctgccagactgctttgcaatg 4080

HLJ/18 acgaacccatttagatgccgtgattcgaccgacgtggtgctgccagactgctttgcaatg 4079

************************************************************

intB318L tgaagaagccggtgtagctcagcgacctccttgtaagaaacaaatcccagctcaggacgt 4140

HLJ/18 tgaagaagccggtgtagctcagcgacctccttgtaagaaacaaatcccagctcaggacgt 4139

************************************************************

intB318L cttagcatttctgtttgaatgatggcgcgtgtaaagcctaccacaaaaatccagggcgca 4200

HLJ/18 cttagcatttctgtttgaatgatggcgcgtgtaaagcctaccacaaaaatccagggcgca 4199

************************************************************

intB318L ttttcaataaaattcatgtagtggttcataaattgacgcgcgatggcaatcgcggcaatg 4260

HLJ/18 ttttcaataaaattcatgtagtggttcataaattgacgcgcgatggcaatcgcggcaatg 4259

************************************************************

intB318L ctttttcccgtcccggtctgccagtttaataaaagacgcgagtagggcgtgttgggattt 4320

HLJ/18 ctttttcccgtcccggtctgccagtttaataaaagacgcgagtagggcgtgttgggattt 4319

************************************************************

intB318L tgaaagttttggacgaaaagctgggcattatgcaattggagacccttgatggaaggaaag 4380

HLJ/18 tgaaagttttggacgaaaagctgggcattatgcaattggagacccttgatggaaggaaag 4379

************************************************************

intB318L ggcgacgcgtaggggtcacacggaaaaaacgctcgcccccccttctcgcagccaggccca 4440

HLJ/18 ggcgacgcgtaggggtcacacggaaaaaacgctcgcccccccttctcgcagccaggccca 4439

************************************************************

intB318L ccgatctggacaaaatgagcccgcagatcacgaatgagctctttttggtcgacaggaggg 4500

HLJ/18 ccgatctggacaaaatgagcccgcagatcacgaatgagctctttttggtcgacaggaggg 4499

************************************************************

intB318L gaaatcaacgatttaaactcctttcttcgcgccaactgctgcaaaaagtctgcggcatcc 4560

HLJ/18 gaaatcaacgatttaaactcctttcttcgcgccaactgctgcaaaaagtctgcggcatcc 4559

************************************************************

intB318L aattcgggatacgccat 4577

HLJ/18 aattcgggatacgccat 4576

*****************

# ASFV-HLJ/18 (RED: D339L, GREEN: D1133L)

TTATTCATGTCGGGAGATGGCTATTTAAAAATCATGTCCTATTTTTCTTTGCTCAATAAGCATCCAAATATTTTCATGGCGTTTTATTAATTGTTCATTATTGAACGTATCACAAAGATCATTTATAAATTGCAGATAGTTTATTATTTCTTTCAAGAGAGTAACAAACATTACTTCAGCAGAACATATAATAGGTAATTCAGTGGCGTTAAAAGAATTTTGATCTTGTTGATACGCCAATGGCGAGGACTTAAGGAGATTTGGGGGTCTTGCCCAAAACCCTAGGCTGCTGTTCTTGTTTTTTAGGGCGTCATAAAGAAATGAAAGCACATTGCAAGGCTTAAGCCGCGACATCTCCTTCCCCTTGGGCCCTTTCCATATTTTTAGATCTAAGATCTCATCCGAGCTTATAGAGTAGGTATAGTAAAGTTTTTCAAAAAAGCATATCTGCTTGAAGTCTTTTTTAGAACGACTTTCAAGAAGCATTTCTATAATGTTAACAAGTTTTGTTAGGTTTAAGGCCTGTTCCTGTGTAAGCTCCTCTTGCACGTGATAGACTGAAAAAGTGTGCTTAGGAATGAAAATACTCCCCGTGGCACTGGCCTGTTGTCTGCCAGGTATATAGTACACGCTGCTGTTAGCAAGCTGTACCGGCACAATTTGCCCCACTTCTGCAACATTATTTTGCGATTCGGACGAGGGTATGACAATAGTTACGGGTTCAGTCAATAGGCTTTCGCCGAGAATAATATTACTGTCATTTTTAATAATTTTAACGGCCGCTATTAAATCAAAGGCATTTAAGTAAGAAACAACAGCAGAAAATCTTACATGCATATATCCTCTTCCGCTATTATTCGTACGCATAATAAAACAAGGGGAGCGTTGTATAACGCCAGTAATATTAAGAATAAAACTGTTTTTGAAACACTTACCCACATAAATGTTTTCAAGCTCCTTCAAAAGATGAGCCTCCACATTTGTACAAAAATTGGTAGGATCATCAATATTCAACGTTGTCTCAAAAATTTTTTGGTCGATCATATCTATAATATATTCTGTCTATTTCAATTTAAATAATATACGAATAAATAACGAGATTATTTTATTAAATAAGCAATGGTGTATACACTTTGTATTTACTTTGAGATATACTTTGTGTATCACAACGTGCCCTAAGATGTGTGCACAAGTGACGGCATTTTGTCGTTAAAAAGGTAAAACCAGCGGATTCCATCCTGCATTCCATTTGGTTGATTACGAGCCTCCATTTCTTTTTGCAAAAGGTTATTGCGAATGAGTAAGCAGAGCTTGATGGCACTAATCTTTGTAAGGTTTAAACTTATGCCCAATTGGTCAGCAATTTTTTGTTGCTCCTCCCGTCCGCGTGTTTCGCATACGGCTCCCCGGTTTAGCATGCGAATATCAGTAATCTCATTCTTTTTTAAAACCGGATAGGTGGGCGGATTTTAAATTTAAGGGCCTTTCCCTTGCTTTCCATATAGCCTATGACGATGTCGTTTTCTTTTCGTTTAACATTAATATTAAGCATATAAAGCGGAATTTCATGCCAGGTTTTATCTTCTCGCGAGGTAATAAGTCGCACGGAGTCCTCCGTGGCATAGCCCACTAGAGTGTTGTCATCCCCAGGCACGTGGCTTATAATTTTAAAAATGTCCGGAAATGGCTGAATATCTTTTTTTGAAAAAGCGATGAAAAACTTTTTATAAACCTCGACAAGGGCCCCCATACCTGCAAGATTATCTATAATAAGTGCTTCTAGCATCGTATAGTGAAATGAAGCGGGGTAGTGGATGAGTACCTGCTCCATTGGCTCATCCTGAAAATCCTTCTGAAACTTTTCATACAATACTTGAAAGGGTTCTTTGGTCTGCGAGTGTTCGAGGTATTTGGTAATACGGATGCTGTGCATCGCGGGAGGCTGAAAATCCCGAATATATGTTTCAATATCTAATACCGGTTCCTTTTTATGGTTAAGCACCGCAGCGACGTACAAATGCTCAGGCTTTGCCGGCACATGCATAATGGTGCAAAGACGATTCTGTATCCATAATTCCTTGCACTGGTTTTTTGAGTAGCATAGAGAAATGAGCGCCAGCGCGAAGTTGTCCTCTGAGAAGAGTTTATTATCGATGGTAATTCCCTGTATGAGCTTGGGAGTGGAAACAGCCTTCCATAGCTCGGAGTACGTCCACACGGGGCGTGCCATAAACAAAGATATAATAATATTAGAAATTGTTTTTACCTCTTGCTCCCCGTATCCATAGGCCTCAAAGGTATTGAGGACGGTGGCTCCGACGTTTGCCGGCGTGATGGATGGACTAAGGGGCAGACTTTCCAACATAGGCTTATCAATCTTAATCTGGTTGGTGAACCCATCAATGGCGTGCTTTCGCAGCGCCTTATCCCCCTCCTGTATTAAAATGTATTCTTTTAATTTTTGTGCGTACTTAGCGAGCTCTGGCCCTCCATCGGGTGTTGTCGATACGTACAAATAAATTGTCACGTTGCGCTCACTGGGGGGGAGCTCCATGTGTGAATTTTTTCGCACCACCCTCCCAAATACCTGAATAAGCCGGGGAATATCAAGGGGCAATGACATAATCATCTCGTACCGCACGGCCTGAAAGTTCAAACCCTCCACAATCACCTTGGACCCGATGAGAATACGCAGCTGGTGGCCTTCCAGGTTGGACGAGGCGTTAAAAAGAGCCAGGCTTCGTTCGCGTACAGCGGGCTCTATTTCGCTGTGCAGAATGGTGAACCGTACTGGAATAAACTGATGGTCGCTATGTGTGTGCTCATCGCGAATCGCGGCGCAGATGGAGCAGCGGGTCGTTCCCACAGGGGACGAAACTTCATTTAAAATGCCATTACTTTGTAAAATTTCTTGCAAGATAAGAACCCCCGACATGCGGACCCGATTGTGGTAAATTAAAATTTTCCCCCGGCCTTGCCGAATAATGGAAAGAATGTCTTTCATCATTTGAGTGTATTTTCCGCTATAAAAGGCCAATCCCGAGATGTGCGTTGGTGGCTGCAGCGACAAAAAGCTGCCACTCACATTAAAGGGGGCTCTACGCGAAGGCTCAATAATCTGTACCCCGTTTTCCAGAAGCCAGTCTGTGCTTGCCATAGAAAGGGCGGTGGGGGTTTCCGTCGAGTTAAACAGGCCGTAAGCCTTGGGTTCCGTTTGTTTTGAAAATTTTGGGTTGGGAAACACCATGTCATAAATGCTGTACGCATTACTCGAGATTTTAGGGTCAGGGCCCAGCTGTTTAAGCGTTTCAAGCTGATACTCAGACATGGGGCATTCGATGAAATGTAAGTACGGCAATGTTTCGTCTTTATAGGACAACATCTTTCCGGCAAATATTCTTTCGGGGTAAAAATTGGTGTTGGTATCCAACAAAAAAGATACCCTTCCGGTGCTCAGTCTTTCCACAAGAGCTAGGGCGTCCTTTTTCCATTTAACGGAATGCCCACTGCTGTCAAACAGTTGCTGGCGCTGGAGGGGCTGGCCGTTGGGCAGCTCATGCCGCGGAACCAAAAGGTTTAACAGGTCGACGTATTCCATGACACTCCCGGTTACGGGCGTTGCCGACATGAAGACGGCCCTGGGGGCCTGGTGAGGTGGAAAGGCATCCAGGACATACTGTAAAGCGATGCCATAATTATTTCGTTCCTGGATATTGTACACGTTGTGTATTTCATCCGCAATGAGCAGTCCTCCCCTAAGTTGCTCCATGATTTTTTGATTCACCCGGATGAGGCCGTTTGTCTCGGCCTCGCTAATTTTTTGCACGAACTGAGATATATCGTTCTCATTCAATGTATCTTCTGCTTCGTCAGAACGATGAAACAGAGAAAGCACATCAAAGTTTTTCTCTTCACCCTTACTCGTAATATTGAAAAGCTTGGATGCAAATTCCTTATAGCCGTAAAACTGAAAAAAGCCTCCGCGGTTTCTATCGGTTAAACGGCGCTTTAACGTACTAACGAACCCATTTAGATGCCGTGATTCGACCGACGTGGTGCTGCCAGACTGCTTTGCAATGTGAAGAAGCCGGTGTAGCTCAGCGACCTCCTTGTAAGAAACAAATCCCAGCTCAGGACGTCTTAGCATTTCTGTTTGAATGATGGCGCGTGTAAAGCCTACCACAAAAATCCAGGGCGCATTTTCAATAAAATTCATGTAGTGGTTCATAAATTGACGCGCGATGGCAATCGCGGCAATGCTTTTTCCCGTCCCGGTCTGCCAGTTTAATAAAAGACGCGAGTAGGGCGTGTTGGGATTTTGAAAGTTTTGGACGAAAAGCTGGGCATTATGCAATTGGAGACCCTTGATGGAAGGAAAGGGCGACGCGTAGGGGTCACACGGAAAAAACGCTCGCCCCCCCTTCTCGCAGCCAGGCCCACCGATCTGGACAAAATGAGCCCGCAGATCACGAATGAGCTCTTTTTGGTCGACAGGAGGGGAAATCAACGATTTAAACTCCTTTCTTCGCGCCAACTGCTGCAAAAAGTCTGCGGCATCCAATTCGGGATACGCCAT

# ASFV-intB318L (RED: D339L, GREEN: D1133L of HLJ/18, Gray background: D1133L of Georgia)

TTATTCATGTCGGGAGATGGCTATTTAAAAATCATGTCCTATTTTTCTTTGCTCAATAAGCATCCAAATATTTTCATGGCGTTTTATTAATTGTTCATTATTGAACGTATCACAAAGATCATTTATAAATTGCAGATAGTTTATTATTTCTTTCAAGAGAGTAACAAACATTACTTCAGCAGAACATATAATAGGTAATTCAGTGGCGTTAAAAGAATTTTGATCTTGTTGATACGCCAATGGCGAGGACTTAAGGAGATTTGGGGGTCTTGCCCAAAACCCTAGGCTGCTGTTCTTGTTTTTTAGGGCGTCATAAAGAAATGAAAGCACATTGCAAGGCTTAAGCCGCGACATCTCCTTCCCCTTGGGCCCTTTCCATATTTTTAGATCTAAGATCTCATCCGAGCTTATAGAGTAGGTATAGTAAAGTTTTTCAAAAAAGCATATCTGCTTGAAGTCTTTTTTAGAACGACTTTCAAGAAGCATTTCTATAATGTTAACAAGTTTTGTTAGGTTTAAGGCCTGTTCCTGTGTAAGCTCCTCTTGCACGTGATAGACTGAAAAAGTGTGCTTAGGAATGAAAATACTCCCCGTGGCACTGGCCTGTTGTCTGCCAGGTATATAGTACACGCTGCTGTTAGCAAGCTGTACCGGCACAATTTGCCCCACTTCTGCAACATTATTTTGCGATTCGGACGAGGGTATGACAATAGTTACGGGTTCAGTCAATAGGCTTTCGCCGAGAATAATATTACTGTCATTTTTAATAATTTTAACGGCCGCTATTAAATCAAAGGCATTTAAGTAAGAAACAACAGCAGAAAATCTTACATGCATATATCCTCTTCCGCTATTATTCGTACGCATAATAAAACAAGGGGAGCGTTGTATAACGCCAGTAATATTAAGAATAAAACTGTTTTTGAAACACTTACCCACATAAATGTTTTCAAGCTCCTTCAAAAGATGAGCCTCCACATTTGTACAAAAATTGGTAGGATCATCAATATTCAACGTTGTCTCAAAAATTTTTTGGTCGATCATATCTATAATATATTCTGTCTATTTCAATTTAAATAATATACGAATAAATAACGAGATTATTTTATTAAATAAGCAATGGTGTATACACTTTGTATTTACTTTGAGATATACTTTGTGTATCACAACGTGCCCTAAGATGTGTGCACAAGTGACGGCATTTTGTCGTTAAAAAGGTAAAACCAGCGGATTCCATCCTGCATTCCATTTGGTTGATTACGAGCCTCCATTTCTTTTTGCAAAAGGTTATTGCGAATGAGTAAGCAGAGCTTGATGGCACTAATCTTTGTAAGGTTTAAACTTATGCCCAATTGGTCAGCAATTTTTTGTTGCTCCTCCCGTCCGCGTGTTTCGCATACGGCTCCCCGGTTTAGCATGCGAATATCAGTAATCTCATTCTTTTTTAAAACCTGGATAGGTGGGCGGATTTTAAATTTAAGGGCCTTTCCCTTGCTTTCCATATAGCCTATGACGATGTCGTTTTCTTTTCGTTTAACATTAATATTAAGCATATAAAGCGGAATTTCATGCCAGGTTTTATCTTCTCGCGAGGTAATAAGTCGCACGGAGTCCTCCGTGGCATAGCCCACTAGAGTGTTGTCATCCCCAGGCACGTGGCTTATAATTTTAAAAATGTCCGGAAATGGCTGAATATCTTTTTTTGAAAAAGCGATGAAAAACTTTTTATAAACCTCGACAAGGGCCCCCATACCTGCAAGATTATCTATAATAAGTGCTTCTAGCATCGTATAGTGAAATGAAGCGGGGTAGTGGATGAGTACCTGCTCCATTGGCTCATCCTGAAAATCCTTCTGAAACTTTTCATACAATACTTGAAAGGGTTCTTTGGTCTGCGAGTGTTCGAGGTATTTGGTAATACGGATGCTGTGCATCGCGGGAGGCTGAAAATCCCGAATATATGTTTCAATATCTAATACCGGTTCCTTTTTATGGTTAAGCACCGCAGCGACGTACAAATGCTCAGGCTTTGCCGGCACATGCATAATGGTGCAAAGACGATTCTGTATCCATAATTCCTTGCACTGGTTTTTTGAGTAGCATAGAGAAATGAGCGCCAGCGCGAAGTTGTCCTCTGAGAAGAGTTTATTATCGATGGTAATTCCCTGTATGAGCTTGGGAGTGGAAACAGCCTTCCATAGCTCGGAGTACGTCCACACGGGGCGTGCCATAAACAAAGATATAATAATATTAGAAATTGTTTTTACCTCTTGCTCCCCGTATCCATAGGCCTCAAAGGTATTGAGGACGGTGGCTCCGACGTTTGCCGGCGTGATGGATGGACTAAGGGGCAGACTTTCCAACATAGGCTTATCAATCTTAATCTGGTTGGTGAACCCATCAATGGCGTGCTTTCGCAGCGCCTTATCCCCCTCCTGTATTAAAATGTATTCTTTTAATTTTTGTGCGTACTTAGCGAGCTCTGGCCCTCCATCGGGTGTTGTCGATACGTACAAATAAATTGTCACGTTGCGCTCACTGGGGGGGAGCTCCATGTGTGAATTTTTTCGCACCACCCTCCCAAATACCTGAATAAGCCGGGGAATATCAAGGGGCAATGACATAATCATCTCGTACCGCACGGCCTGAAAGTTCAAACCCTCCACAATCACCTTGGACCCGATGAGAATACGCAGCTGGTGGCCTTCCAGGTTGGACGAGGCGTTAAAAAGAGCCAGGCTTCGTTCGCGTACAGCGGGCTCTATTTCGCTGTGCAGAATGGTGAACCGTACTGGAATAAACTGATGGTCGCTATGTGTGTGCTCATCGCGAATCGCGGCGCAGATGGAGCAGCGGGTCGTTCCCACAGGGGACGAAACTTCATTTAAAATGCCATTACTTTGTAAAATTTCTTGCAAGATAAGAACCCCCGACATGCGGACCCGATTGTGGTAAATTAAAATTTTCCCCCGGCCTTGCCGAATAATGGAAAGAATGTCTTTCATCATTTGAGTGTATTTTCCGCTATAAAAGGCCAATCCCGAGATGTGCGTTGGTGGCTGCAGCGACAAAAAGCTGCCACTCACATTAAAGGGGGCTCTACGCGAAGGCTCAATAATCTGTACCCCGTTTTCCAGAAGCCAGTCTGTGCTTGCCATAGAAAGGGCGGTGGGGGTTTCCGTCGAGTTAAACAGGCCGTAAGCCTTGGGTTCCGTTTGTTTTGAAAATTTTGGGTTGGGAAACACCATGTCATAAATGCTGTACGCATTACTCGAGATTTTAGGGTCAGGGCCCAGCTGTTTAAGCGTTTCAAGCTGATACTCAGACATGGGGCATTCGATGAAATGTAAGTACGGCAATGTTTCGTCTTTATAGGACAACATCTTTCCGGCAAATATTCTTTCGGGGTAAAAATTGGTGTTGGTATCCAACAAAAAAGATACCCTTCCGGTGCTCAGTCTTTCCACAAGAGCTAGGGCGTCCTTTTTCCATTTAACGGAATGCCCACTGCTGTCAAACAGTTGCTGGCGCTGGAGGGGCTGGCCGTTGGGCAGCTCATGCCGCGGAACCAAAAGGTTTAACAGGTCGACGTATTCCATGACACTCCCGGTTACGGGCGTTGCCGACATGAAGACGGCCCTGGGGGCCTGGTGAGGTGGAAAGGCATCCAGGACATACTGTAAAGCGATGCCATAATTATTTCGTTCCTGGATATTGTACACGTTGTGTATTTCATCCGCAATGAGCAGTCCTCCCCTAAGTTGCTCCATGATTTTTTGATTCACCCGGATGAGGCCGTTTGTCTCGGCCTCGCTAATTTTTTGCACGAACTGAGATATATCGTTCTCATTCAATGTATCTTCTGCTTCGTCAGAACGATGAAACAGAGAAAGCACATCAAAGTTTTTCTCTTCACCCTTACTCGTAATATTGAAAAGCTTGGATGCAAATTCCTTATAGCCGTAAAACTGAAAAAAGCCTCCGCGGTTTCTATCGGTTAAACGGCGCTTTAACGTACTAACGAACCCATTTAGATGCCGTGATTCGACCGACGTGGTGCTGCCAGACTGCTTTGCAATGTGAAGAAGCCGGTGTAGCTCAGCGACCTCCTTGTAAGAAACAAATCCCAGCTCAGGACGTCTTAGCATTTCTGTTTGAATGATGGCGCGTGTAAAGCCTACCACAAAAATCCAGGGCGCATTTTCAATAAAATTCATGTAGTGGTTCATAAATTGACGCGCGATGGCAATCGCGGCAATGCTTTTTCCCGTCCCGGTCTGCCAGTTTAATAAAAGACGCGAGTAGGGCGTGTTGGGATTTTGAAAGTTTTGGACGAAAAGCTGGGCATTATGCAATTGGAGACCCTTGATGGAAGGAAAGGGCGACGCGTAGGGGTCACACGGAAAAAACGCTCGCCCCCCCTTCTCGCAGCCAGGCCCACCGATCTGGACAAAATGAGCCCGCAGATCACGAATGAGCTCTTTTTGGTCGACAGGAGGGGAAATCAACGATTTAAACTCCTTTCTTCGCGCCAACTGCTGCAAAAAGTCTGCGGCATCCAATTCGGGATACGCCAT

**ASFV-Georgia 2007/1** (RED: D339L, GREEN: D1133L)

TATTCATGTCGGGAGATGGCTATTTAAAAATCATGTCCTATTTTTCTTTGCTCAATAAGCATCCAAATATTTTCATGGCGTTTTATTAATTGTTCATTATTGAACGTATCACAAAGATCATTTATAAATTGCAGATAGTTTATTATTTCTTTCAAGAGAGTAACAAACATTACTTCAGCAGAACATATAATAGGTAATTCAGTGGCGTTAAAAGAATTTTGATCTTGTTGATACGCCAATGGCGAGGACTTAAGGAGATTTGGGGGTCTTGCCCAAAACCCTAGGCTGCTGTTCTTGTTTTTTAGGGCGTCATAAAGAAATGAAAGCACATTGCAAGGCTTAAGCCGCGACATCTCCTTCCCCTTGGGCCCTTTCCATATTTTTAGATCTAAGATCTCATCCGAGCTTATAGAGTAGGTATAGTAAAGTTTTTCAAAAAAGCATATCTGCTTGAAGTCTTTTTTAGAACGACTTTCAAGAAGCATTTCTATAATGTTAACAAGTTTTGTTAGGTTTAAGGCCTGTTCCTGTGTAAGCTCCTCTTGCACGTGATAGACTGAAAAAGTGTGCTTAGGAATGAAAATACTCCCCGTGGCACTGGCCTGTTGTCTGCCAGGTATATAGTACACGCTGCTGTTAGCAAGCTGTACCGGCACAATTTGCCCCACTTCTGCAACATTATTTTGCGATTCGGACGAGGGTATGACAATAGTTACGGGTTCAGTCAATAGGCTTTCGCCGAGAATAATATTACTGTCATTTTTAATAATTTTAACGGCCGCTATTAAATCAAAGGCATTTAAGTAAGAAACAACAGCAGAAAATCTTACATGCATATATCCTCTTCCGCTATTATTCGTACGCATAATAAAACAAGGGGAGCGTTGTATAACGCCAGTAATATTAAGAATAAAACTGTTTTTGAAACACTTACCCACATAAATGTTTTCAAGCTCCTTCAAAAGATGAGCCTCCACATTTGTACAAAAATTGGTAGGATCATCAATATTCAACGTTGTCTCAAAAATTTTTTGGTCGATCATATCTATAATATATTCTGTCTATTTCAATTTAAATAATATACGAATAAATAACGAGATTATTTTATTAAATAAGCAATGGTGTATACACTTTGTATTTACTTTGAGATATACTTTGTGTATCACAACGTGCCCTAAGATGTGTGCACAAGTGACGGCATTTTGTCGTTAAAAAGGTAAAACCAGCGGATTCCATCCTGCATTCCATTTGGTTGATTACGAGCCTCCATTTCTTTTTGCAAAAGGTTATTGCGAATGAGTAAGCAGAGCTTGATGGCACTAATCTTTGTAAGGTTTAAACTTATGCCCAATTGGTCAGCAATTTTTTGTTGCTCCTCCCGTCCGCGTGTTTCGCATACGGCTCCCCGGTTTAGCATGCGAATATCAGTAATCTCATTCTTTTTTAAAACCTGGATAGGTGGGCGGATTTTAAATTTAAGGGCCTTTCCCTTGCTTTCCATATAGCCTATGACGATGTCGTTTTCTTTTCGTTTAACATTAATATTAAGCATATAAAGCGGAATTTCATGCCAGGTTTTATCTTCTCGCGAGGTAATAAGTCGCACGGAGTCCTCCGTGGCATAGCCCACTAGAGTGTTGTCATCCCCAGGCACGTGGCTTATAATTTTAAAAATGTCCGGAAATGGCTGAATATCTTTTTTTGAAAAAGCGATGAAAAACTTTTTATAAACCTCGACAAGGGCCCCCATACCTGCAAGATTATCTATAATAAGTGCTTCTAGCATCGTATAGTGAAATGAAGCGGGGTAGTGGATGAGTACCTGCTCCATTGGCTCATCCTGAAAATCCTTCTGAAACTTTTCATACAATACTTGAAAGGGTTCTTTGGTCTGCGAGTGTTCGAGGTATTTGGTAATACGGATGCTGTGCATCGCGGGAGGCTGAAAATCCCGAATATATGTTTCAATATCTAATACCGGTTCCTTTTTATGGTTAAGCACCGCAGCGACGTACAAATGCTCAGGCTTTGCCGGCACATGCATAATGGTGCAAAGACGATTCTGTATCCATAATTCCTTGCACTGGTTTTTTGAGTAGCATAGAGAAATGAGCGCCAGCGCGAAGTTGTCCTCTGAGAAGAGTTTATTATCGATGGTAATTCCCTGTATGAGCTTGGGAGTGGAAACAGCCTTCCATAGCTCGGAGTACGTCCACACGGGGCGTGCCATAAACAAAGATATAATAATATTAGAAATTGTTTTTACCTCTTGCTCCCCGTATCCATAGGCCTCAAAGGTATTGAGGACGGTGGCTCCGACGTTTGCCGGCGTGATGGATGGACTAAGGGGCAGACTTTCCAACATAGGCTTATCAATCTTAATCTGGTTGGTGAACCCATCAATGGCGTGCTTTCGCAGCGCCTTATCCCCCTCCTGTATTAAAATGTATTCTTTTAATTTTTGTGCGTACTTAGCGAGCTCTGGCCCTCCATCGGGTGTTGTCGATACGTACAAATAAATTGTCACGTTGCGCTCACTGGGGGGGAGCTCCATGTGTGAATTTTTTCGCACCACCCTCCCAAATACCTGAATAAGCCGGGGAATATCAAGGGGCAATGACATAATCATCTCGTACCGCACGGCCTGAAAGTTCAAACCCTCCACAATCACCTTGGACCCGATGAGAATACGCAGCTGGTGGCCTTCCAGGTTGGACGAGGCGTTAAAAAGAGCCAGGCTTCGTTCGCGTACAGCGGGCTCTATTTCGCTGTGCAGAATGGTGAACCGTACTGGAATAAACTGATGGTCGCTATGTGTGTGCTCATCGCGAATCGCGGCGCAGATGGAGCAGCGGGTCGTTCCCACAGGGGACGAAACTTCATTTAAAATGCCATTACTTTGTAAAATTTCTTGCAAGATAAGAACCCCCGACATGCGGACCCGATTGTGGTAAATTAAAATTTTCCCCCGGCCTTGCCGAATAATGGAAAGAATGTCTTTCATCATTTGAGTGTATTTTCCGCTATAAAAGGCCAATCCCGAGATGTGCGTTGGTGGCTGCAGCGACAAAAAGCTGCCACTCACATTAAAGGGGGCTCTACGCGAAGGCTCAATAATCTGTACCCCGTTTTCCAGAAGCCAGTCTGTGCTTGCCATAGAAAGGGCGGTGGGGGTTTCCGTCGAGTTAAACAGGCCGTAAGCCTTGGGTTCCGTTTGTTTTGAAAATTTTGGGTTGGGAAACACCATGTCATAAATGCTGTACGCATTACTCGAGATTTTAGGGTCAGGGCCCAGCTGTTTAAGCGTTTCAAGCTGATACTCAGACATGGGGCATTCGATGAAATGTAAGTACGGCAATGTTTCGTCTTTATAGGACAACATCTTTCCGGCAAATATTCTTTCGGGGTAAAAATTGGTGTTGGTATCCAACAAAAAAGATACCCTTCCGGTGCTCAGTCTTTCCACAAGAGCTAGGGCGTCCTTTTTCCATTTAACGGAATGCCCACTGCTGTCAAACAGTTGCTGGCGCTGGAGGGGCTGGCCGTTGGGCAGCTCATGCCGCGGAACCAAAAGGTTTAACAGGTCGACGTATTCCATGACACTCCCGGTTACGGGCGTTGCCGACATGAAGACGGCCCTGGGGGCCTGGTGAGGTGGAAAGGCATCCAGGACATACTGTAAAGCGATGCCATAATTATTTCGTTCCTGGATATTGTACACGTTGTGTATTTCATCCGCAATGAGCAGTCCTCCCCTAAGTTGCTCCATGATTTTTTGATTCACCCGGATGAGGCCGTTTGTCTCGGCCTCGCTAATTTTTTGCACGAACTGAGATATATCGTTCTCATTCAATGTATCTTCTGCTTCGTCAGAACGATGAAACAGAGAAAGCACATCAAAGTTTTTCTCTTCACCCTTACTCGTAATATTGAAAAGCTTGGATGCAAATTCCTTATAGCCGTAAAACTGAAAAAAGCCTCCGCGGTTTCTATCGGTTAAACGGCGCTTTAACGTACTAACGAACCCATTTAGATGCCGTGATTCGACCGACGTGGTGCTGCCAGACTGCTTTGCAATGTGAAGAAGCCGGTGTAGCTCAGCGACCTCCTTGTAAGAAACAAATCCCAGCTCAGGACGTCTTAGCATTTCTGTTTGAATGATGGCGCGTGTAAAGCCTACCACAAAAATCCAGGGCGCATTTTCAATAAAATTCATGTAGTGGTTCATAAATTGACGCGCGATGGCAATCGCGGCAATGCTTTTTCCCGTCCCGGTCTGCCAGTTTAATAAAAGACGCGAGTAGGGCGTGTTGGGATTTTGAAAGTTTTGGACGAAAAGCTGGGCATTATGCAATTGGAGACCCTTGATGGAAGGAAAGGGCGACGCGTAGGGGTCACACGGAAAAAACGCTCGCCCCCCCTTCTCGCAGCCAGGCCCACCGATCTGGACAAAATGAGCCCGCAGATCACGAATGAGCTCTTTTTGGTCGACAGGAGGGGAAATCAACGATTTAAACTCCTTTCTTCGCGCCAACTGCTGCAAAAAGTCTGCGGCATCCAATTCGGGATACGCCAT
